# Design, Synthesis, and Anti-Biofilm Activity of C-28 Modified Betulinic Acid Derivatives Targeting SarA in Drug-Resistant *Staphylococcus aureus*

**DOI:** 10.3390/microorganisms14030574

**Published:** 2026-03-03

**Authors:** Dongshun Jia, Junchao Zhang, Xuejin Zhang, Peng Gao, Hongyu Zhan, Zihan Dong, Hao Li, Fanhao Meng, Nan Cai, Dajun Zhang

**Affiliations:** 1 School of Pharmacy, Shenyang Medical College, Shenyang 110034, China; 2 School of Pharmacy, China Medical University, Shenyang 110034, China; 3 Faculty of Dentistry, The University of Hong Kong, Hong Kong 999077, China

**Keywords:** betulinic acid, antibacterial activity, biofilm inhibition, molecular docking

## Abstract

To address the urgent challenge of antimicrobial resistance, a series of twenty novel C-28 modified betulinic acid derivatives was designed and synthesized. Several derivatives, particularly **3b**, **3d**, **3e**, and **3o**, displayed notable antibacterial activity against Gram-positive bacteria, including *Staphylococcus aureus* and vancomycin-resistant *Staphylococcus aureus* (VRSA). The most active compound, **3d**, was subjected to further mechanistic evaluation: it produced concentration-dependent inhibition zones in Oxford cup assays, exhibited bactericidal kinetics in time-kill studies, and significantly suppressed biofilm formation. Molecular docking suggested that the anti-biofilm activity of **3d** may be mediated through binding to the staphylococcal accessory regulator A (SarA), a key transcriptional regulator of biofilm formation. The molecular dynamics study provided additional confirmation of the effective binding between **3d** and SarA. These results highlight compound **3d** as a promising lead for the development of novel anti-biofilm agents targeting drug-resistant Gram-positive infections.

## 1. Introduction

Pathogenic bacterial infections have long posed a significant threat to global public health, causing millions of deaths annually and substantially increasing the global disease burden [1]. The serendipitous discovery of penicillin by Fleming in 1928 ushered in the golden age of antibiotics [2], revolutionizing medical practice and enabling complex interventions such as organ transplantation and chemotherapy. However, the widespread clinical and agricultural use of antibiotics since the 1940s has accelerated the emergence of antimicrobial resistance (AMR) [3,4]. Today, AMR represents a critical 21st-century global health challenge [5], driven by horizontal gene transfer and genomic mutations in pathogens [6].

Multidrug-resistant (MDR) and extensively drug-resistant (XDR) “superbugs” now propagate globally, rendering conventional antibiotics ineffective against common infections (e.g., urinary tract infections, sepsis). According to WHO surveillance, AMR-associated infections cause >2.3 million deaths annually in North America and >33,000 direct deaths in Europe [7]. A 2019 systematic analysis attributed 4.95 million deaths to drug-resistant infections, with *S. aureus*, *E. coli*, *K. pneumoniae*, *S. pneumoniae*, *A. baumannii*, and *P. aeruginosa* accounting for 929,000 fatalities [8]. Without intervention, AMR-related deaths may reach 10 million per year by 2050, with projected economic losses of $100 trillion [9,10].

The antibiotic development pipeline remains alarmingly scarce: only 42 candidates were in clinical trials as of 2019, with merely 4 exhibiting novel mechanisms [11]. This crisis stems from three key factors: (1) high costs and rapid resistance development during antibiotic R&D, (2) technological bottlenecks in discovering new scaffolds, and (3) persistent global antibiotic misuse [11].

Natural products and their derivatives have historically served as cornerstones for novel drug discovery [12,13,14]. Plant-derived metabolites exhibit diverse pharmacological properties, including antimicrobial [15], antiviral [16], and antitumor activities [17]. Notably, artemisinin (antimalarial) and vinblastine (anticancer) exemplify successful natural product-derived therapeutics [18,19]. Plant antimicrobials—particularly pentacyclic triterpenoids—offer structurally unique, low-toxicity scaffolds against resistant pathogens [20,21,22]. Betulinic acid (BA), a lupane-type triterpenoid widely distributed in Betula species, demonstrates broad bioactivities, including antibacterial effects [22,23]. Although native BA exhibits moderate antimicrobial potency [24], its low eukaryotic cytotoxicity and modifiable C-3/C-28 positions render it an ideal scaffold for structural optimization [25,26,27]. Recent studies confirm that BA derivatives disrupt bacterial membrane integrity and inhibit metabolic pathways [28,29], while C-28 modifications enhance activity against Gram-positive pathogens and fungi [26,27].

The C-28 carboxyl group of betulinic acid provides a versatile site for chemical modification to enhance its bioactivity. In this study, aniline, piperazine, and aminoheterocyclic moieties were strategically selected for conjugation based on a reasoned design approach. Aniline derivatives—particularly those substituted with halogen atoms (F, Cl, Br, and CF_3_)—were chosen because electron-withdrawing groups are known to modulate molecular lipophilicity and electronic properties, which can improve antibacterial potency in triterpenoid scaffolds. Furthermore, piperazine and aminoheterocycles such as thiazole and pyridine were incorporated to introduce nitrogen-rich, polar fragments. This modification aims not only to enhance molecular solubility but also to promote hydrogen-bonding interactions with biological targets, which are often critical in anti-biofilm contexts involving protein–protein or protein–DNA interfaces, including transcriptional regulators like SarA. By integrating these distinct pharmacophores through a flexible chloroacetyl chloride linker, we sought to construct a hybrid compound library that balances the lipophilic character contributed by the BA core and aryl groups with added polar functionality, thereby optimizing the derivatives for dual antibacterial and biofilm-inhibitory effects.

**Figure 1:**
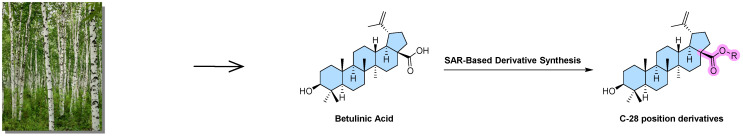
Design strategy for the target compounds.

Herein, to address the urgent need for innovative anti-infective agents, we designed a series of novel BA derivatives by introducing diverse aniline, piperazine, and aminoheterocyclic moieties at the C-28 position via a chloroacetyl chloride linker. The primary objectives of this study were to (1) systematically evaluate the antibacterial potency of these derivatives against a panel of clinically relevant Gram-positive and Gram-negative pathogens, with a focus on drug-resistant Staphylococcus aureus; (2) investigate their potential in inhibiting bacterial biofilm formation, a key virulence factor associated with persistent infections; and (3) preliminarily explore the underlying mechanism, specifically whether the anti-biofilm activity involves interference with key regulatory proteins such as the staphylococcal accessory regulator A (SarA). Twenty derivatives were synthesized, characterized, and evaluated against six clinically relevant pathogens to address the urgent need for innovative anti-infective agents (Figure 1).

## 2. Methods and Materials

### 2.1. Chemistry

#### 2.1.1. General Procedure for Synthesis of Intermediate Amides **2**

To a dried glass reactor, substituted aniline (20.0 mmol) and dichloromethane (DCM, 60 mL) were added and stirred at room temperature until complete dissolution. The reaction mixture was then cooled to −5 °C, followed by dropwise addition of triethylamine (3.5 mL). After stirring for 30 min, chloroacetyl chloride (24.0 mmol) dissolved in DCM (30 mL) was added slowly via dropping funnel over 30 min. The reaction was maintained under vigorous stirring for 12 h at −5 °C. Reaction completion was monitored by thin-layer chromatography. The mixture was sequentially washed with saturated NaCl solution (3 × 50 mL) and distilled water (3 × 50 mL). The organic phase was dried over anhydrous Na_2_SO_4_, concentrated in vacuo, and purified by column chromatography on silica gel (200–300 mesh) using a gradient eluent to afford pure intermediate amides **2**.

#### 2.1.2. General Procedure for Synthesis of Target Compounds **3**

In a round-bottom flask, betulinic acid (1.0 mmol) was dissolved in acetonitrile (30 mL) under magnetic stirring at ambient temperature. Potassium carbonate (3.0 mmol) was added, and the mixture was heated to reflux for 1 h. A solution of intermediate amide **2** (1.5 mmol) in acetonitrile (10 mL) was then introduced dropwise, and refluxing was continued for 12 h. Progress was monitored by thin-layer chromatography (TLC). After completion, the reaction mixture was allowed to cool to room temperature, filtered under vacuum, and concentrated in vacuo. The residue was purified by column chromatography to afford the target compounds **3**. The characterization data of target compounds **3a**–**3t** are provided in the Supplementary Materials.

### 2.2. Bioassay

All strains (*Staphylococcus aureus* ATCC 25923, vancomycin-resistant *Staphylococcus aureus* (VRSA) clinical isolate (provided by the Faculty of Dentistry, The University of Hong Kong), *Streptococcus pneumoniae* ATCC 49619, *Staphylococcus epidermidis* ATCC 12228, *Escherichia coli* ATCC 25922, and *Pseudomonas aeruginosa* ATCC 27853) were obtained from the American Type Culture Collection (ATCC) or clinically isolated and preserved in our laboratory.

#### 2.2.1. Determination of Minimum Inhibitory Concentrations (MICs)

Based on methods described in the literature, the assay was performed with slight adaptations [30]. The test compounds were dissolved in dimethyl sulfoxide (DMSO), with the final concentration kept below 1% (*v*/*v*). Levofloxacin and tetracycline (8 × MIC) were used as positive controls, while the solvent DMSO (1%) served as a blank control. The stock solutions were filter-sterilized using a 0.22 μm membrane filter. A sterile 96-well plate was loaded with culture medium and test drug solutions in sequence, yielding a total volume of 100 μL per well. Each well was then supplemented with 100 μL of bacterial suspension (1.5 × 10^6^ CFU/mL) and mixed thoroughly. The final concentrations of the test compounds in the wells were 250, 125, 62.5, 31.25, 15.63, 7.81, 3.91, 1.95, 0.98, and 0.49 μg/mL. The plates were incubated at 37 °C for 24 h, after which the optical density at 600 nm (OD_600_) was measured using a microplate reader. All experiments were conducted in triplicate.

#### 2.2.2. Antibacterial Activity Evaluation Using the Oxford Cup Method

The experimental procedure was adapted from established methods reported in the literature [30], with minor modifications. Under a laminar flow hood, 100 μL aliquots of bacterial suspensions of *S. aureus* and VRSA (1.5 × 10^6^ CFU/mL) were separately spread onto sterile agar plates. Each inoculum was evenly distributed using a sterile cotton swab by rotating the plate 60° between three consecutive spreads, following a circular motion along the plate periphery. A sterilized Oxford cup was then aseptically placed on the agar surface using sterile forceps to ensure full contact. Each cup was filled with 200 μL of the test compound at 8 × MIC, while an equal volume of solvent was used as the negative control. The plates were incubated at 37 °C for 24 h, after which the inhibition zone diameters were measured. The concentration of 8 × MIC was employed to align with established experimental protocols for assessing anti-biofilm and bactericidal activities, serving primarily for mechanistic investigation rather than as a direct therapeutic recommendation. The entire experiment was performed in triplicate.

#### 2.2.3. Evaluation of Time-Dependent Killing Kinetics

The experimental protocol was implemented with minor modifications based on previously reported methodologies [30]. Single colonies of *S. aureus* and VRSA were individually inoculated into brain heart infusion (BHI) broth and cultured at 37 °C with shaking (e.g., 200 rpm) for 16–18 h. The resulting bacterial suspensions were adjusted to approximately 1.5 × 10^6^ CFU/mL using sterile broth. Test compound **3d** and tetracycline were dissolved in DMSO and diluted with distilled water to achieve 8 × MIC stock solutions. The control group contained 1% DMSO.

For the time-kill assay, twenty-one sterile 5 mL tubes were allocated into three sets: negative control, sample, and positive control, corresponding to time points of 0, 0.5, 1, 2, 4, 8, and 24 h. The reaction mixtures were prepared as follows: the negative control contained 1 mL of bacterial suspension and 1 mL of broth; the sample group contained 1 mL of bacterial suspension and 1 mL of the 8 × MIC test compound; the positive control contained 1 mL of bacterial suspension and 1 mL of the 8 × MIC tetracycline. All tubes were incubated at 37 °C. At each designated time point, 100 μL aliquots were withdrawn from the tubes, serially diluted (10^−1^ to 10^−7^) in sterile broth, and spread onto agar plates. After incubation at 37 °C for 24 h, viable colonies were enumerated from plates yielding 30–300 colonies. All experiments were performed in duplicate.

#### 2.2.4. Crystal Violet Assay for Biofilm Formation Inhibition

Following established literature procedures, the assay was conducted with necessary adaptations [30]. To assess biofilm formation, *S. aureus* and VRSA suspensions (100 μL) were co-incubated with 100 μL of compound **3d** (8 × MIC) in a 96-well plate; sterile BHI containing 1% DMSO was used as a blank control, with both conditions replicated three times. Following incubation at 37 °C for 4, 20, and 24 h, planktonic cells were removed by gentle aspiration. The adherent biofilm was washed three times with sterile distilled water to remove non-adherent bacteria. The biofilm was then fixed with 200 μL of 99% methanol for 15 min, followed by air-drying. Subsequently, the biofilm was stained with 1% crystal violet for 15 min, washed again three times with distilled water to remove excess dye, and air-dried. The bound crystal violet was solubilized with 30% acetic acid, and absorbance was measured at 570 nm. All biofilm inhibition experiments were performed in triplicate (n = 3), with data presented as mean ± standard error of the mean (SEM).

#### 2.2.5. Molecular Docking

The molecular docking was performed using AutoDock Vina 1.5.6. After docking, the conformation with the highest frequency of occurrence and the most favorable binding affinity was selected as the final output. Visualization and analysis were carried out using PyMol 3.1 and Discovery Studio (DS) 2019. The selected ligand (**3d**) was subjected to energy minimization to reduce the overall energy of the system and achieve a stable state. The optimized ligand structure was saved in PDB format using DS. The protein file was imported into PyMol, where non-essential ligands and water molecules were removed, and the resulting structure was saved in PDB format for further use. Hydrogen atoms and partial charges were added to the protein, which was then designated as the receptor and saved in PDBQT format. Similarly, the small molecule was processed by adding hydrogens and charges, assigned as the ligand, and its torsion angles and rotatable bonds were checked before saving in PDBQT format. The grid box was set to encompass the entire protein to facilitate comprehensive ligand binding. Following the grid configuration, 50 independent molecular docking runs were performed using AutoDock Tools 1.5.6. Upon completion of the docking process, the conformation with the highest recurrence and the strongest binding affinity was selected as the representative result. The three-dimensional structure and surface model were visualized using DS or PyMol.

#### 2.2.6. Molecular Dynamics (MD) Simulation

A 100 ns molecular dynamics (MD) simulation was performed on the protein–ligand complex using GROMACS 2025. The protein was described with the AMBER99SB-ILDN force field, while the ligand topology was generated using the GAFF2 force field. The protein and ligand topologies were merged, ensuring consistency in atom types. Periodic boundary conditions (PBC) were applied, and the complex was placed in a cubic box with a minimum distance of 1.2 nm between the solute and the box edges. The system was solvated using the TIP3P water model and neutralized with Na^+^/Cl^−^ ions at a physiological concentration of 0.15 M. Energy minimization was first carried out, followed by a two-step equilibration procedure: 1,000,000 steps of NVT (constant particle number, volume, and temperature) and NPT (constant particle number, pressure, and temperature) equilibration, each conducted for 2 ns with a coupling constant of 0.1 ps. The production MD simulation was then run for 50,000,000 steps using a 2 fs time step, resulting in a total simulation time of 100 ns under constant temperature (310 K) and pressure (1 bar). Trajectory data were saved every 1000 steps for subsequent analysis.

The binding free energy after system stabilization was calculated using the MM-PBSA method. The following properties were computed using GROMACS utilities: root-mean-square deviation (RMSD, for overall structural stability), root-mean-square fluctuation (RMSF, for residue flexibility), radius of gyration (Rg, indicating compactness), solvent-accessible surface area (SASA, reflecting solvent exposure), number of protein–ligand hydrogen bonds (evaluating interaction stability), 2D and 3D free energy landscapes, and MM-PBSA results (including binding free energy and per-residue energy decomposition).

### 2.3. Statistical Analysis

Data are expressed as mean ± SEM. Statistical significance was determined by one-way ANOVA using GraphPad Prism 10.0, with *p* < 0.05 considered statistically significant.

## 3. Results

### 3.1. Chemistry

**Scheme 1:**
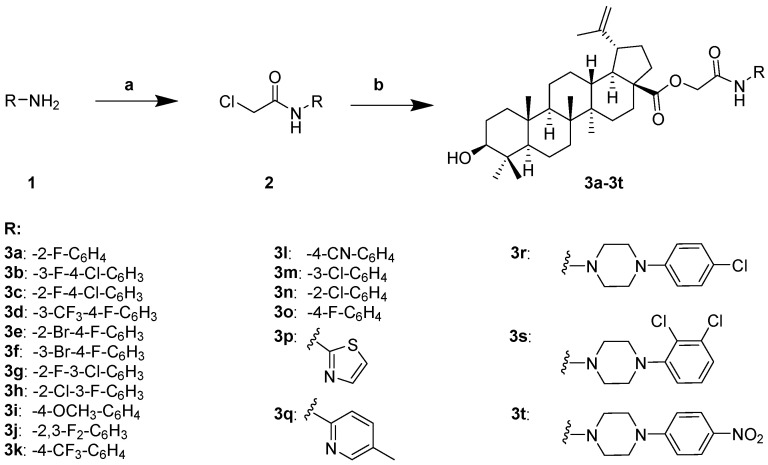
Synthetic route of target compounds **3a**–**3t**. Reagents and conditions: (**a**) ClCH_2_COCl, Et_3_N, CH_2_Cl_2_, −5 °C, 12 h; (**b**) betulinic acid, K_2_CO_3_, CH_3_CN, reflux: 12 h.

The synthetic route for compounds **3a**–**3t** is depicted in Scheme 1. A series of aryl- and heterocyclic-substituted amines (**1**) underwent condensation with chloroacetyl chloride to afford intermediate amides (**2**). Subsequent nucleophilic substitution of these intermediates with the carboxylic acid moiety of betulinic acid (BA) yielded the target derivatives **3a**–**3t** with good to excellent yields. All final compounds were unambiguously characterized by ^1^H NMR, ^13^C NMR, and high-resolution mass spectrometry (HRMS).

### 3.2. Bioassay

#### 3.2.1. Standardized Minimum Inhibitory Concentration (MIC) Assay

Twenty C-28 modified betulinic acid derivatives—categorized as halogenated anilines (**3a**–**3o**), amino polyheterocycles (**3p**–**3q**), and piperazines (**3r**–**3t**)—were evaluated for their in vitro antibacterial activity against six clinically relevant pathogens using the broth microdilution method (Table 1). Betulinic acid (BA), levofloxacin, and tetracycline were included as controls. While the parent compound BA showed no activity against any tested strain (MIC > 250 μg/mL), several derivatives exhibited markedly enhanced potency.

Among them, compounds **3b**, **3d**, and **3e** demonstrated the most potent activity against *Staphylococcus aureus* ATCC 25923, with MIC values of 125, 31.25, and 62.5 μg/mL, respectively. Notably, **3d** and **3e** also effectively inhibited vancomycin-resistant *S. aureus* (VRSA) at 62.5 μg/mL, and **3o** showed selective activity against *Streptococcus pneumoniae* ATCC 49619 (MIC = 125 μg/mL). All active derivatives were specific to Gram-positive bacteria, exhibiting no detectable inhibition against the Gram-negative strains *Escherichia coli* ATCC 25922 and *Pseudomonas aeruginosa* ATCC 27853 within the tested concentration range.

Structure–activity relationship (SAR) analysis revealed that the presence of electron-withdrawing groups on the aniline ring—particularly 4-chloro-3-fluoro (**3b**), 4-fluoro-3-trifluoromethyl (**3d**), 2-bromo-4-fluoro (**3e**), and 4-fluoro (**3o**)—was critical for enhancing antibacterial efficacy. The most potent compound, **3d**, was selected for further mechanistic studies to explore its antibacterial and anti-biofilm properties.

#### 3.2.2. Evaluation of the Antibacterial Efficacy of Compound **3d** by the Oxford Cup Method

**Figure 2:**
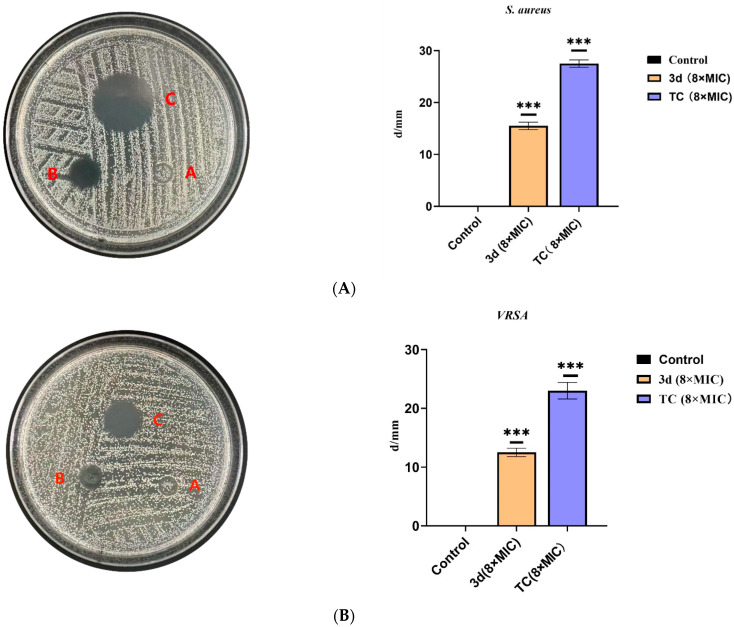
(**A**) Representative images of *S. aureus* growth inhibition. (**B**) Representative images of VRSA growth inhibition: (A) control, (B) compound **3d**, and (C) tetracycline. *** *p* < 0.001 compared with the control group (1% DMSO).

Compound **3d**, as the most potent derivative from the initial screening, was selected for further evaluation of its antibacterial activity against *S. aureus* and VRSA using the Oxford cup method. Tetracycline was used as a positive control due to its broad-spectrum activity and common use in anti-biofilm studies. As shown in Figure 2A, **3d** at 8 × MIC produced a clear inhibition zone of 15.5 mm against *S. aureus* (*p* < 0.001). Against VRSA, an inhibition zone of 11.8 mm (*p* < 0.001) was observed (Figure 2B). These results demonstrate that **3d** exhibits potent and statistically significant antibacterial activity against both drug-sensitive and drug-resistant *S. aureus*, supporting its potential to overcome existing resistance mechanisms and warranting further mechanistic investigation.

#### 3.2.3. Time-Kill Kinetics of Compound **3d** Against *S. aureus* and VRSA

**Figure 3:**
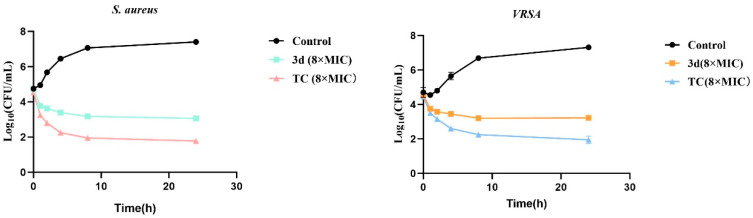
The time-kill curves of compound **3d** against *S. aureus* and VRSA. Control group: 1% DMSO.

To evaluate the bactericidal properties of compound **3d**, time-kill kinetics were performed against *S. aureus* and VRSA over a 24 h period at a concentration of 8 × MIC (Figure 3). Against *S. aureus*, treatment with **3d** resulted in a rapid reduction of 1.31 log_10_ CFU/mL within the first hour and a cumulative reduction of 4.27 log_10_ CFU/mL by the end of the 24 h incubation, with no evidence of bacterial regrowth throughout the experiment. Against VRSA, **3d** also demonstrated notable inhibitory activity, reducing viable counts by 0.80 log_10_ CFU/mL at 1 h and achieving a 4.04 log_10_ CFU/mL reduction at 24 h, similarly without regrowth. These findings are consistent with the Oxford cup assay results and collectively confirm that compound **3d** possesses stable and effective antibacterial activity against both drug-sensitive and drug-resistant *S. aureus* strains. Although the reduction at 24 h exceeded 3 log_10_ for both strains—generally considered bactericidal—the kinetic profile suggests a sustained inhibitory effect rather than rapid killing. This pattern, together with its pronounced anti-biofilm activity, implies that **3d** may act through a mechanism distinct from conventional antibiotics, potentially involving interference with virulence regulatory pathways such as SarA. These characteristics highlight the therapeutic potential of **3d** in combating biofilm-associated and drug-resistant staphylococcal infections.

#### 3.2.4. Effects of Active Target Compounds on Bacterial Biofilm Formation

**Figure 4:**
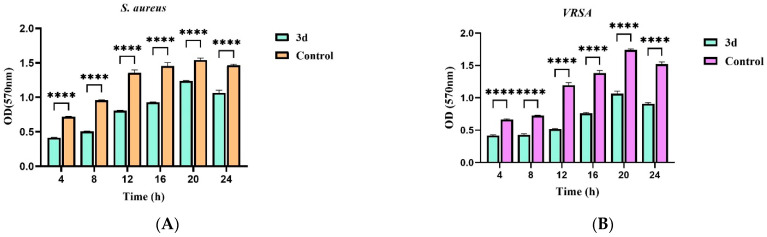
Impact of compound **3d** on biofilm formation. Inhibitory effects of the active compounds on biofilm formation by (**A**) *S. aureus* and (**B**) vancomycin-resistant *S. aureus* (VRSA). **** *p* < 0.0001 compared with the control group (1% DMSO).

Given the potent antibacterial activity of compound **3d** against both drug-sensitive and drug-resistant strains of *S. aureus*, its effect on biofilm formation was further evaluated. As shown in Figure 4, biofilm biomass in the control groups increased progressively over time for both strains. In contrast, treatment with **3d** at 8 × MIC significantly inhibited biofilm formation at all time points assessed (4, 20, and 24 h; *p* < 0.0001). The sustained suppression of biofilm accumulation suggests that **3d** may interfere with key stages of biofilm development, including initial attachment or extracellular matrix production. These findings indicate that compound **3d** not only exhibits direct antibacterial activity but also effectively disrupts the biofilm formation process, possibly through targeting regulatory mechanisms involved in biofilm maturation.

#### 3.2.5. Molecular Docking Analysis

**Figure 5:**
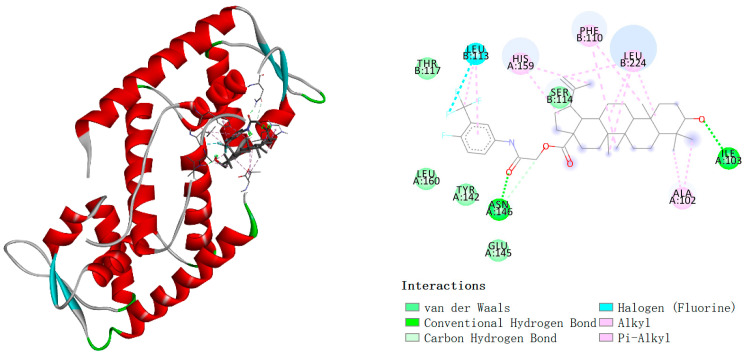
Docking pose of compound **3d** within the SarA (2FRH) binding pocket.

The staphylococcal accessory regulator A (SarA) is a key transcriptional regulator of virulence factors in *S. aureus* and has been established as indispensable for biofilm formation [31]. Given our prior experimental evidence that compound **3d** significantly inhibits biofilm formation in *S. aureus*, we postulated SarA as its potential molecular target. To elucidate the underlying structural mechanism, molecular docking was performed, which also provides a theoretical basis for identifying targets of analogous compounds. Molecular docking was performed using AutoDock Vina 2.1.6. Upon completion of the docking process, the conformation exhibiting the lowest binding energy and the highest frequency of recurrent binding poses was selected as the output. The results were subsequently imported into PyMol 3.1 and Discovery Studio 2019 for visualization and analysis. Analysis of the three-dimensional docking model revealed that the ligand forms two hydrogen bonds with the ILE 103 and ASN 146 amino acid residues of the protein, suggesting that these residues serve as key binding sites (Figure 5). In addition, halogen interactions, alkyl interactions, and pi–alkyl interactions were observed between the ligand and the protein, which further contributed to the binding affinity. The calculated binding energy for this docking complex was −6.89 kcal/mol, indicating a strong interaction between the ligand and the protein. Furthermore, the surface model illustrated that the ligand is embedded within a cavity on the protein surface, exhibiting structural complementarity with the binding site. This spatial accommodation is conducive to stable binding.

#### 3.2.6. Molecular Dynamics Simulation

**Figure 6:**
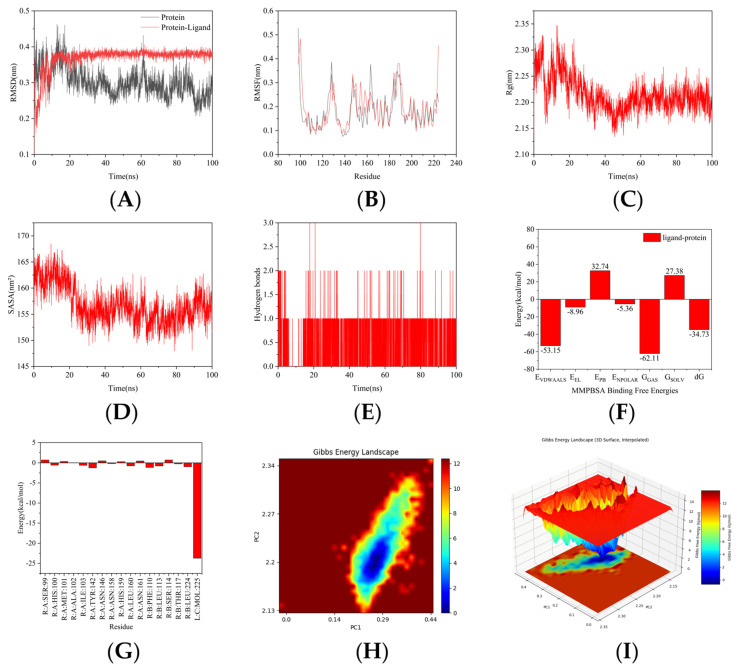
Binding stability and conformational dynamics of compound **3d** from molecular dynamics simulations: (**A**) Root-mean-square deviation (RMSD) of the protein backbone. (**B**) Residue-wise root-mean-square fluctuation (RMSF). (**C**) Radius of gyration (Rg) of the protein. (**D**) Solvent-accessible surface area (SASA). (**E**) Number of protein–ligand hydrogen bonds over time. (**F**) Binding free energy calculated by the MM-PBSA method. (**G**) Binding energy decomposition of key residues. (**H**,**I**) Two-dimensional and three-dimensional conformational free energy landscapes.

Based on the molecular docking results described above, a 100 ns molecular dynamics (MD) simulation was further performed using GROMACS 2025 for the protein (SarA, PDB: 2FRH)–ligand (**3d**) complex. The specific results are summarized as follows: After 10 ns, the RMSD values plateaued, indicating that the protein reached a stable state upon ligand binding. The absence of significant movement of the ligand within the binding site suggests a stable binding mode (Figure 6A). Most residues exhibited low RMSF values, reflecting the structural stability of the protein. Higher fluctuations observed in the *N*-terminal region may correspond to a flexible loop or terminal dynamics (Figure 6B). After 10 ns, the radius of gyration (Rg) decreased and stabilized, implying that the protein adopted a more compact conformation in the presence of the ligand (Figure 6C). A decrease in the solvent accessible surface area (SASA) after 10 ns further indicated tighter packing of the protein around the ligand, corroborating the stability of the complex (Figure 6D). The number of hydrogen bonds remained stable after 10 ns, demonstrating consistent and favorable interactions between the protein and the ligand throughout the simulation (Figure 6E). The calculated binding free energy was −34.73 kcal/mol, suggesting spontaneous and thermodynamically stable binding of the ligand to the protein. This provides an energetic rationale for further investigation into the biological functions of this ligand–protein complex (Figure 6F). Residues such as TYR-42, PHE-110, and LEU-224 contributed favorably to binding, as indicated by their negative energy values in the residue-wise decomposition analysis, highlighting their key roles in stabilizing the protein–ligand interactions (Figure 6G). The free energy landscape revealed deep blue basins corresponding to highly populated, low-energy conformational states of the bound complex, while yellow/red regions indicated energy barriers or transition states between conformational clusters, reflecting the dynamic bottlenecks during conformational sampling (Figure 6H,I). Collectively, these results demonstrate that compound **3d** exhibits strong binding affinity to SarA, supported by both structural stability throughout the simulation and favorable binding energetics.

## 4. Discussion

The results of this study demonstrate that structural modification of BA at the C-28 position with halogenated aniline moieties significantly enhances its antibacterial and anti-biofilm effects against *S. aureus*, including vancomycin-resistant strains (VRSA). Our synthetic strategy focused on the direct amidation of the C-28 carboxyl group of BA with substituted anilines via a chloroacetyl chloride linker, leading to a focused library of 20 derivatives (**3a**–**3t**). This approach prioritized synthetic accessibility and systematically explored the electronic effects of substituents, particularly electron-withdrawing groups, on enhancing activity against Gram-positive bacteria. This finding aligns with and extends previous reports on the structure–activity relationships of BA derivatives.

A comparison with alternative strategies in the field provides a clearer context for the characteristics and value of our work. For instance, Spivak et al. demonstrated that introducing basic amine and guanidine groups at C-28 markedly improved activity against Gram-positive bacteria, including *S. aureus* [26]. In contrast, Batool et al. adopted a different approach: first converting betulinic acid into a hydrazide ligand, followed by complexation with various metals (Fe, Cu, Zn, Sn, and Sb) to form organometallic complexes [32]. Their method leveraged the potential of metal coordination to alter bioactivity; the resulting complexes exhibited activity against both Gram-positive and Gram-negative strains, demonstrating a broader antibacterial spectrum than that observed for our purely organic derivatives. This suggests that metallation may be a viable strategy to overcome the intrinsic resistance of triterpenoid scaffolds against Gram-negative bacteria. Tsepaeva et al. employed yet another distinct strategy by conjugating BA or betulin with a triphenylphosphonium (TPP) cation via linkers of varying lengths [33]. TPP is a well-known mitochondria-targeting moiety; this design aimed to enhance intracellular delivery and potentially disrupt energy metabolism. Their TPP conjugates showed very low MIC values (2–3 µM) against staphylococci and provided a mechanistic perspective involving mitochondrial dysfunction. Furthermore, Schuhly et al. [34] reported naturally occurring BA esters with aromatic acids at the C-3 or C-28 positions, which exhibited MICs of 64 µg/mL against *Staphylococcus epidermidis*—an activity range comparable to that of some of our derivatives. Their work indicates that esterification at positions other than C-28, particularly with aromatic acids, can also confer significant antibacterial activity, a principle consistent with our strategy of using stable amide linkers.

Our most potent compound, **3d** (MIC = 31.25 µg/mL against *S. aureus*), may exhibit somewhat lower direct antibacterial potency than some of the aforementioned extreme cases (e.g., metal complexes or TPP conjugates). However, it incorporates a unique halogenated aryl amide pharmacophore strategy, which may offer advantages in terms of physicochemical properties and potential anti-biofilm specificity. By systematically exploring electron-withdrawing halogenated anilines, our work revealed a clear positive correlation between the electron deficiency of the aryl ring and enhanced anti-staphylococcal activity, as evidenced by the superior performance of **3d** (bearing a –CF_3_ group) and **3e** (bearing a –Br group).

Time-kill kinetics indicated that the lead compound **3d** reduced viable counts by >4 log_10_ CFU/mL over 24 h against both susceptible and VRSA strains, meeting a conventional threshold for bactericidal reduction, although the kinetics suggested a predominantly bacteriostatic profile at the tested concentration. This profile is reminiscent of some pentacyclic triterpenoid derivatives whose primary strength lies in inhibiting virulence rather than causing rapid killing [29]. Notably, the pronounced and consistent suppression of biofilm formation by **3d** across time points represents a significant finding. Its anti-biofilm efficacy compares favorably with other recently described natural product derivatives targeting staphylococcal biofilms, such as certain ursolic acid conjugates that act via membrane disruption [28], and hints at a potentially distinct mechanism. Our integrated computational studies propose a novel mechanism involving interference with the transcriptional regulator SarA, a key virulence controller less commonly targeted by traditional triterpenoid-based antibacterials. This virulence-targeting mode could offer a strategic advantage by potentially reducing the selection pressure for conventional resistance development.

Nevertheless, the lack of activity of our compound series against Gram-negative bacteria (*E. coli*, *P. aeruginosa*) highlights a common limitation of many triterpenoid derivatives, likely attributable to the impermeable outer membrane and/or efficient efflux systems in Gram-negative pathogens [22]. This contrasts with some broad-spectrum synthetic antibiotics but aligns with the typical spectrum of many BA derivatives. In this regard, the metallation strategy employed by Batool et al. offers a useful insight for overcoming this limitation.

Looking forward, this study points to several promising directions. First, comprehensive in vitro cytotoxicity profiling against mammalian cell lines and hemolytic assays are essential next steps to establish a therapeutic window for lead compounds such as **3d**. Second, given its anti-biofilm activity and the proposed SarA-targeting mechanism, combination therapy studies with conventional antibiotics (e.g., vancomycin, daptomycin) are warranted to explore potential synergy in eradicating established biofilms or treating persistent infections. Third, the proposed mechanism of SarA inhibition requires further genetic validation—for example, using sarA-deletion mutant strains to confirm the specificity of the anti-biofilm effect or conducting transcriptomic analyses to assess downstream changes in virulence gene expression. Fourth, structural optimization could focus on improving potency and broadening the spectrum. For instance, the rational hybridization of the potent halogenated aniline pharmacophore of **3d** with other promising strategies—such as the basic amine groups from Spivak’s work, the metal coordination approach of Batool et al., or the targeted delivery modules from Tsepaeva et al.—may yield next-generation derivatives with enhanced activity and improved properties. Finally, advancing the most promising lead into in vivo efficacy models of biofilm-associated infection would represent a crucial milestone in preclinical development.

Regarding the potential applicability of these findings, it is important to address the translational prospects of compound **3d**. A Chinese invention patent (No. 202610032775.X) has been filed for the compounds and their antibacterial activity reported in this study, representing an initial step toward protecting the intellectual property associated with this novel class of derivatives. However, it should be emphasized that patent filing is only the first stage in a lengthy drug development process. Prior to any potential commercial development, comprehensive preclinical evaluations—including in vivo efficacy studies, pharmacokinetic profiling, and detailed toxicity assessments—will be required to establish a favorable therapeutic index and ensure safety.

Another key consideration is the strain specificity of the observed antibacterial activity. The present study focused on standard laboratory strains and a clinical isolate of VRSA. While compound **3d** demonstrated potent activity against these strains, further testing against a broader panel of *S. aureus* clinical isolates derived from diverse infection sites (e.g., bloodstream, wound, respiratory tract) and various geographical origins would be necessary to establish the generalizability of its efficacy. Such studies would help determine whether the activity of **3d** is conserved across genetically distinct *S. aureus* lineages or is influenced by strain-specific factors such as resistance profiles or virulence gene expression patterns.

From a commercial development perspective, several critical steps remain before this compound class can advance toward clinical application. These include (i) optimization of the lead compound to improve potency and pharmacokinetic properties; (ii) systematic evaluation of synergistic effects with existing antibiotics; (iii) development of suitable formulations; and (iv) rigorous assessment of safety and efficacy in appropriate animal models of infection. The anti-biofilm properties of **3d**, in particular, position it as a potential candidate for combination therapies aimed at eradicating biofilm-associated persistent infections, an area of high unmet medical need.

In summary, the C-28 direct amidation functionalization strategy described herein, particularly using electron-deficient aryl amides, offers a fruitful avenue for developing dual-action antibacterial and anti-biofilm agents. Compound **3d** serves as a valuable lead candidate, demonstrating potent activity against drug-resistant *staphylococci* and representing a starting point for the development of a novel class of anti-virulence agents targeting regulatory proteins such as SarA. Different structural modification strategies—metallation, targeted conjugation, esterification, etc.—each possess distinct advantages. Future work could integrate their strengths through cross-disciplinary fusion to promote the discovery of more breakthrough anti-resistance agents.

## 5. Conclusions

In conclusion, a series of C-28 modified betulinic acid derivatives was successfully synthesized and evaluated. Among them, compound **3d** exhibited potent and broad-spectrum antibacterial activity against both drug-sensitive and drug-resistant *S. aureus*, as demonstrated by multiple assays, including MIC determination, Oxford cup, time-kill kinetics, and biofilm inhibition. Molecular docking suggested that the anti-biofilm effect may be mediated through inhibition of the SarA protein. This mechanism was further supported by molecular dynamics simulations, which confirmed a stable binding interaction between **3d** and SarA. Overall, this study not only validates the C-28 modification strategy as a viable approach to enhancing antibacterial activity but also highlights **3d** as a promising lead compound worthy of further development against biofilm-associated resistant infections. Comprehensive cytotoxicity assessments, including mammalian cell toxicity and hemolysis studies, are to be integrated into the subsequent stages of lead optimization and preclinical development. With its potent activity against drug-resistant *S. aureus* and its ability to inhibit biofilm formation, compound **3d** warrants further preclinical development as a candidate for treating persistent staphylococcal infections.

## Figures and Tables

**Table 1:** MIC values of BA derivatives against six bacterial pathogens (μg/mL).

Entry	*S. aureus*	VRSA	*S. pneumoniae*	*S. epidermidis*	*E. coli*	*P. aeruginosa*
	ATCC25923		ATCC49619	ATCC12228	ATCC25922	ATCC27853
**3a**	>250	>250	>250	>250	>250	>250
**3b**	125	>250	>250	>250	>250	>250
**3c**	>250	>250	>250	>250	>250	>250
**3d**	31.25	62.25	>250	>250	>250	>250
**3e**	62.25	62.25	>250	>250	>250	>250
**3f**	>250	>250	>250	>250	>250	>250
**3g**	>250	>250	>250	>250	>250	>250
**3h**	>250	>250	>250	>250	>250	>250
**3i**	>250	>250	>250	>250	>250	>250
**3j**	>250	>250	>250	>250	>250	>250
**3k**	>250	>250	>250	>250	>250	>250
**3l**	>250	>250	>250	>250	>250	>250
**3m**	>250	>250	>250	>250	>250	>250
**3n**	>250	>250	>250	>250	>250	>250
**3o**	>250	>250	125	>250	>250	>250
**3p**	>250	>250	>250	>250	>250	>250
**3q**	>250	>250	>250	>250	>250	>250
**3r**	>250	>250	>250	>250	>250	>250
**3s**	>250	>250	>250	>250	>250	>250
**3t**	>250	>250	>250	>250	>250	>250
BA	>250	>250	>250	>250	>250	>250
Levofloxacin	0.49	3.91	31.25	0.49	0.49	0.49
Tetracycline	0.49	31.25	62.25	15.625	0.49	15.625

## Data Availability

The original contributions presented in this study are included in the article/Supplementary Materials. Further inquiries can be directed to the corresponding authors.

## Supplementary Materials

The following supporting information can be downloaded at: https://www.mdpi.com/article/10.3390/microorganisms14030574/s1, Figure S1: (1-1): ^1^H NMR spectrum of compound **3a**; (1-2): ^13^C NMR spectrum of compound **3a**; (1-3): HRMS spectrum of compound **3a**; Figure S2: (2-1) ^1^H NMR spectrum of compound **3b**; (2-2): ^13^C NMR spectrum of compound **3b**; (2-3): HRMS spectrum of compound **3b**; Figure S3: (3-1): ^1^H NMR spectrum of compound **3c**; (3-2): ^13^C NMR spectrum of compound **3c**; (3-3): HRMS spectrum of compound **3c**; Figure S4: (4-1): ^1^H NMR spectrum of compound **3d**; (4-2): ^13^C NMR spectrum of compound **3d**; (4-3): HRMS spectrum of compound **3d**; Figure S5: (5-1): ^1^H NMR spectrum of compound **3e**; (5-2): ^13^C NMR spectrum of compound **3e**; (5-3): HRMS spectrum of compound **3e**; Figure S6: (6-1): ^1^H NMR spectrum of compound **3f**; (6-2): ^13^C NMR spectrum of compound **3f**; (6-3): HRMS spectrum of compound **3f**; Figure S7: (7-1): ^1^H NMR spectrum of compound **3g**; (7-2): ^13^C NMR spectrum of compound **3g**; (7-3): HRMS spectrum of compound **3g**; Figure S8: (8-1): ^1^H NMR spectrum of compound **3h**; (8-2): ^13^C NMR spectrum of compound **3h**; (8-3): HRMS spectrum of compound **3h;** Figure S9: (9-1): ^1^H NMR spectrum of compound **3i**; (9-2): ^13^C NMR spectrum of compound **3i**; (9-3): HRMS spectrum of compound **3i;** Figure S10: (10-1): ^1^H NMR spectrum of compound **3j**; (10-2): ^13^C NMR spectrum of compound **3j**; (10-3): HRMS spectrum of compound **3j**; Figure S11: (11-1): ^1^H NMR spectrum of compound **3k**; (11-2): ^13^C NMR spectrum of compound **3k**; (11-3): HRMS spectrum of compound **3k**; Figure S12: (12-1): ^1^H NMR spectrum of compound **3l**; (12-2): ^13^C NMR spectrum of compound **3l**; (12-3): HRMS spectrum of compound **3l**; Figure S13: (13-1): ^1^H NMR spectrum of compound **3m;** (13-2): ^13^C NMR spectrum of compound **3m**; (13-3): HRMS spectrum of compound **3m;** Figure S14: (14-1): ^1^H NMR spectrum of compound **3n**; (14-2): ^13^C NMR spectrum of compound **3n**; (14-3): HRMS spectrum of compound **3n**; Figure S15: (15-1): ^1^H NMR spectrum of compound **3o**; (15-2): ^13^C NMR spectrum of compound **3o**; (15-3): HRMS spectrum of compound **3o**; Figure S16: (16-1): ^1^H NMR spectrum of compound **3p**; (16-2): ^13^C NMR spectrum of compound **3p**; (16-3): HRMS spectrum of compound **3p**; Figure S17: (17-1): ^1^H NMR spectrum of compound **3q**; (17-2): ^13^C NMR spectrum of compound **3q**; (17-3): HRMS spectrum of compound **3q**; Figure S18: (18-1): ^1^H NMR spectrum of compound **3r;** (18-2): ^13^C NMR spectrum of compound **3r**; (18-3): HRMS spectrum of compound **3r**; Figure S19: (19-1): ^1^H NMR spectrum of compound **3s**; (19-2): ^13^C NMR spectrum of compound **3s**; (19-3): HRMS spectrum of compound **3s**; Figure S20: (20-1): ^1^H NMR spectrum of compound **3t**; (20-2): ^13^C NMR spectrum of compound **3t**; (20-3): HRMS spectrum of compound **3t**; Figure S21: Effect of the solvent DMSO on the biofilms of two *S. aureus* strains.
