# Supplementary material for: Design, Synthesis, and Anti-Biofilm Activity of C-28 Modified Betulinic Acid Derivatives Targeting SarA in Drug-Resistant Staphylococcus aureus
Source: Microorganisms. 2026 Mar 3;14(3):574. doi: 10.3390/microorganisms14030574 (PMC13029524; doi:10.3390/microorganisms14030574)
Supplement: Supplementary file 1 [file microorganisms-14-00574-s001.zip › microorganisms-4163834-supplementary.pdf]

Design, Synthesis, and Anti-Biofilm Activity of C-28 Modified Betulinic Acid Derivatives Targeting SarA in Drug-Resistant *Staphylococcus aureus*

Dongshun Jia [1, 2, †], Junchao Zhang [1, 2, †], Xuejin Zhang [1, †], Peng Gao [3], Hongyu Zhan [3], Zihan Dong [1], Hao Li [1], Fanhao Meng [2], Nan Cai [1, *], Dajun Zhang [1, 2, *]

[†]: These authors contributed equally to this work.
*: Corresponding authors.

1. School of Pharmacy, Shenyang Medical College, Shenyang, China.
2. School of Pharmacy, China Medical University, Shenyang, China.
3. Faculty of Dentistry, The University of Hong Kong, Hong Kong.

Page 2-38: ^{1}H NMR, ^{13}C NMR, and HRMS spectra and formulas of target compounds **3a** to **3t**.
Page 38: Effect of the solvent DMSO on biofilms.

To whom Correspondence should be addressed. E-mail address: ladycainan@126.com (Nan Cai), zhangdajun2008@126.com (Dajun Zhang)

1.1 ^{1}H NMR, ^{13}C NMR and HRMS spectra target compounds 3a to 3t.

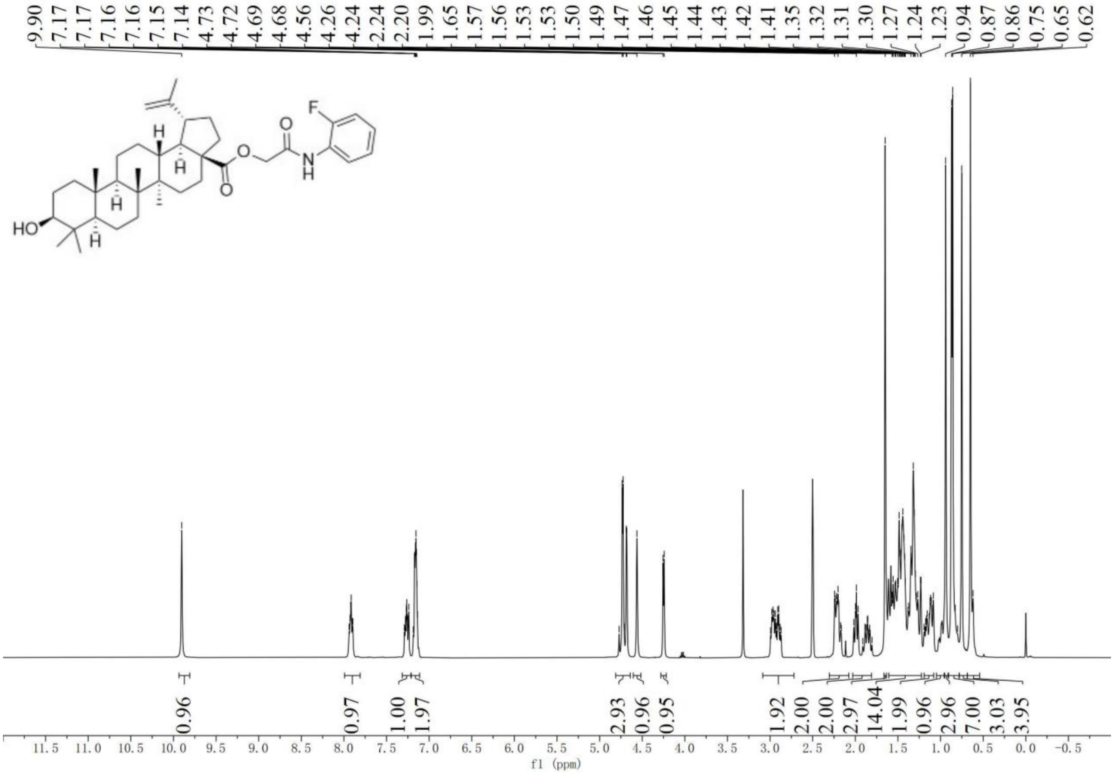

Figure S1-1 ^{1}H NMR spectrum of compound **3a**.

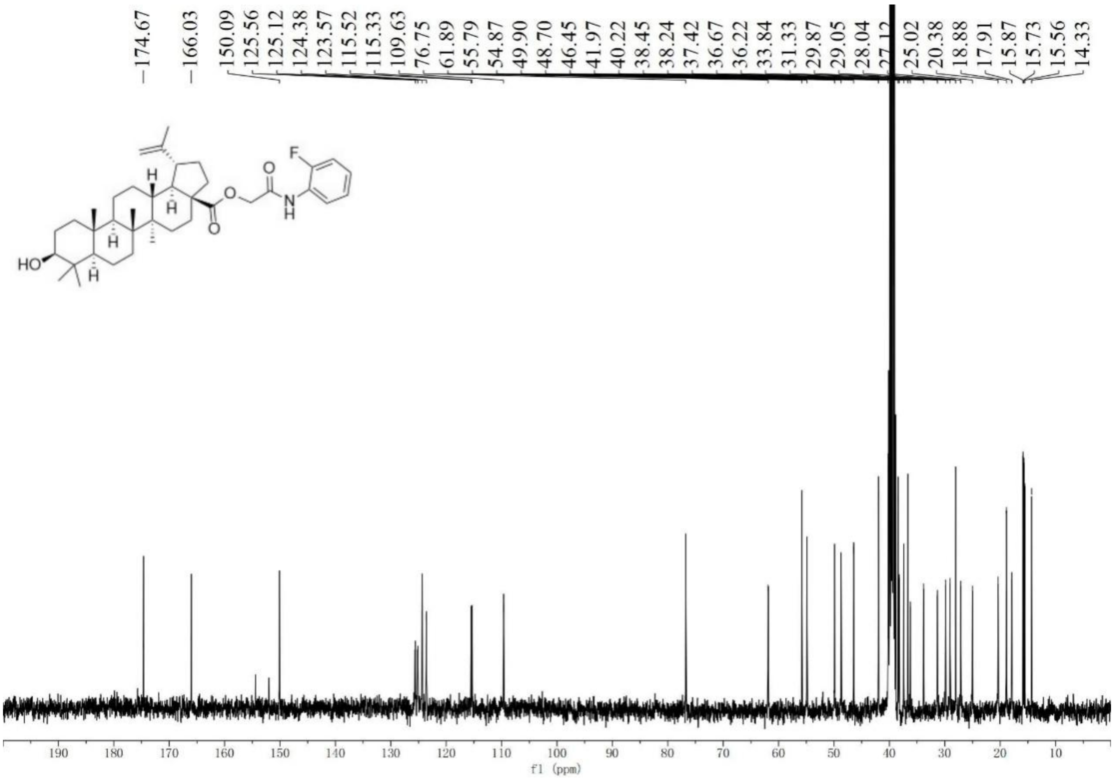

Figure S1-2 ^{13}C NMR spectrum of compound **3a**.

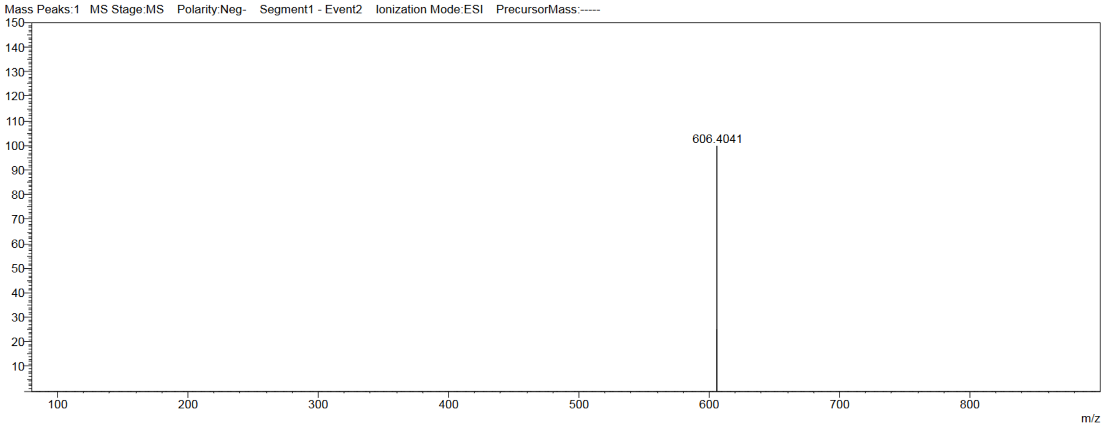

Figure S1-3 HRMS spectrum of compound **3a**.

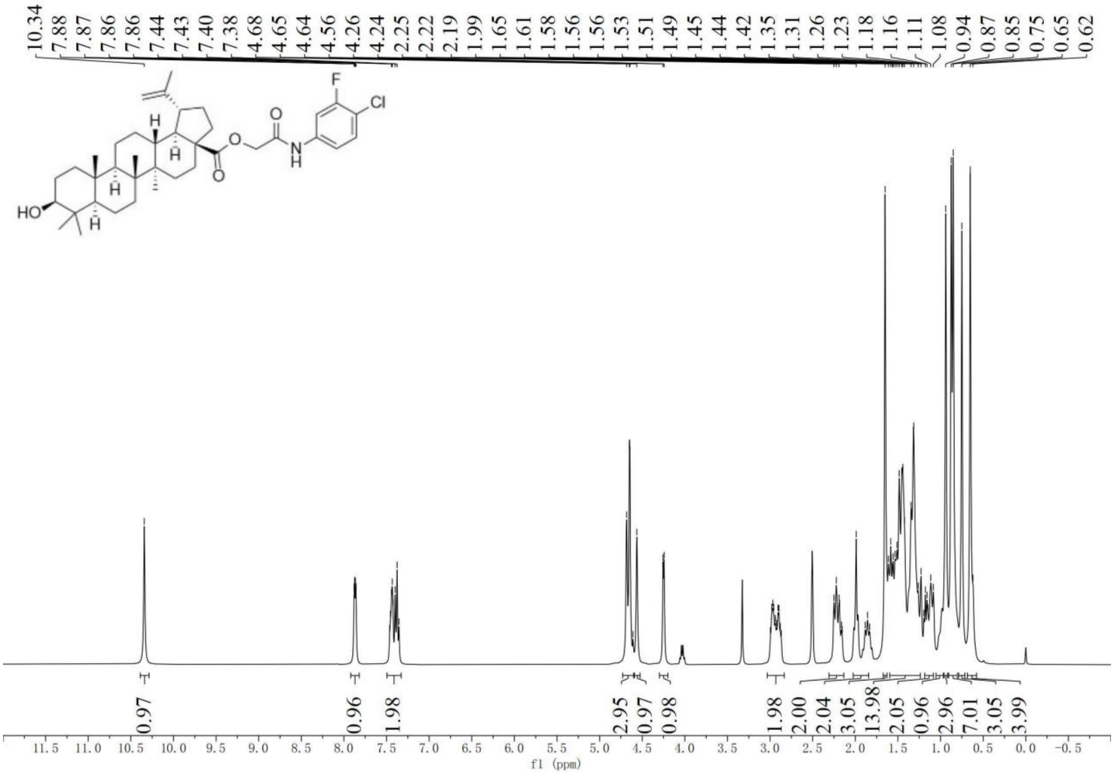

Figure S2-1 ^{1}H NMR spectrum of compound **3b**.

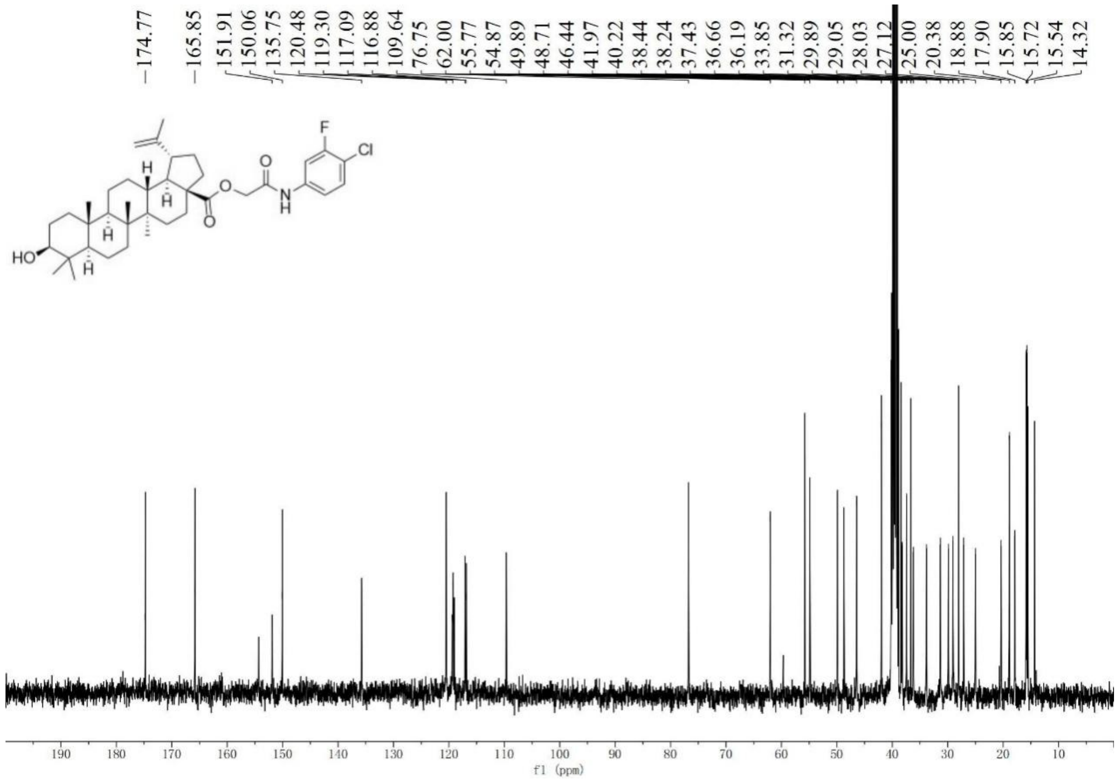

Figure S2-2 ^{13}C NMR spectrum of compound **3b**.

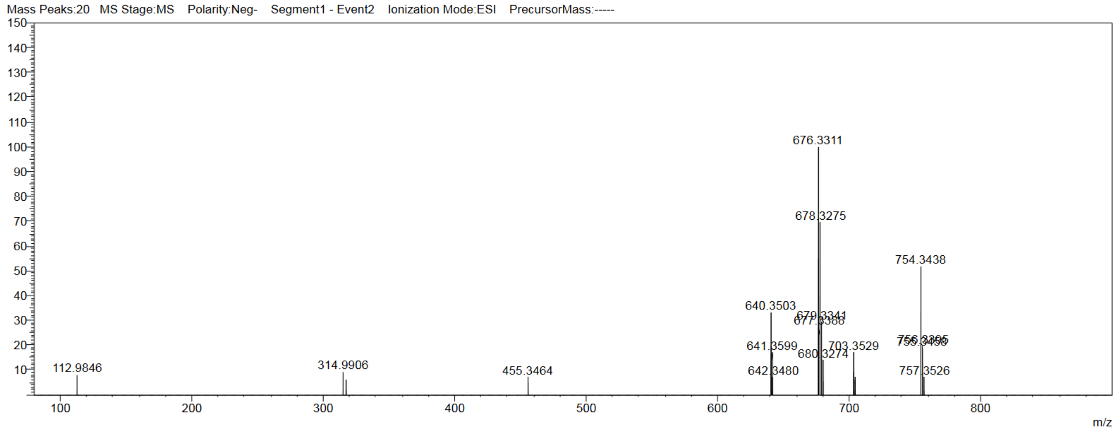

Figure S2-3 HRMS spectrum of compound **3b**.

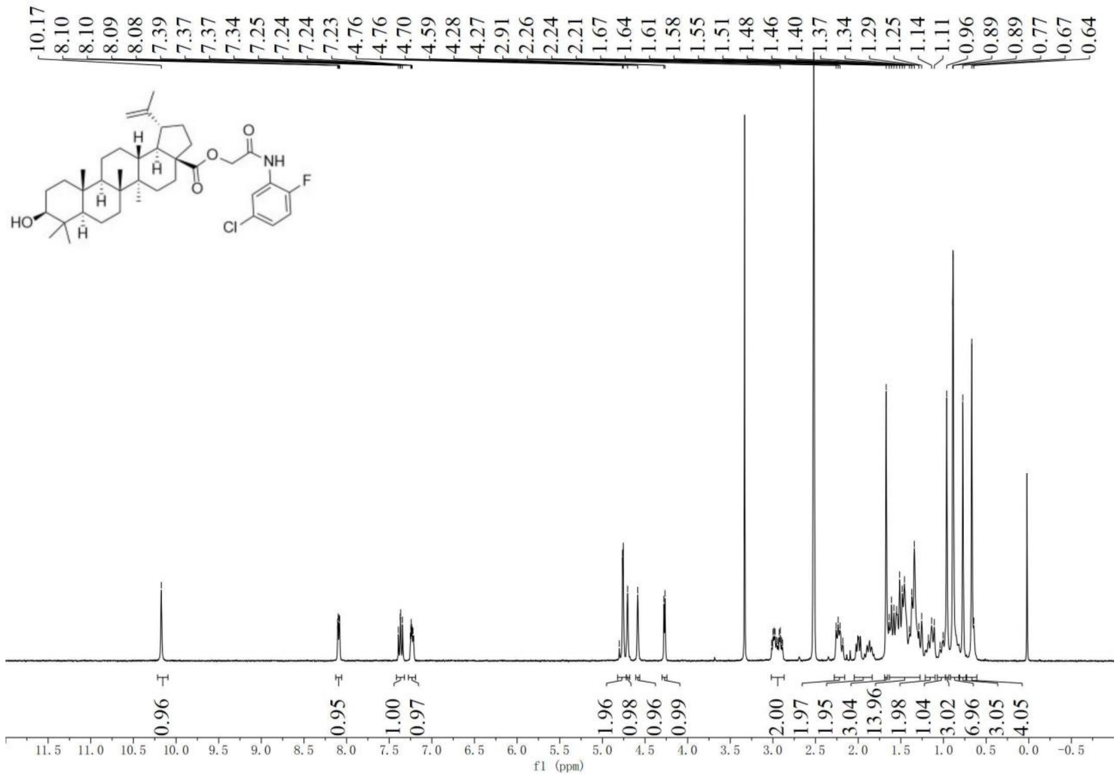

Figure S3-1 ^{1}H NMR spectrum of compound **3c**.

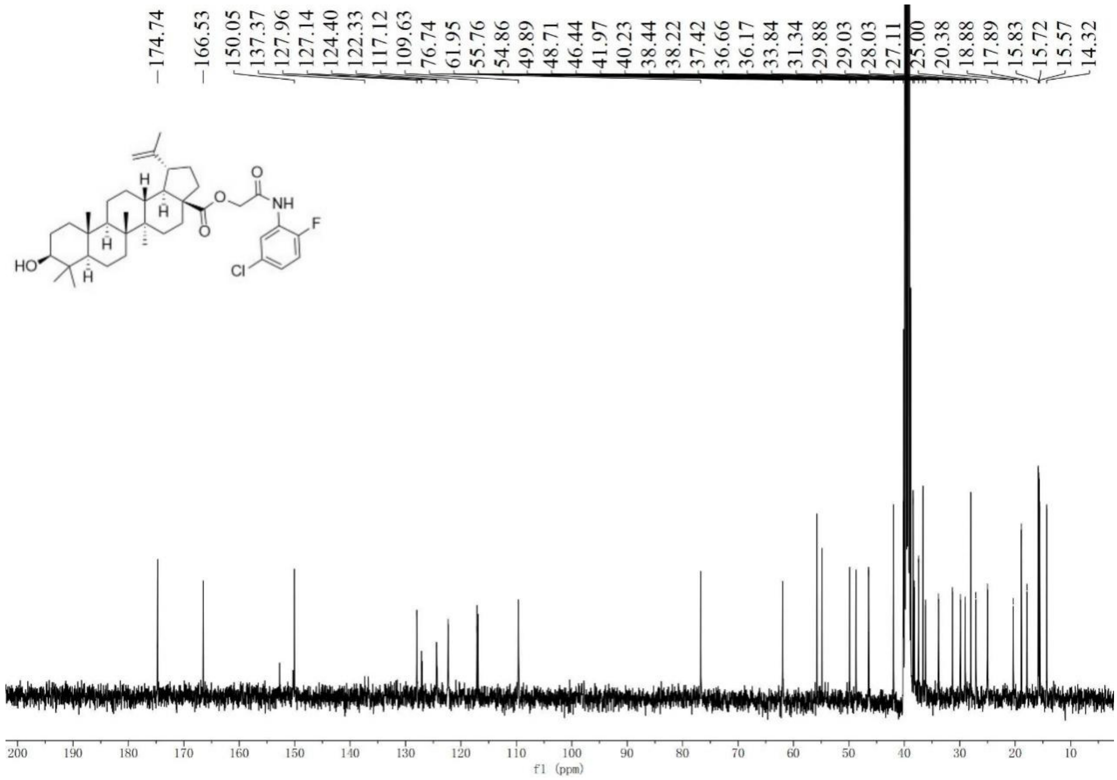

Figure S3-2 ^{13}C NMR spectrum of compound **3c**.

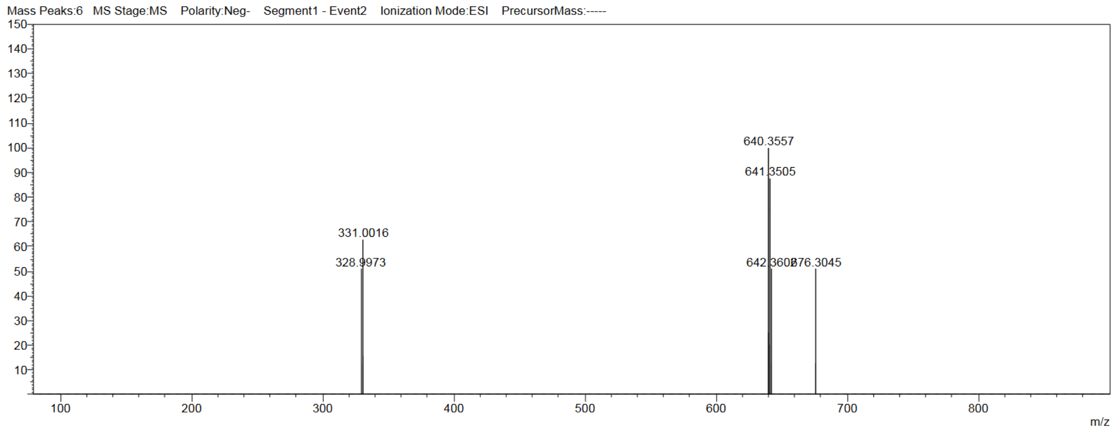

Figure S3-3 HRMS spectrum of compound **3c**.

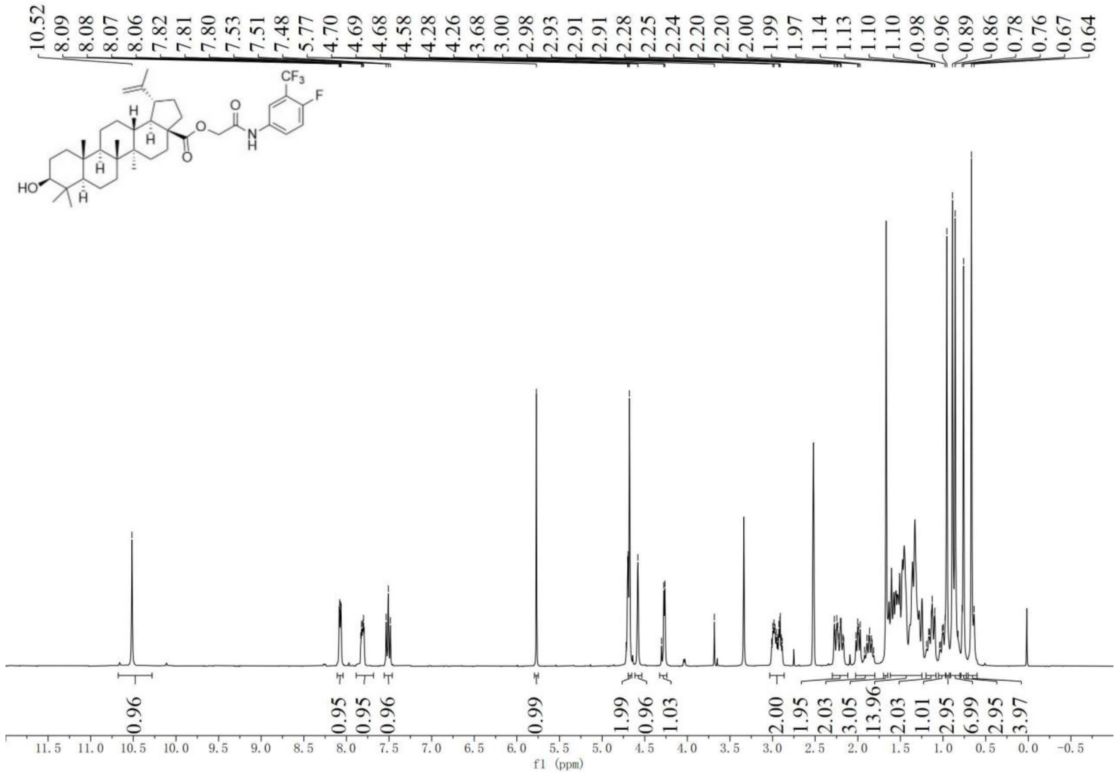

Figure S4-1 ^{1}H NMR spectrum of compound **3d**.

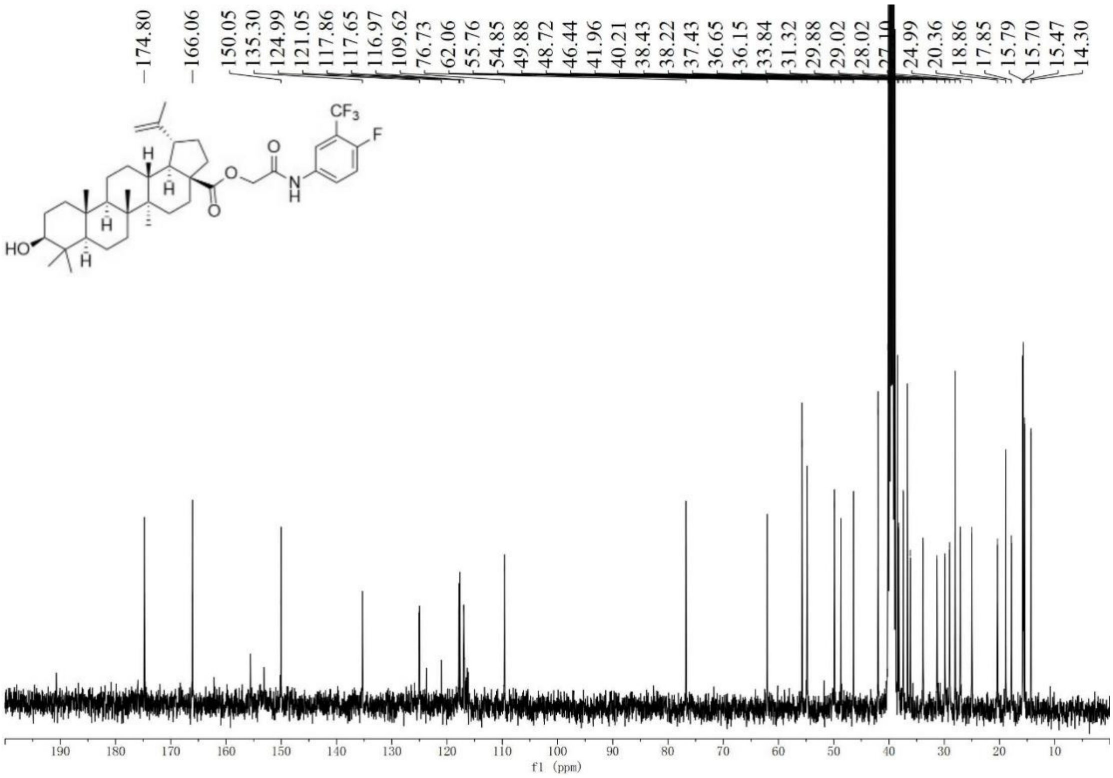

Figure S4-2 ^{13}C NMR spectrum of compound **3d**.

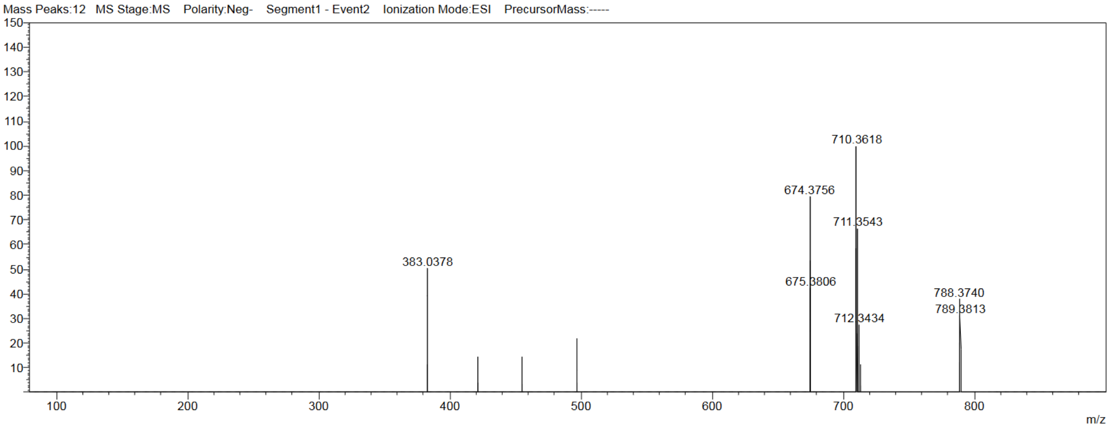

Figure S4-3 HRMS spectrum of compound **3d**.

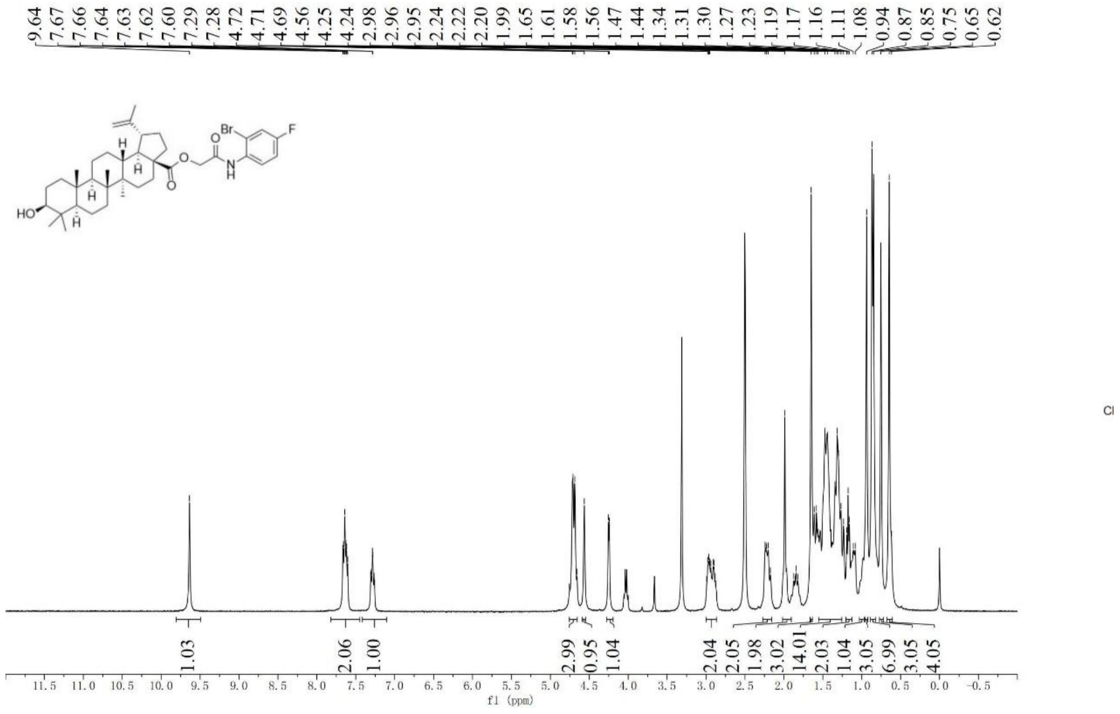

Figure S5-1 ^{1}H NMR spectrum of compound **3e**.

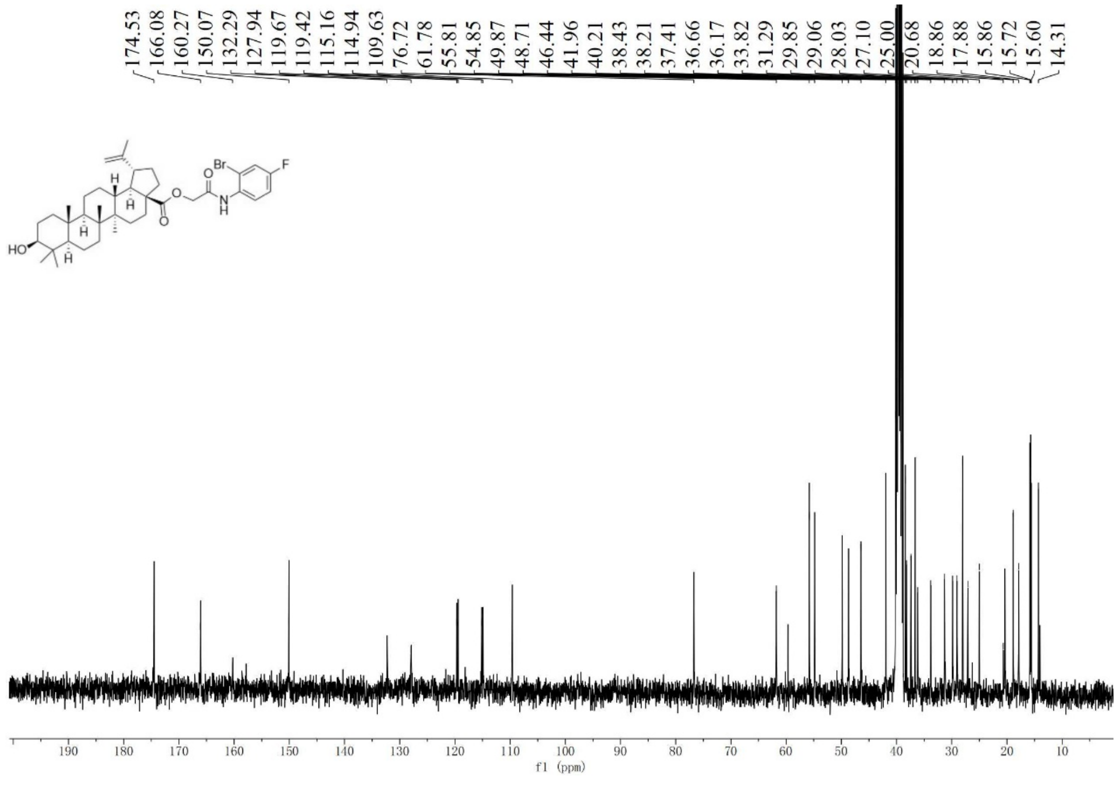

Figure S5-2 ^{13}C NMR spectrum of compound **3e**.

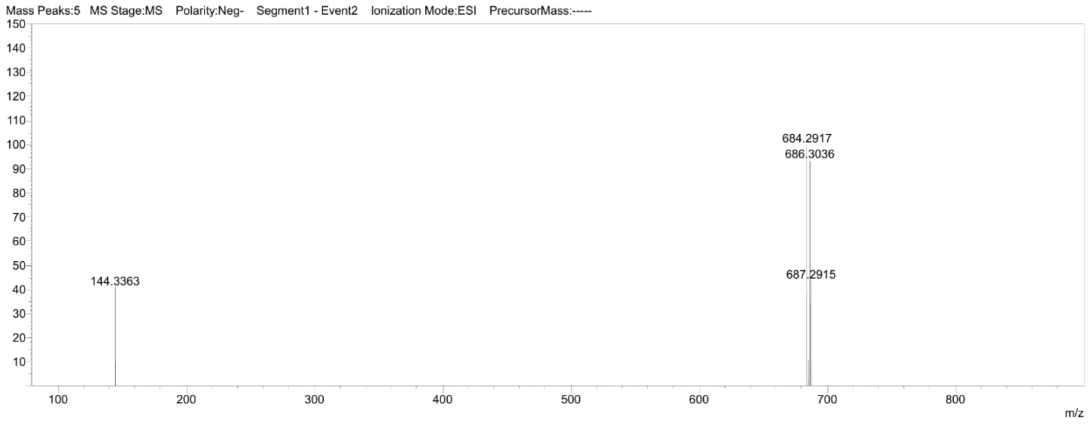

Figure S5-3 HRMS spectrum of compound **3e**.

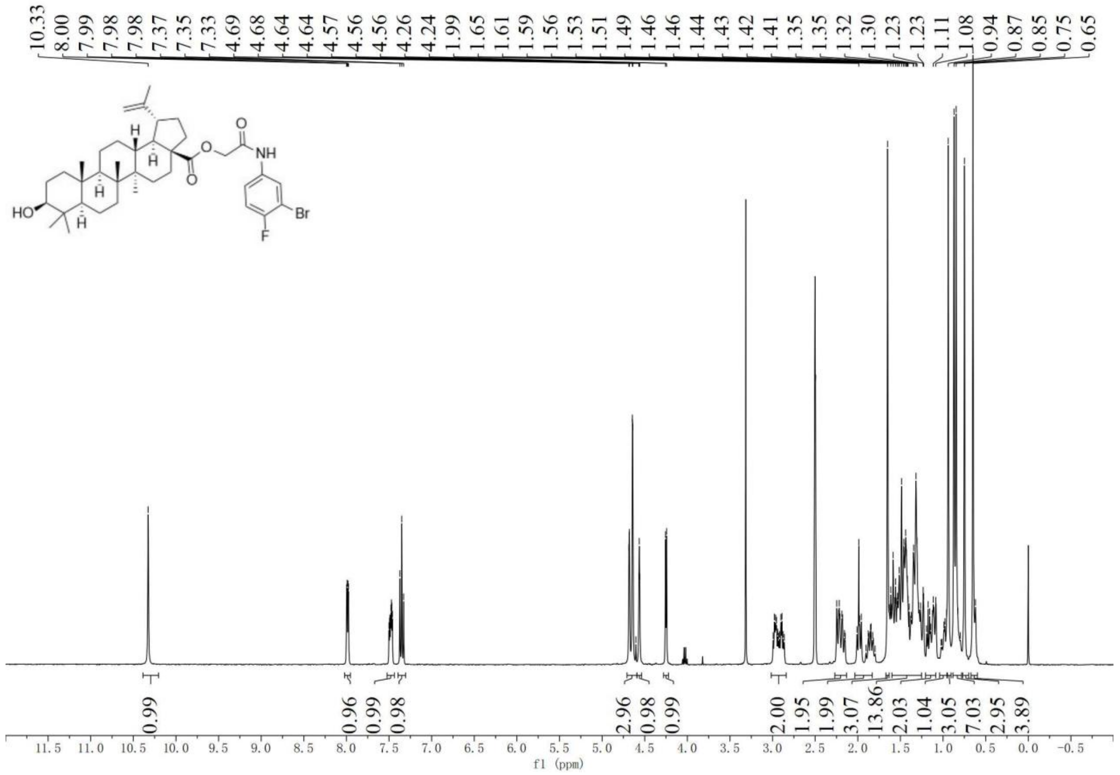

Figure S6-1 ^{1}H NMR spectrum of compound **3f**.

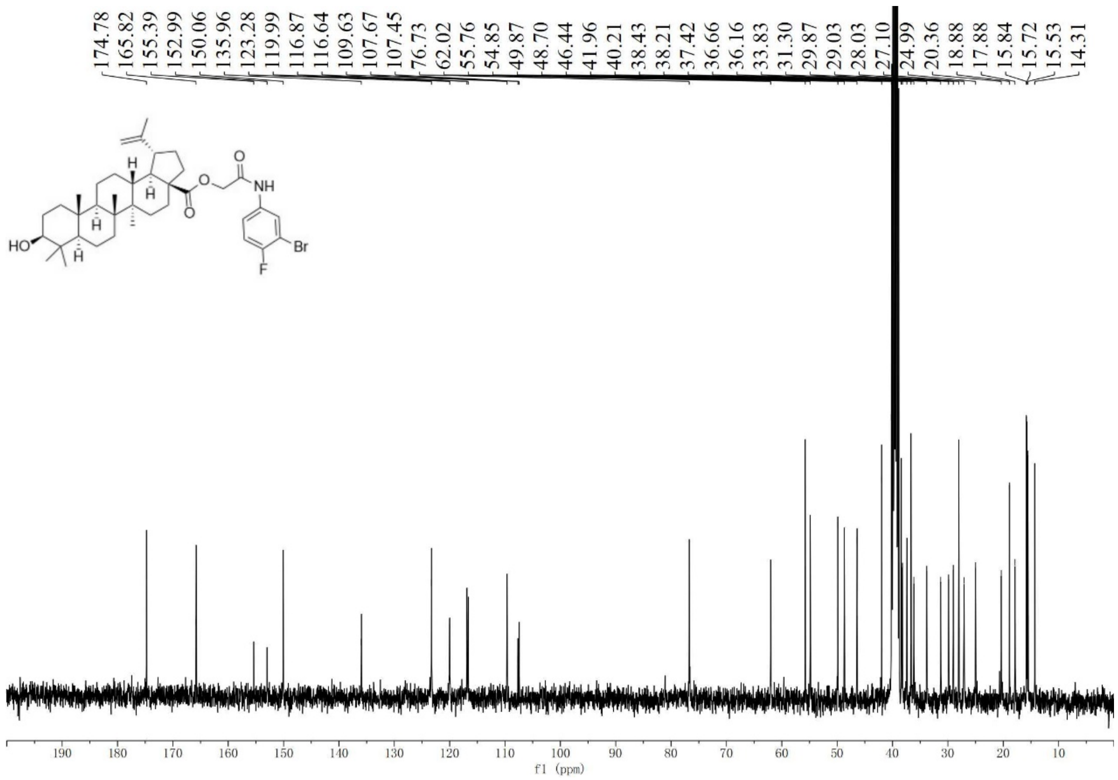

Figure S6-2 ^{13}C NMR spectrum of compound **3f**.

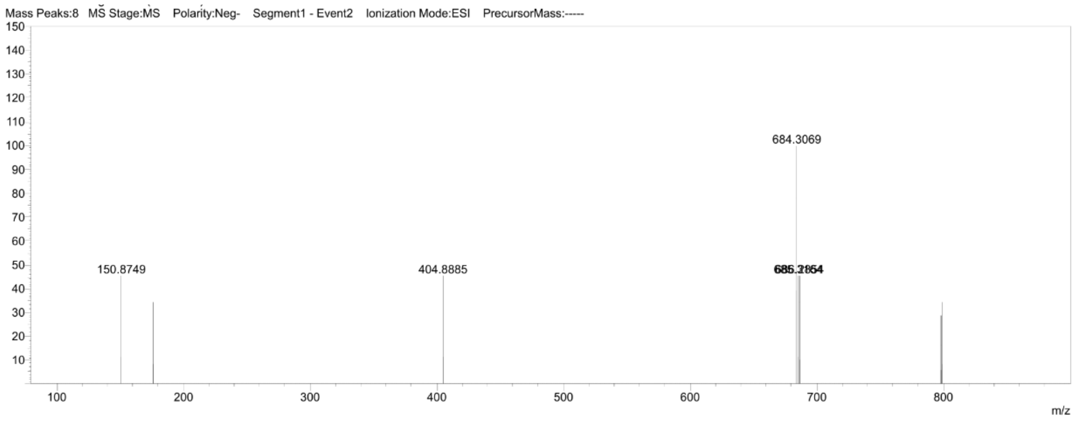

Figure S6-3 HRMS spectrum of compound **3f**.

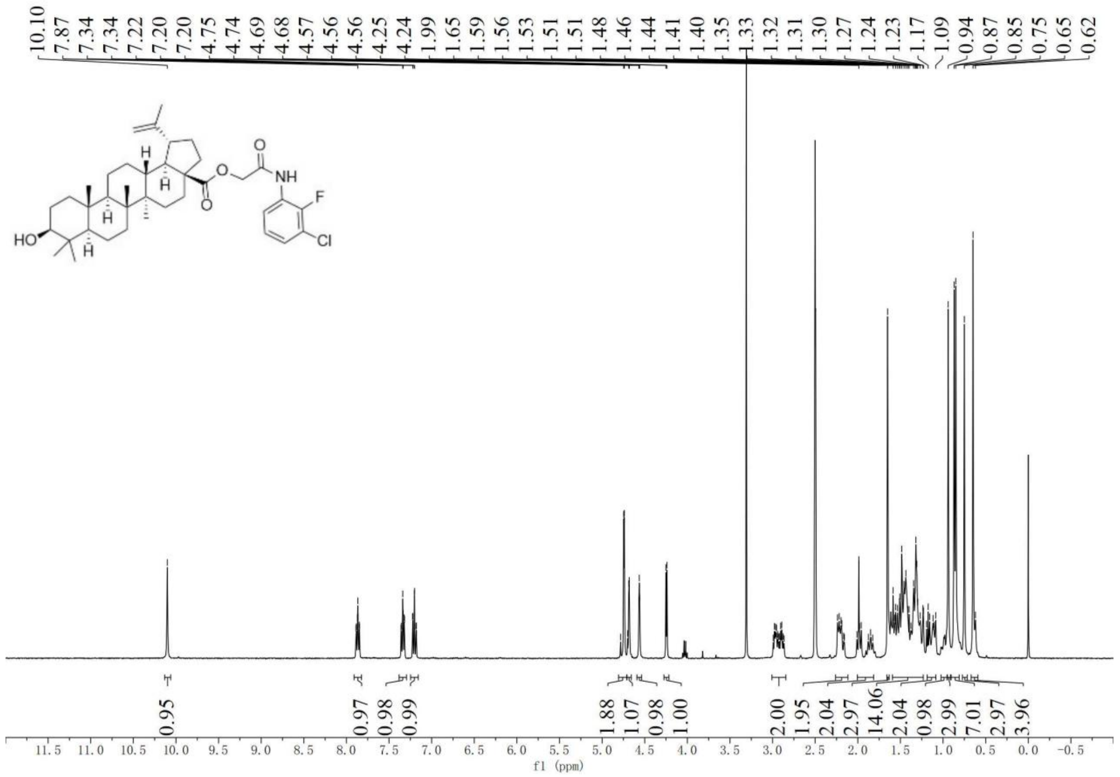

Figure S7-1 ^{1}H NMR spectrum of compound **3g**.

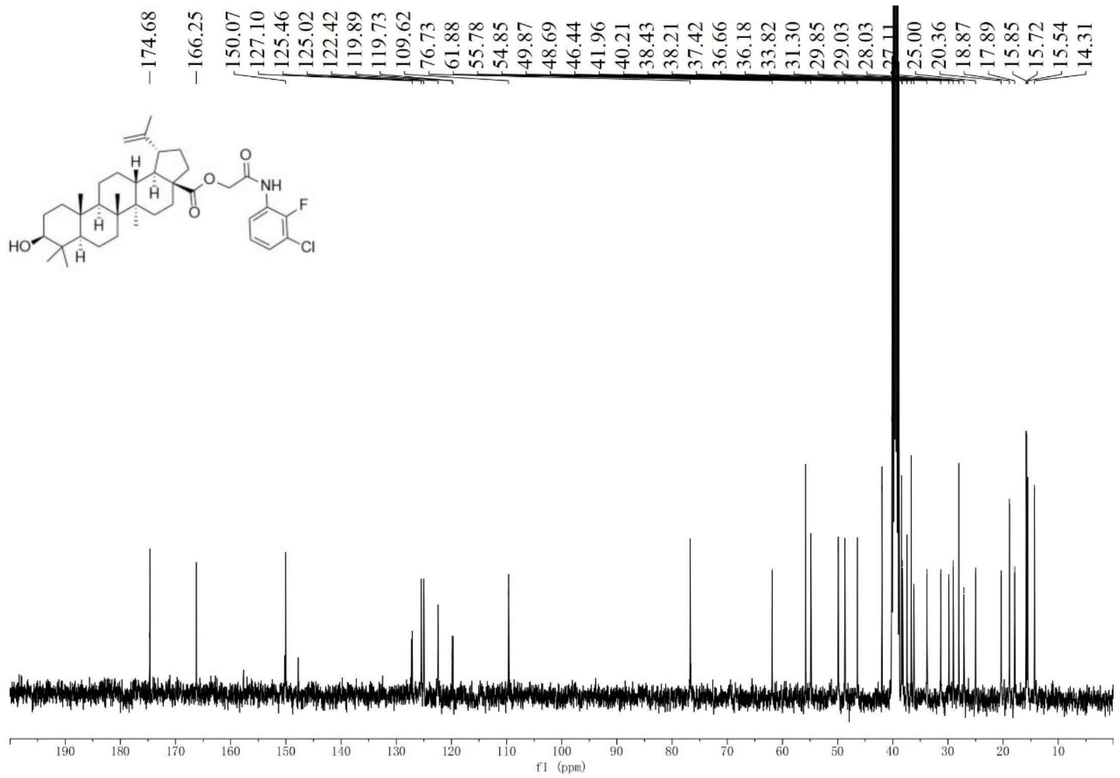

Figure S7-2 ^{13}C NMR spectrum of compound **3g**.

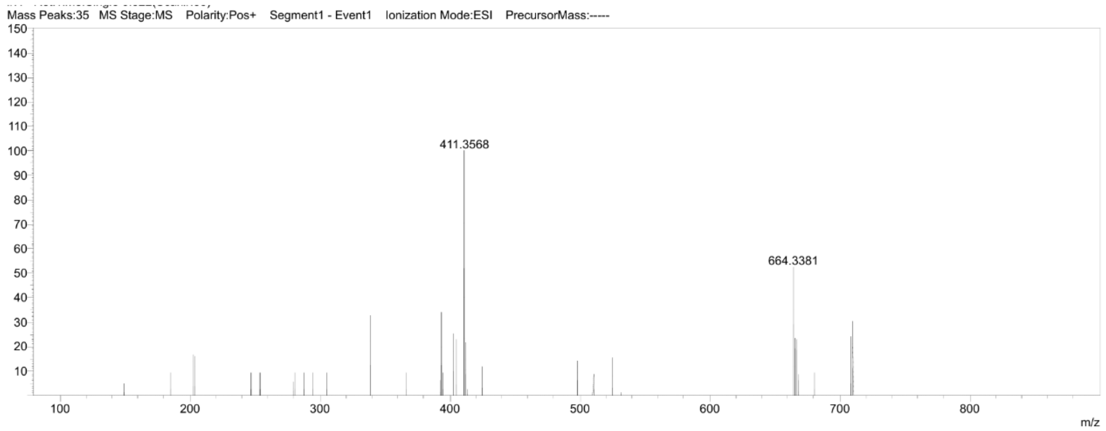

Figure S7-3 HRMS spectrum of compound **3g**.

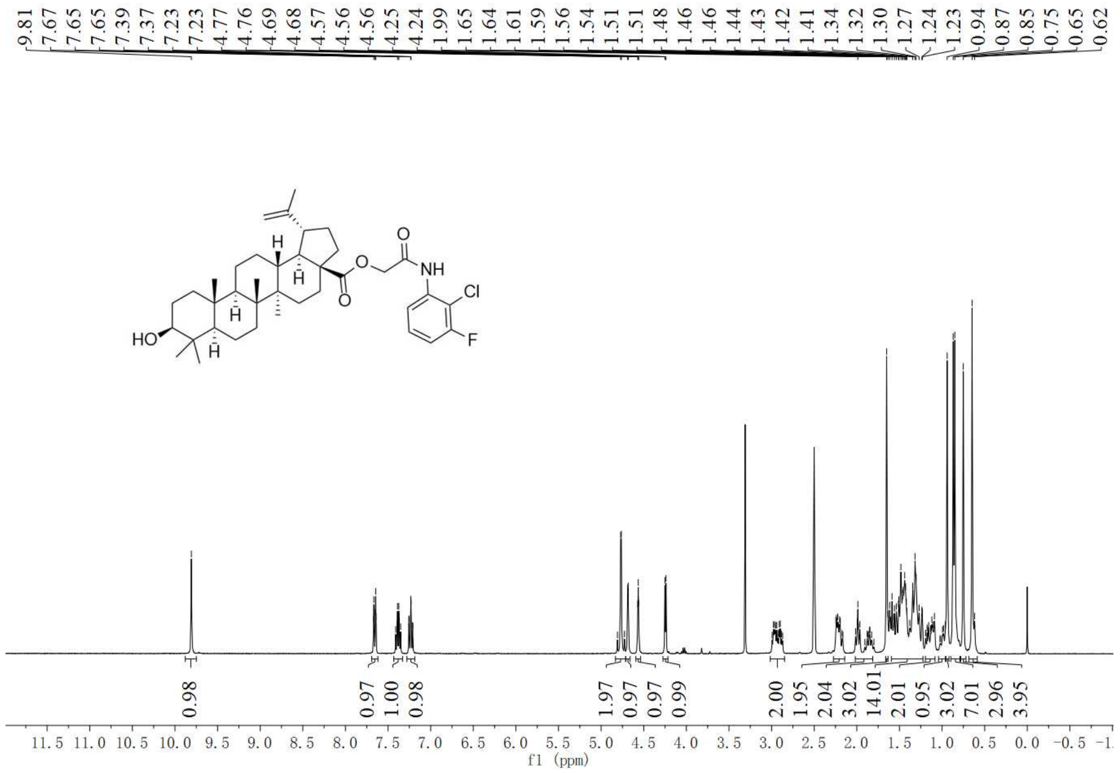

Figure S8-1 ^{1}H NMR spectrum of compound **3h**.

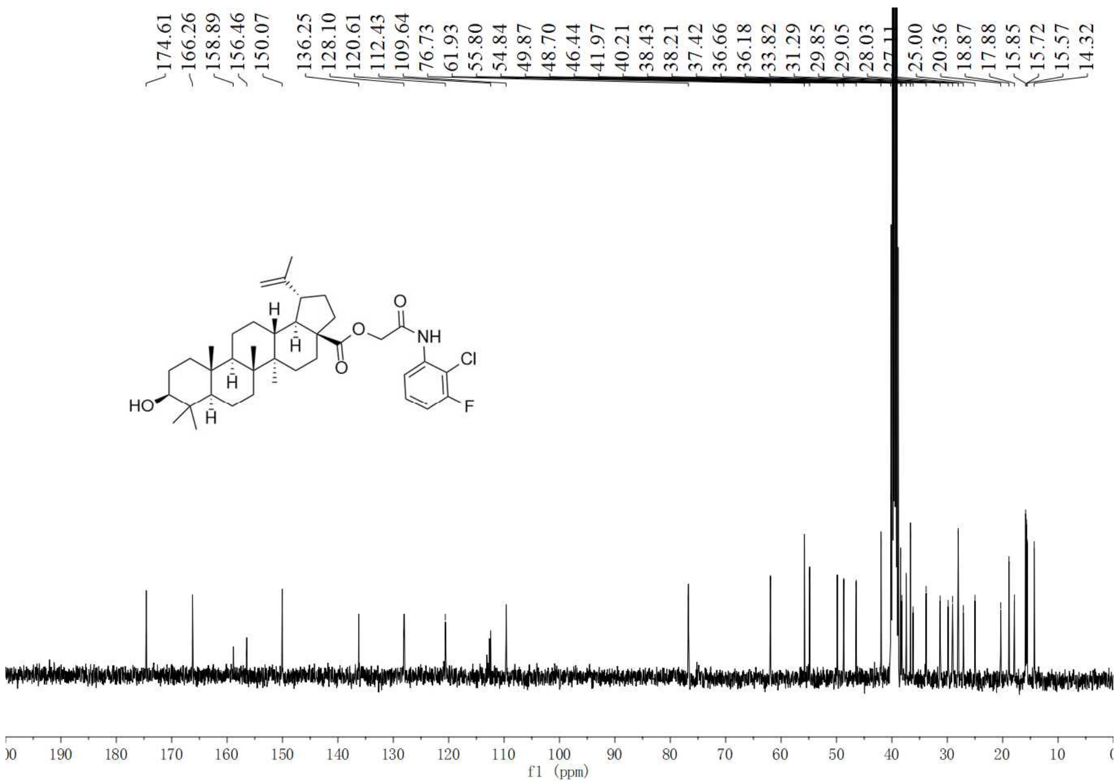

Figure S8-2 ^{13}C NMR spectrum of compound **3h**.

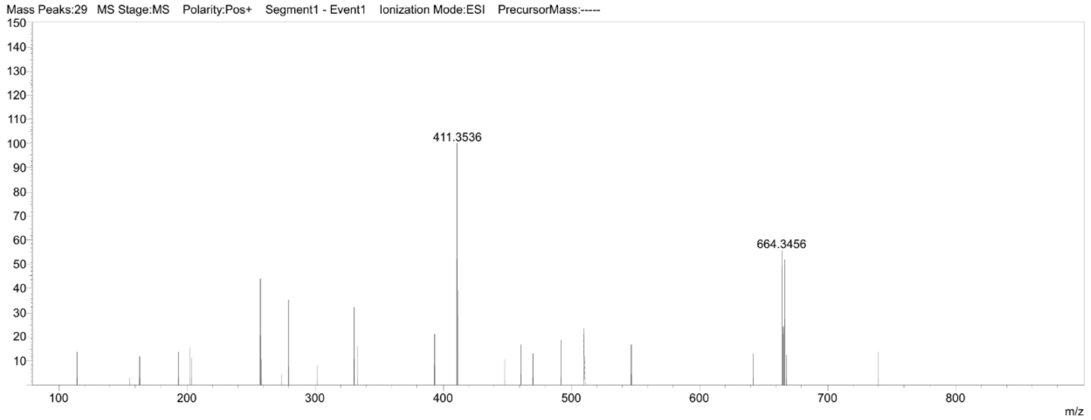

Figure S8-3 HRMS spectrum of compound **3h.**

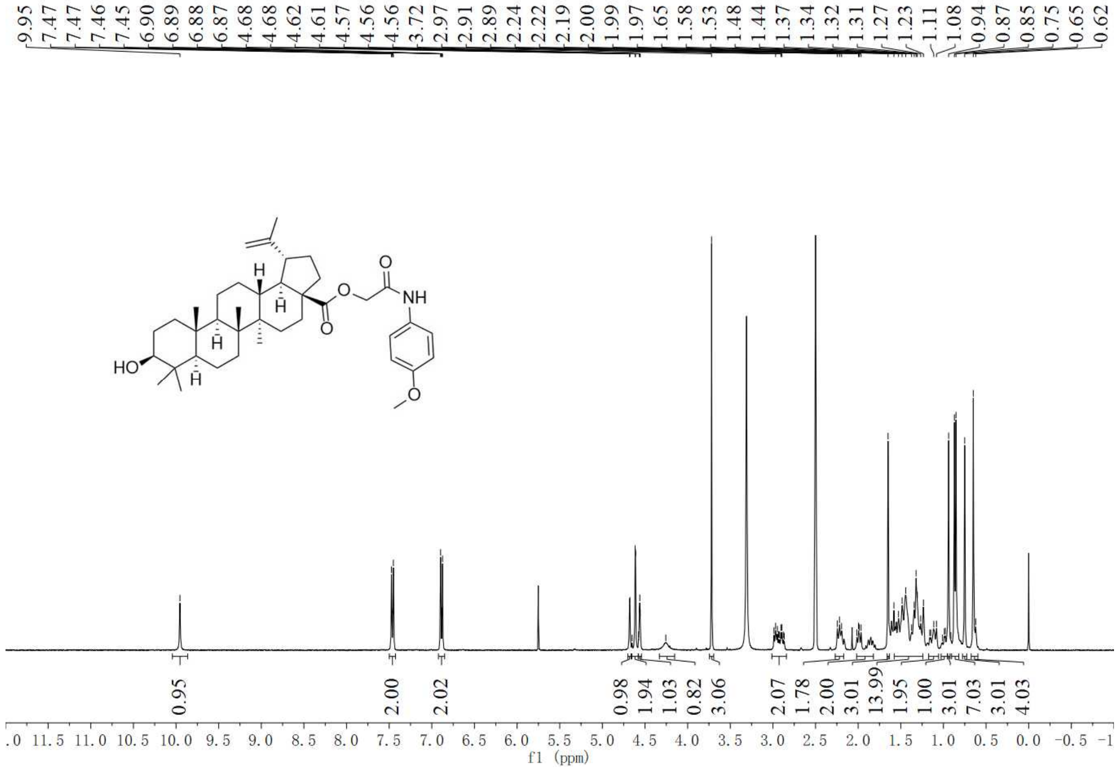

Figure S9-1 ^{1}H NMR spectrum of compound **3i**.

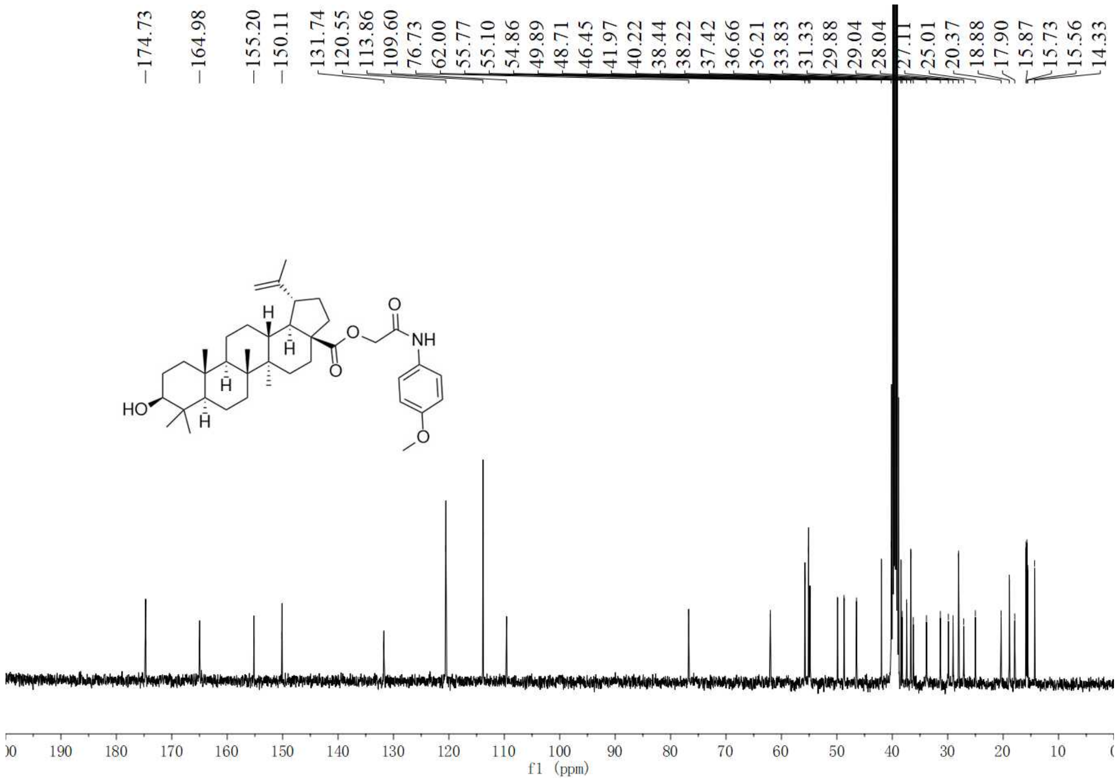

Figure S9-2 ^{13}C NMR spectrum of compound **3i**.

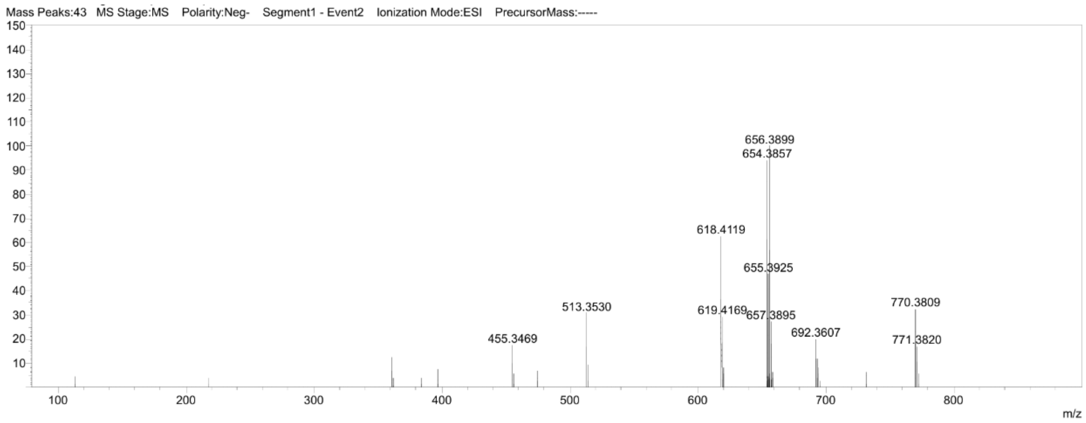

Figure S9-3 HRMS spectrum of compound **3i.**

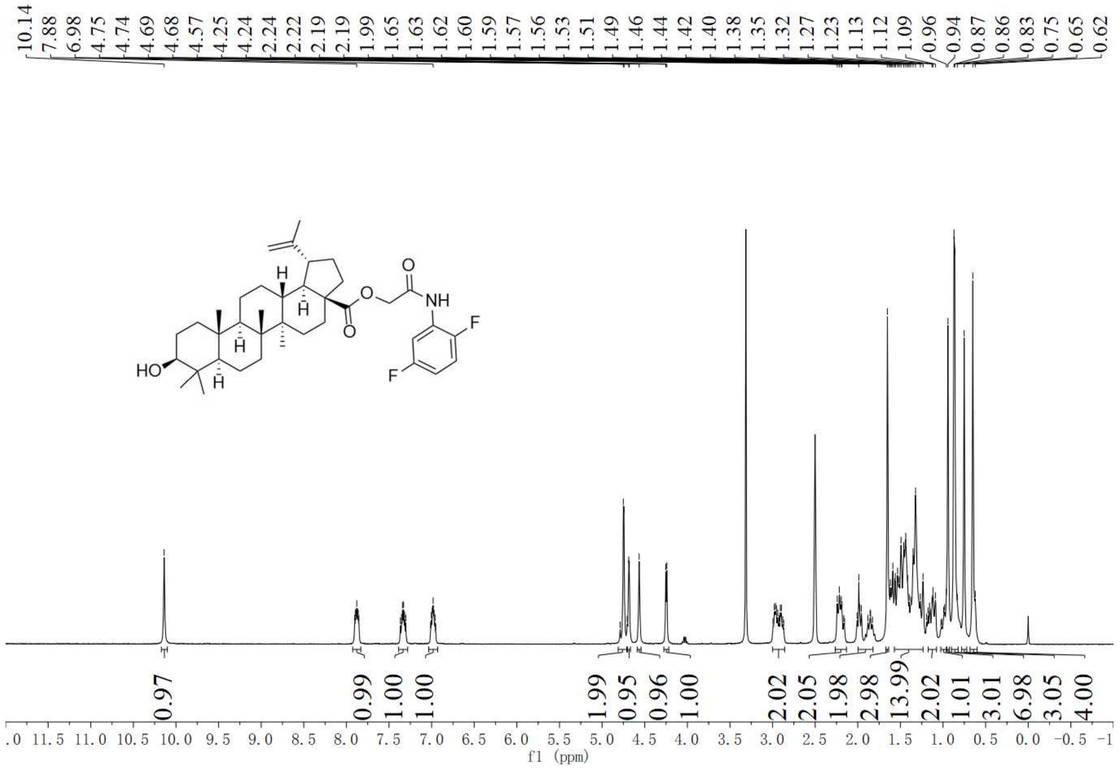

Figure S10-1 ^{1}H NMR spectrum of compound **3j**.

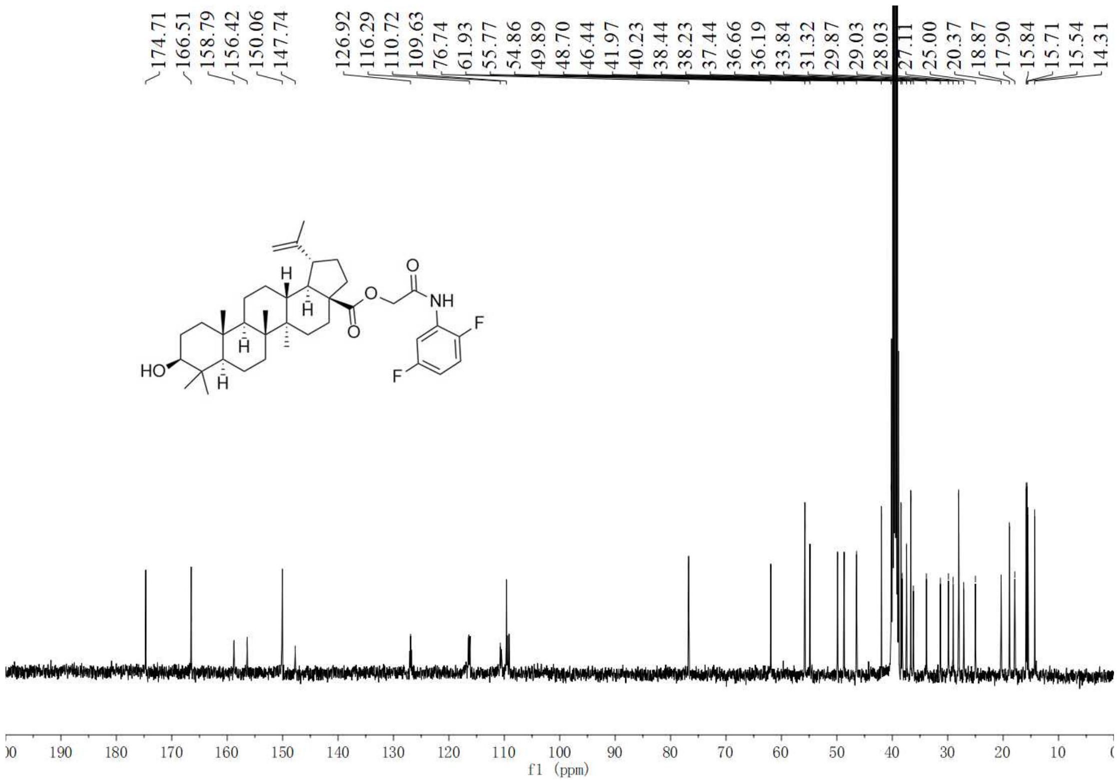

Figure S10-2 ^{13}C NMR spectrum of compound **3j**.

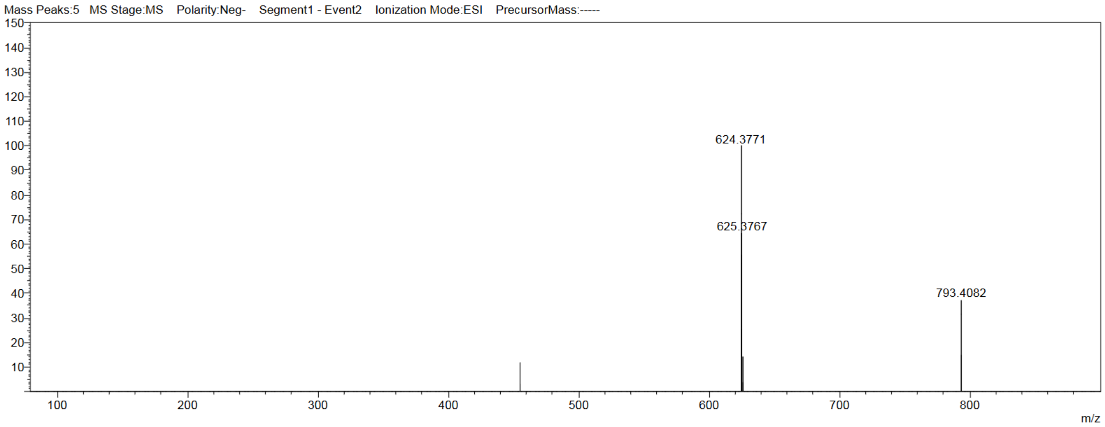

Figure S10-3 HRMS spectrum of compound **3j.**

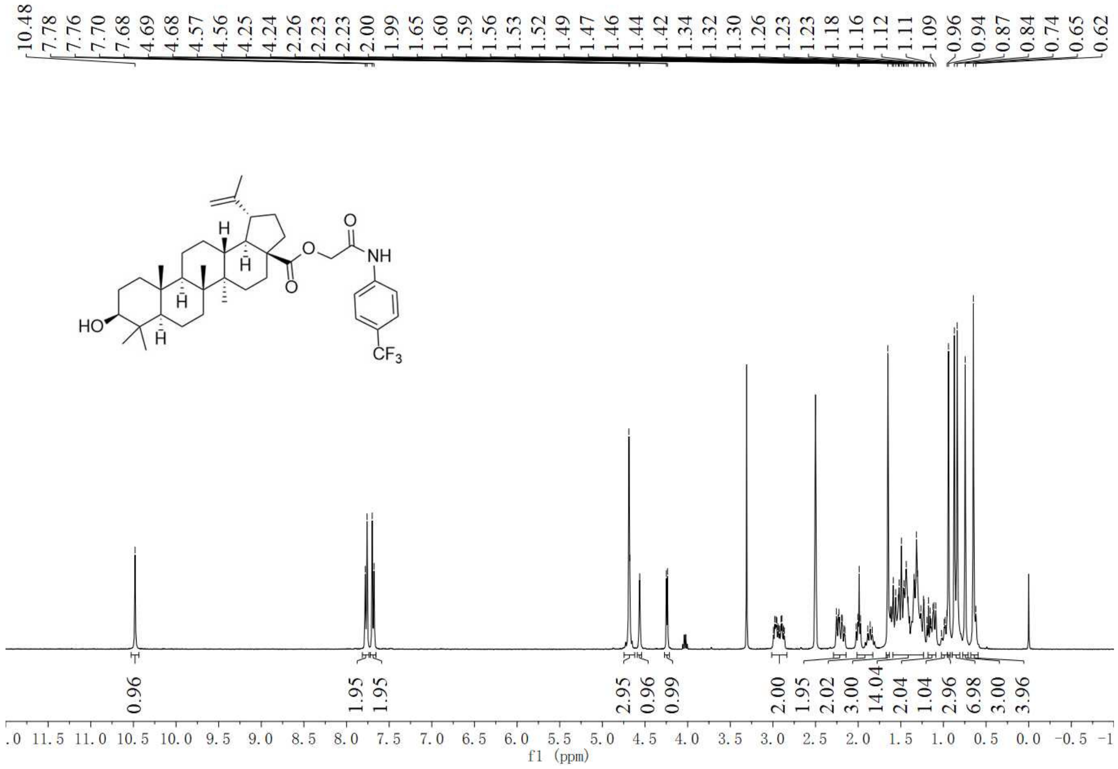

Figure S11-1 ^{1}H NMR spectrum of compound 3k.

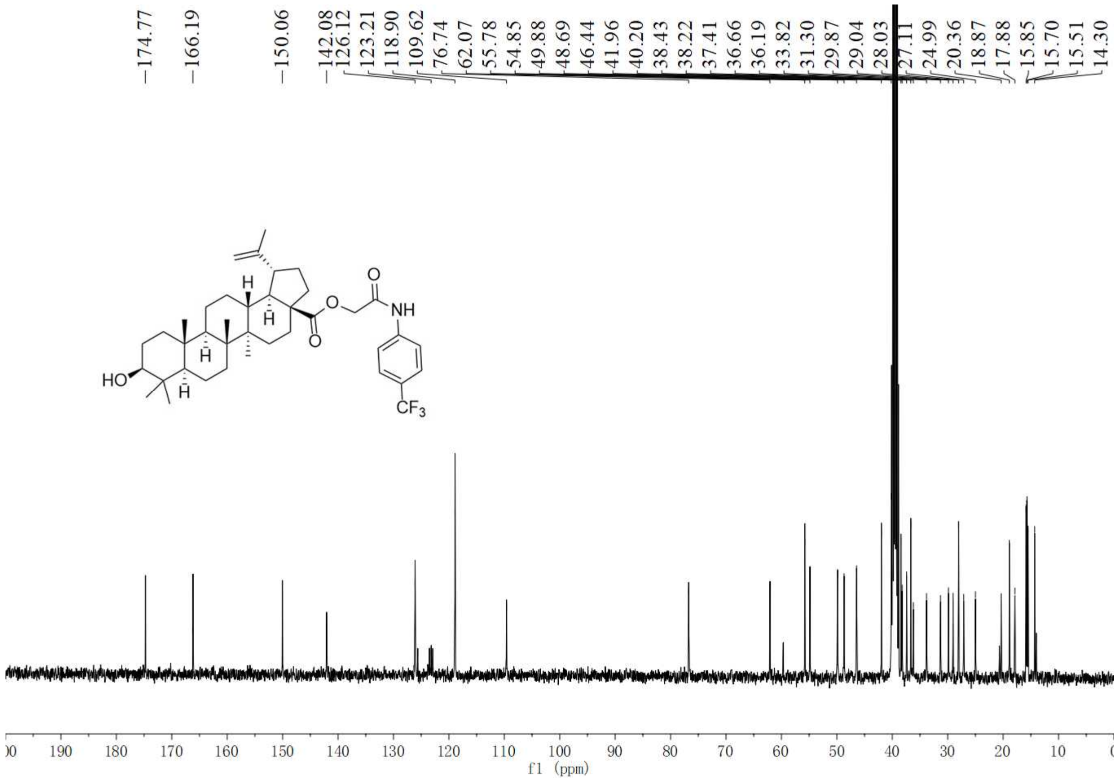

Figure S11-2 ^{13}C NMR spectrum of compound 3k.

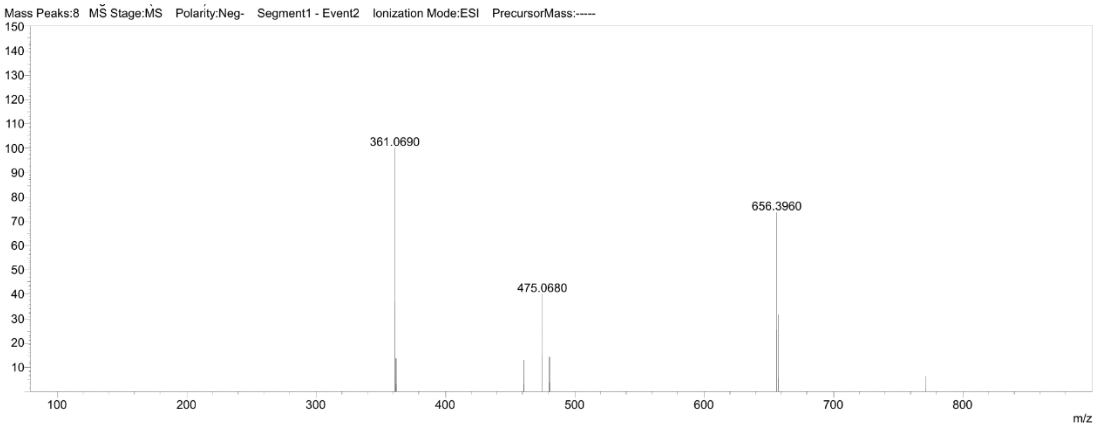

Figure S11-3 HRMS spectrum of compound **3k.**

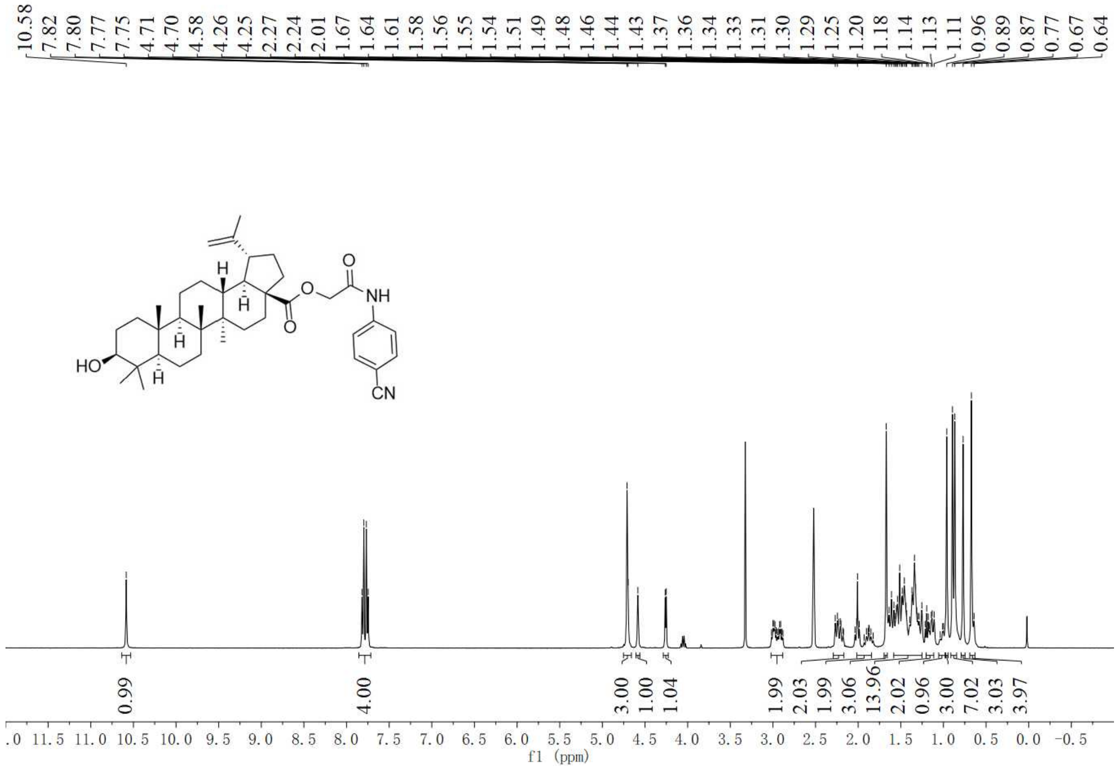

Figure S12-1 ^{1}H NMR spectrum of compound **3l.**

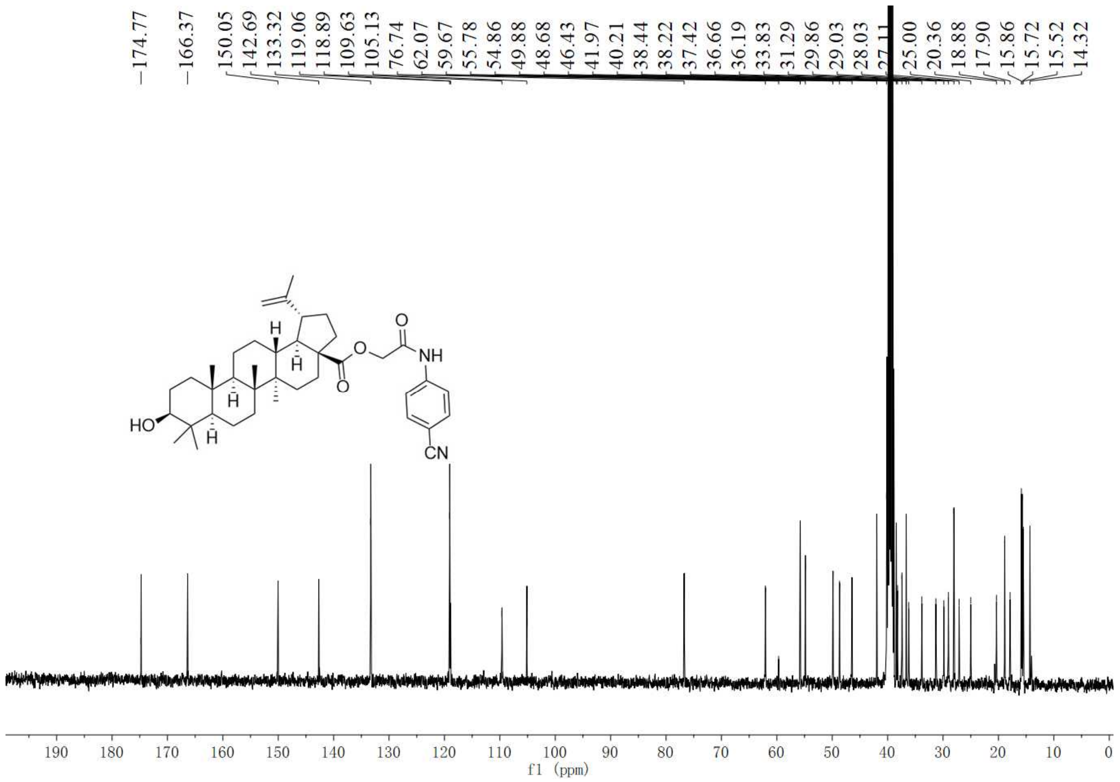

Figure S12-2 ^{13}C NMR spectrum of compound **3l.**

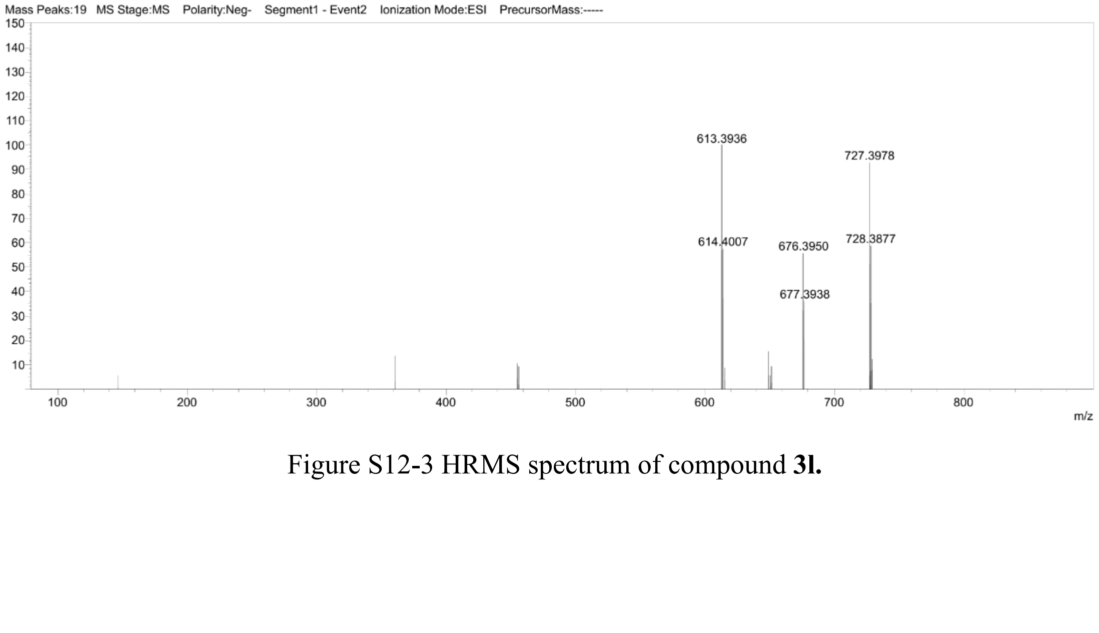

Figure S12-3 HRMS spectrum of compound **3l.**

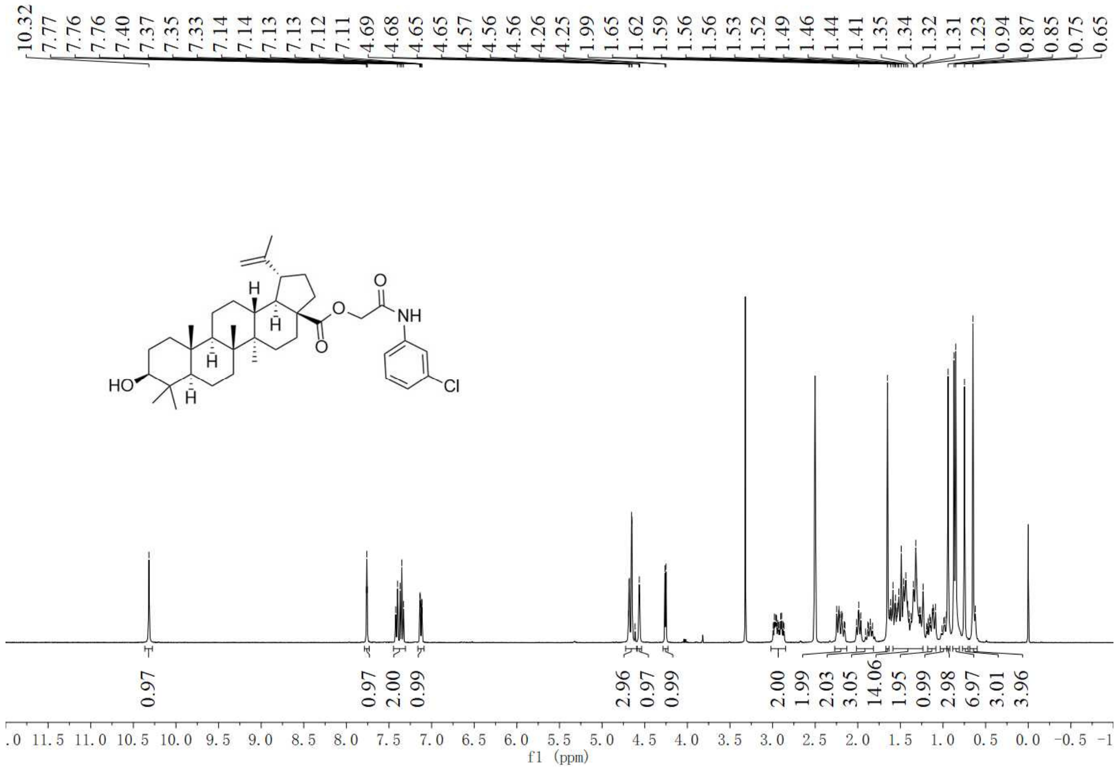

Figure S13-1 ^{1}H NMR spectrum of compound **3m.**

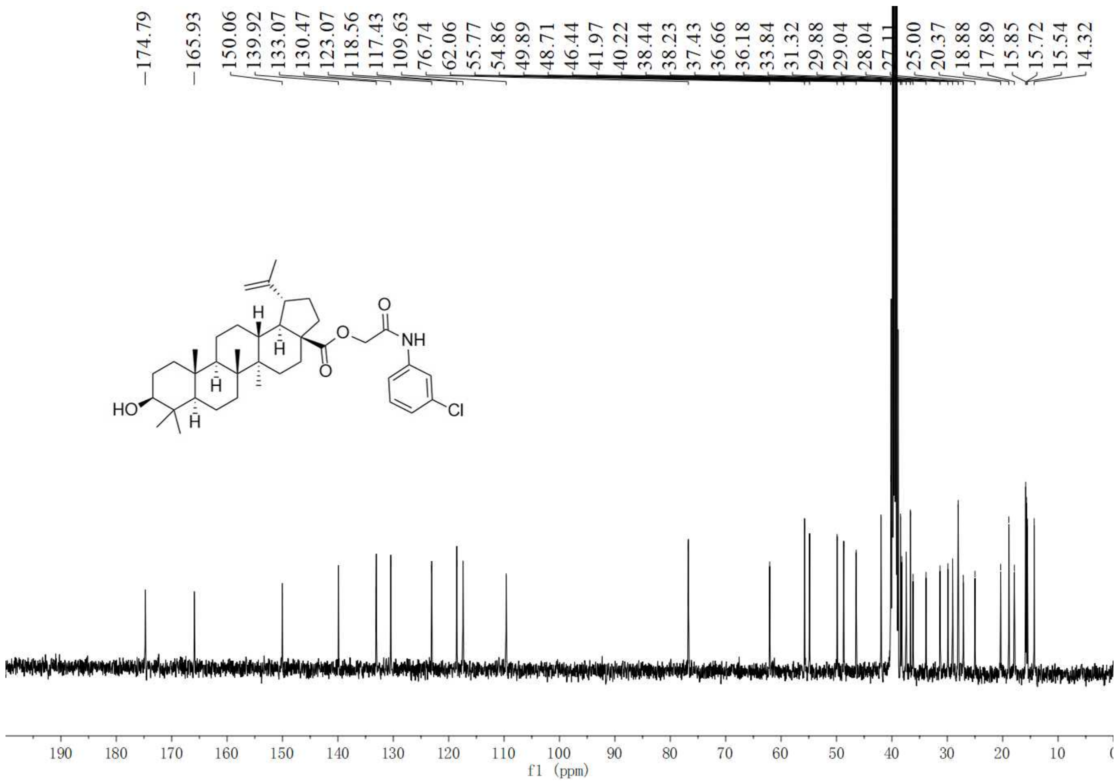

Figure S13-2 ^{13}C NMR spectrum of compound **3m.**

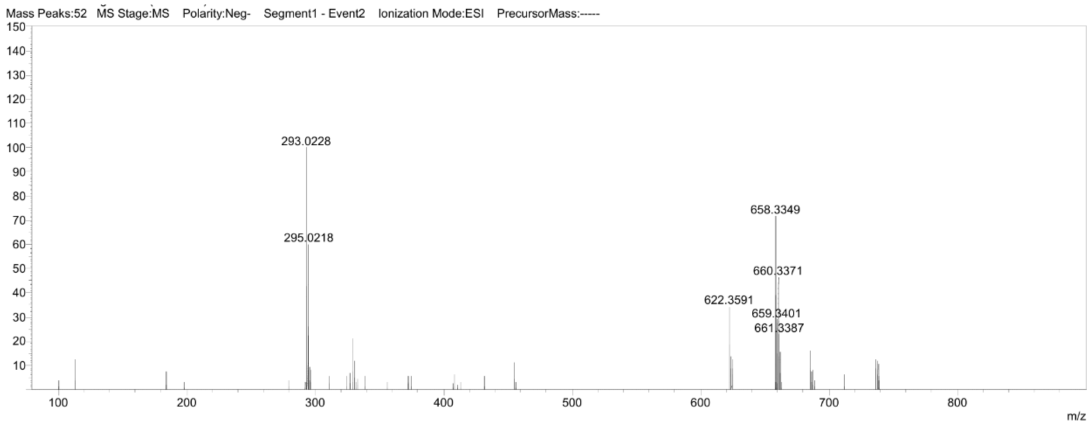

Figure S13-3 HRMS spectrum of compound **3m.**

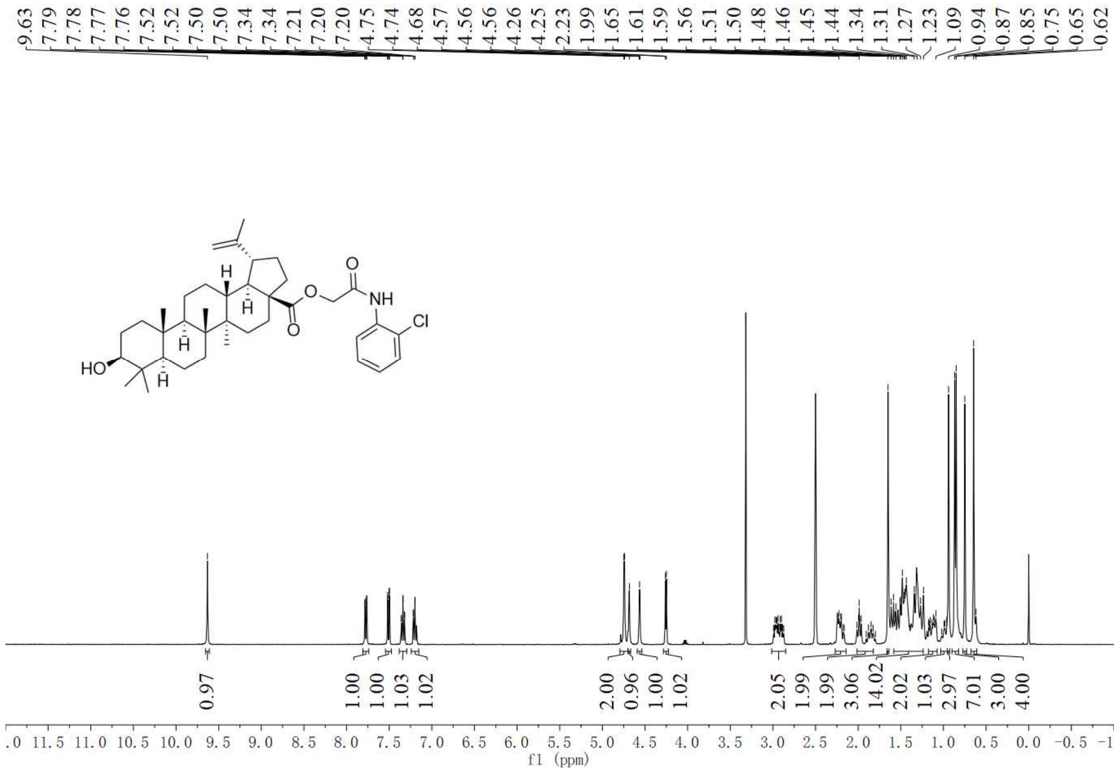

Figure S14-1 ¹H NMR spectrum of compound **3n.**

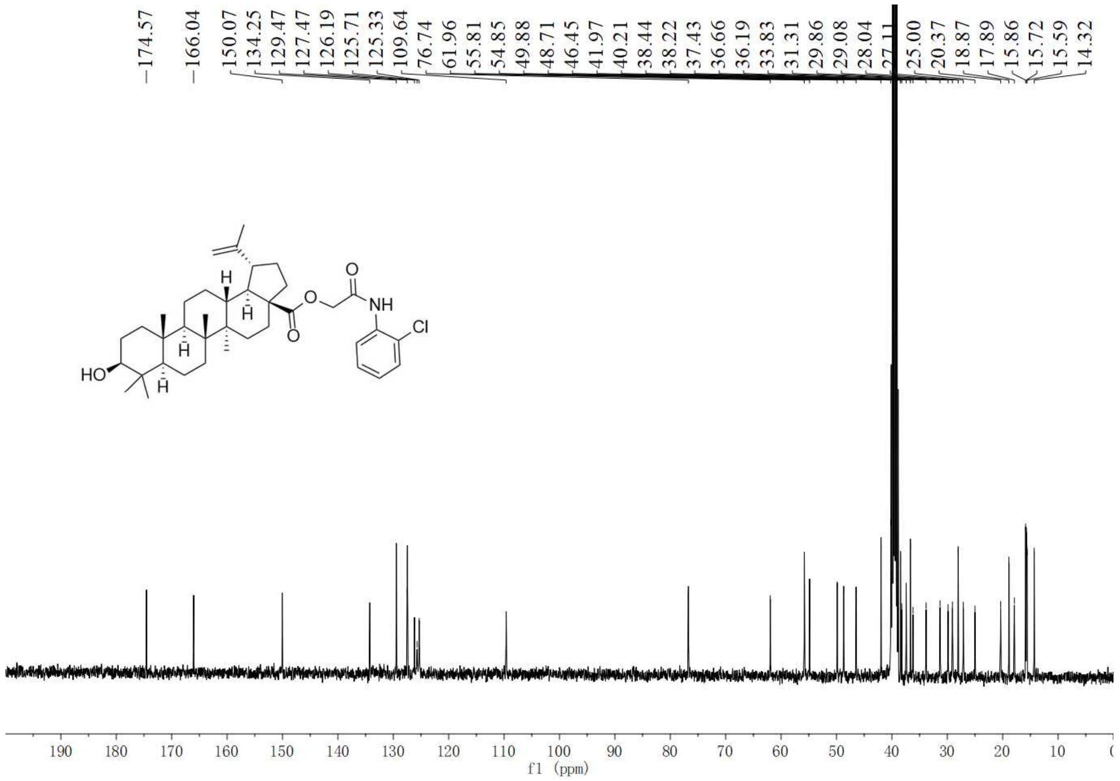

Figure S14-2 ^{13}C NMR spectrum of compound **3n.**

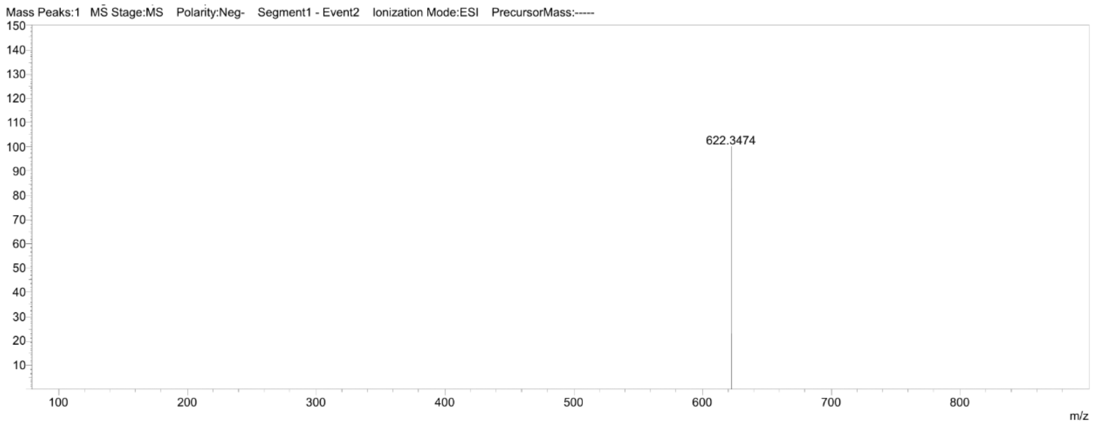

Figure S14-3 HRMS spectrum of compound **3n.**

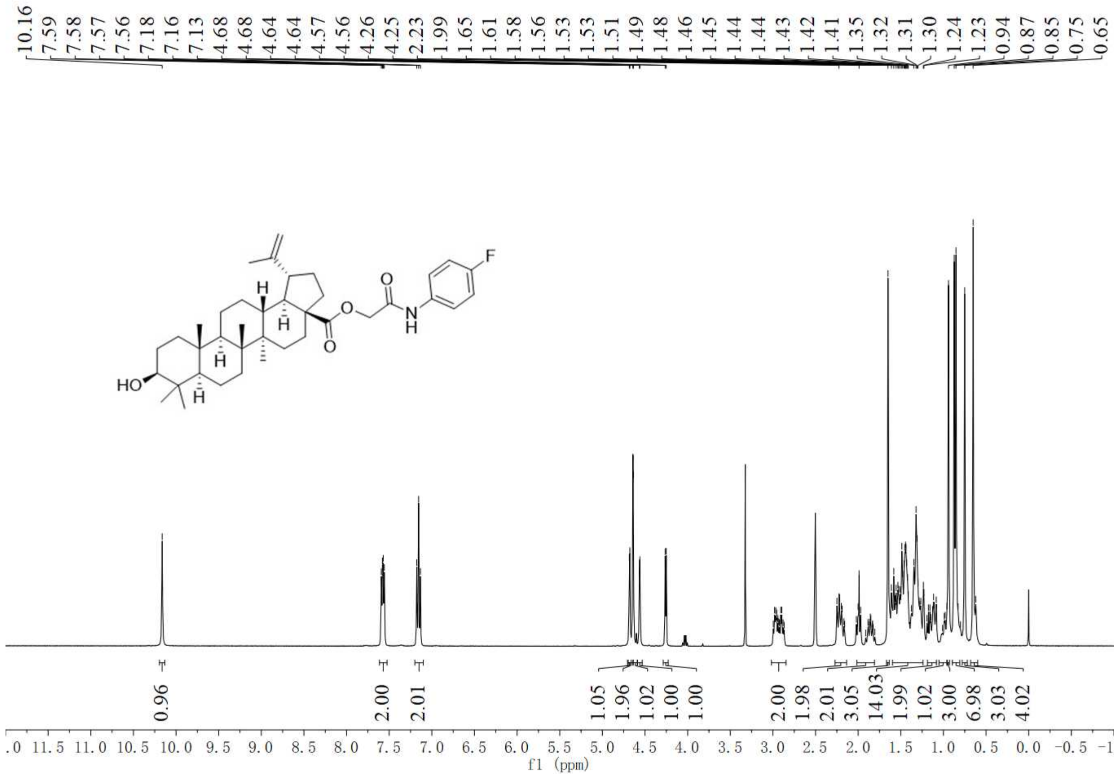

Figure S15-1 ^{1}H NMR spectrum of compound **3o.**

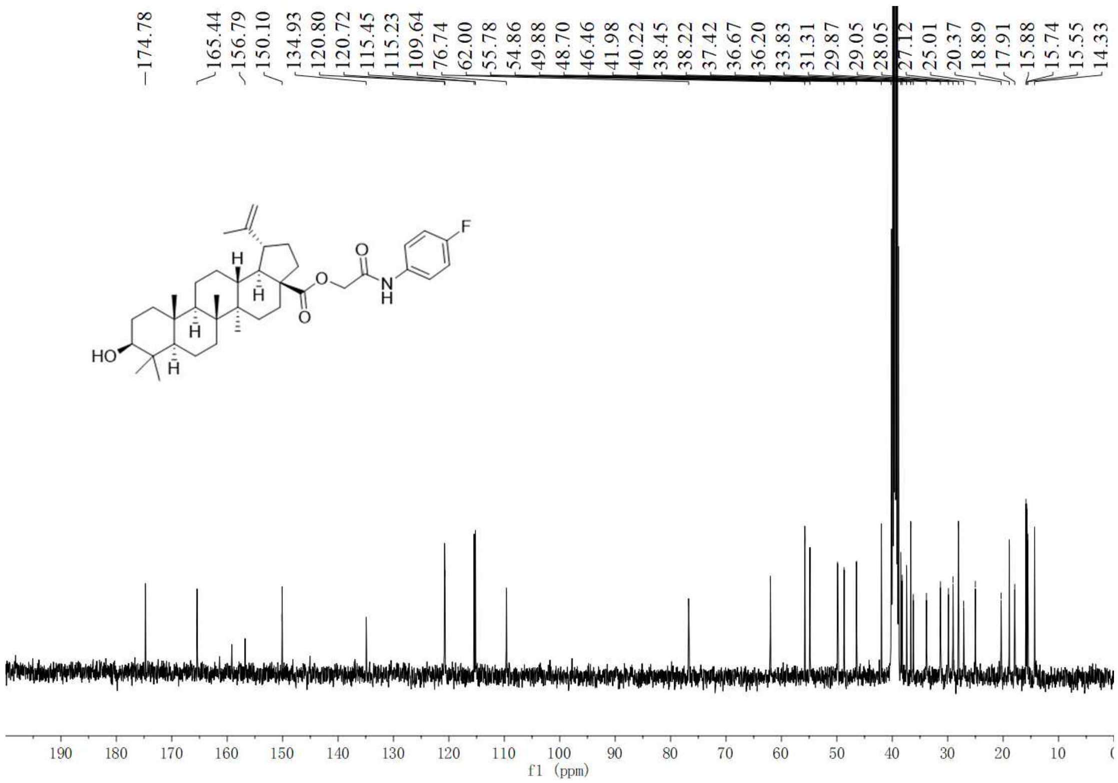

Figure S15-2 ^{13}C NMR spectrum of compound **3o.**

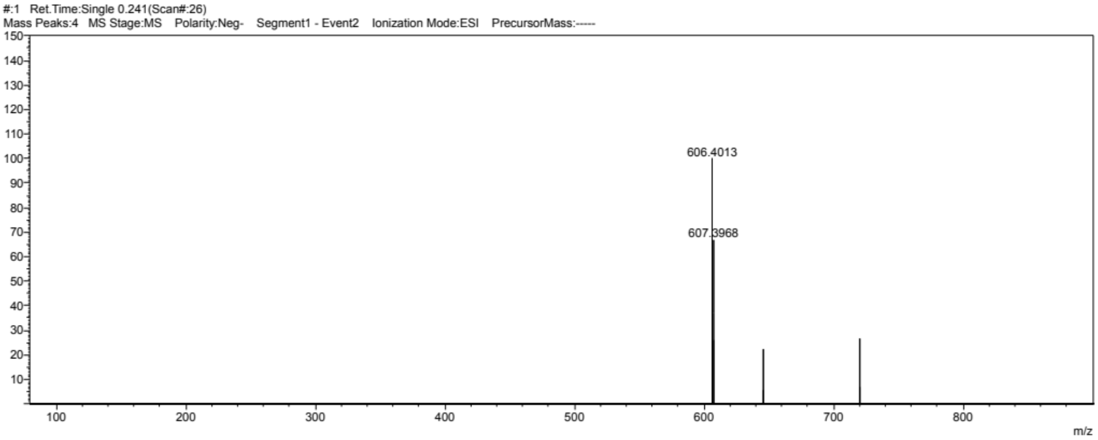

Figure S15-3 HRMS spectrum of compound **3o.**

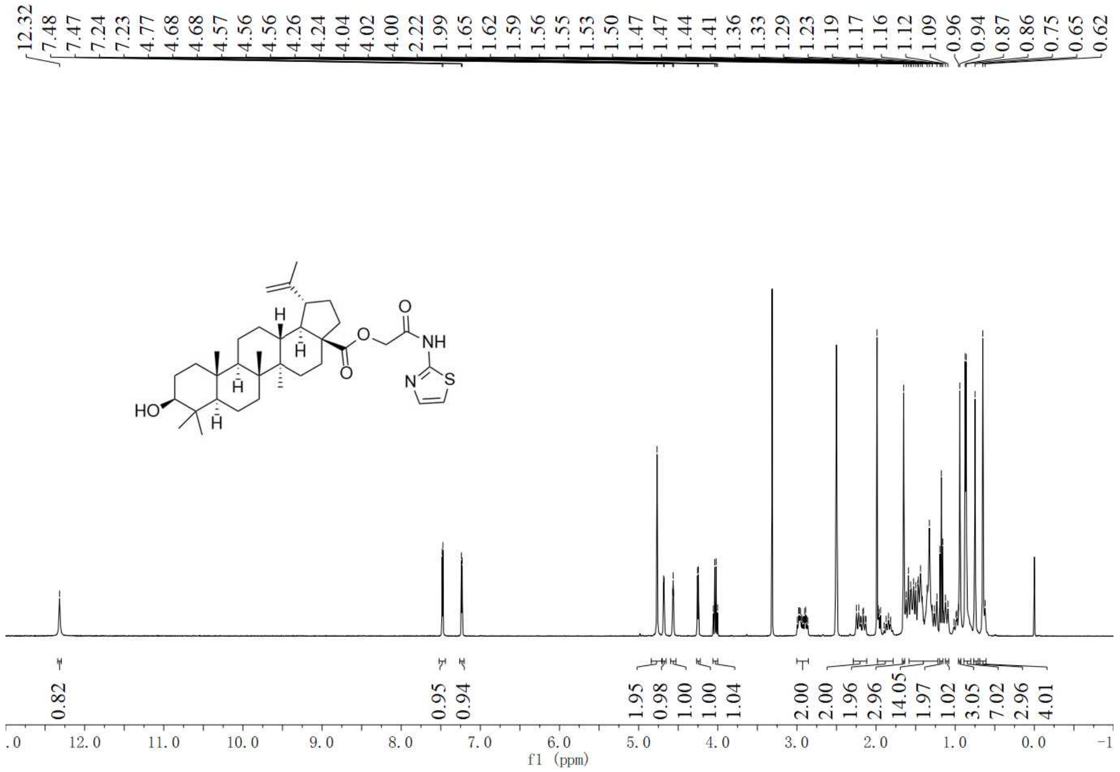

Figure S16-1 ^{1}H NMR spectrum of compound **3p.**

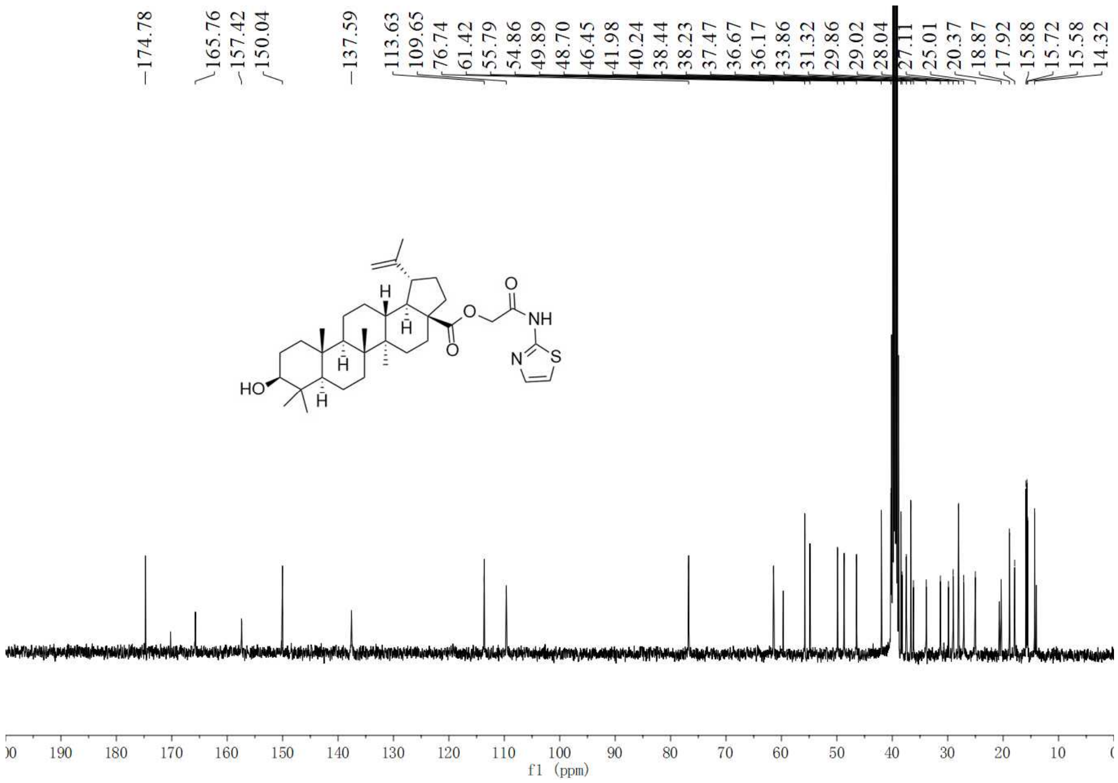

Figure S16-2 ^{13}C NMR spectrum of compound **3p.**

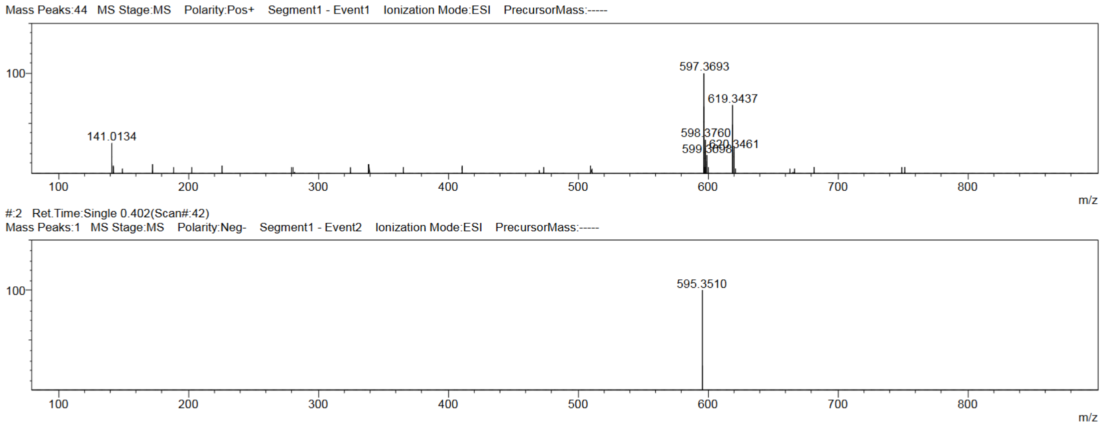

Figure S16-3 HRMS spectrum of compound **3p.**

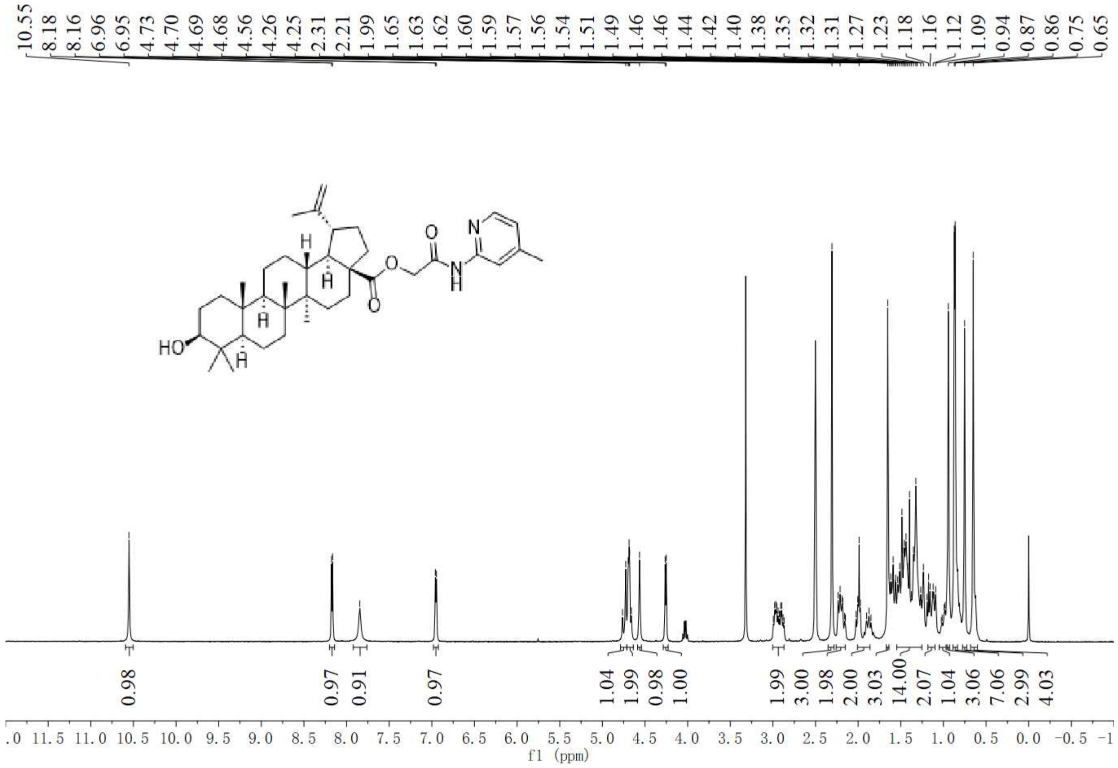

Figure S17-1 ^{1}H NMR spectrum of compound **3q.**

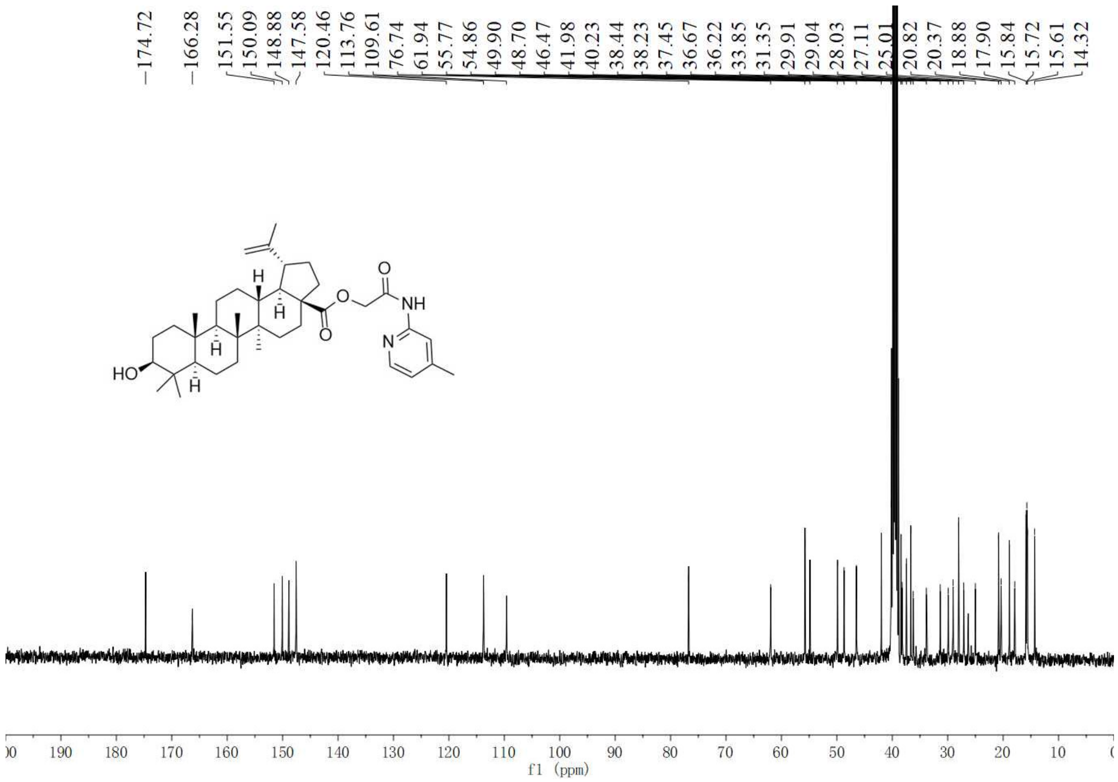

Figure S17-2 ^{13}C NMR spectrum of compound **3q.**

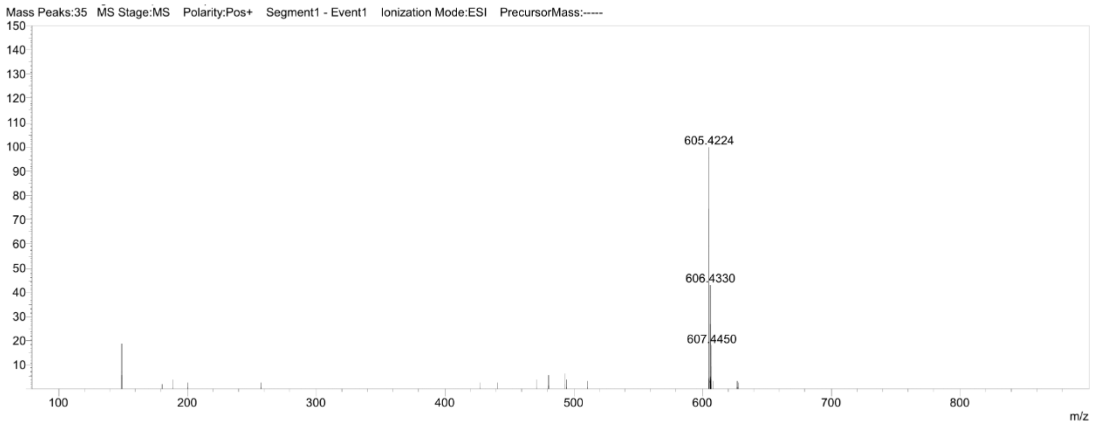

Figure S17-3 HRMS spectrum of compound **3q.**

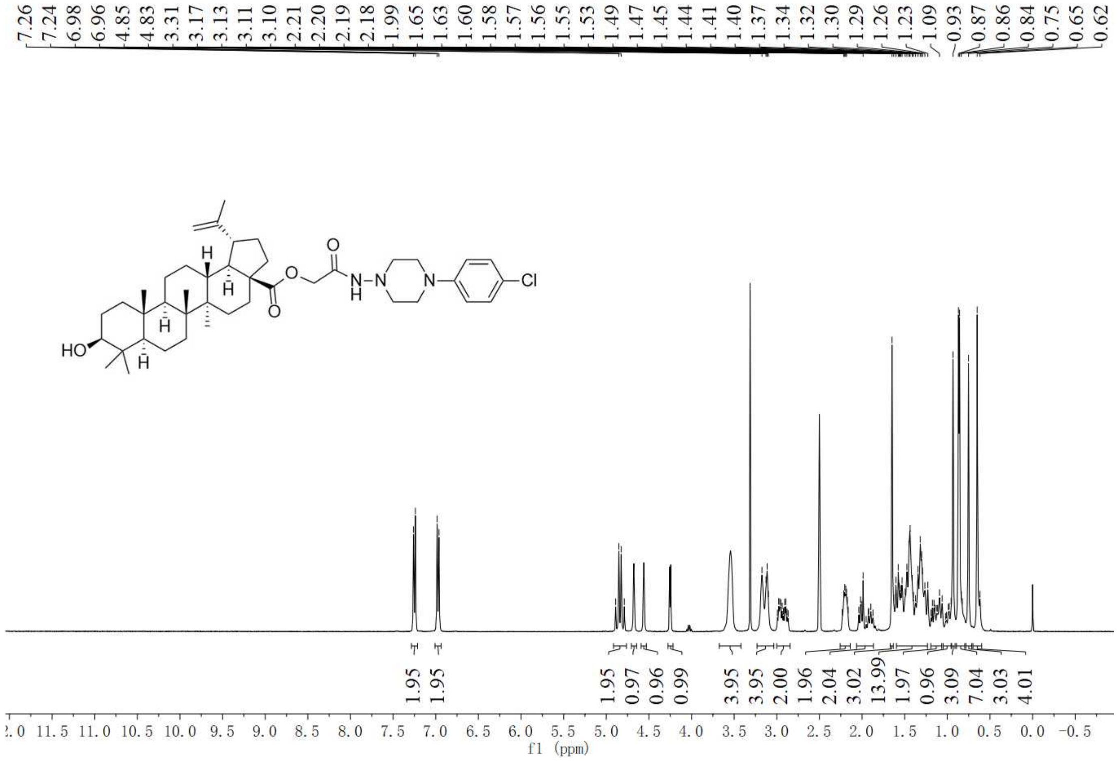

Figure S18-1 ^{1}H NMR spectrum of compound **3r.**

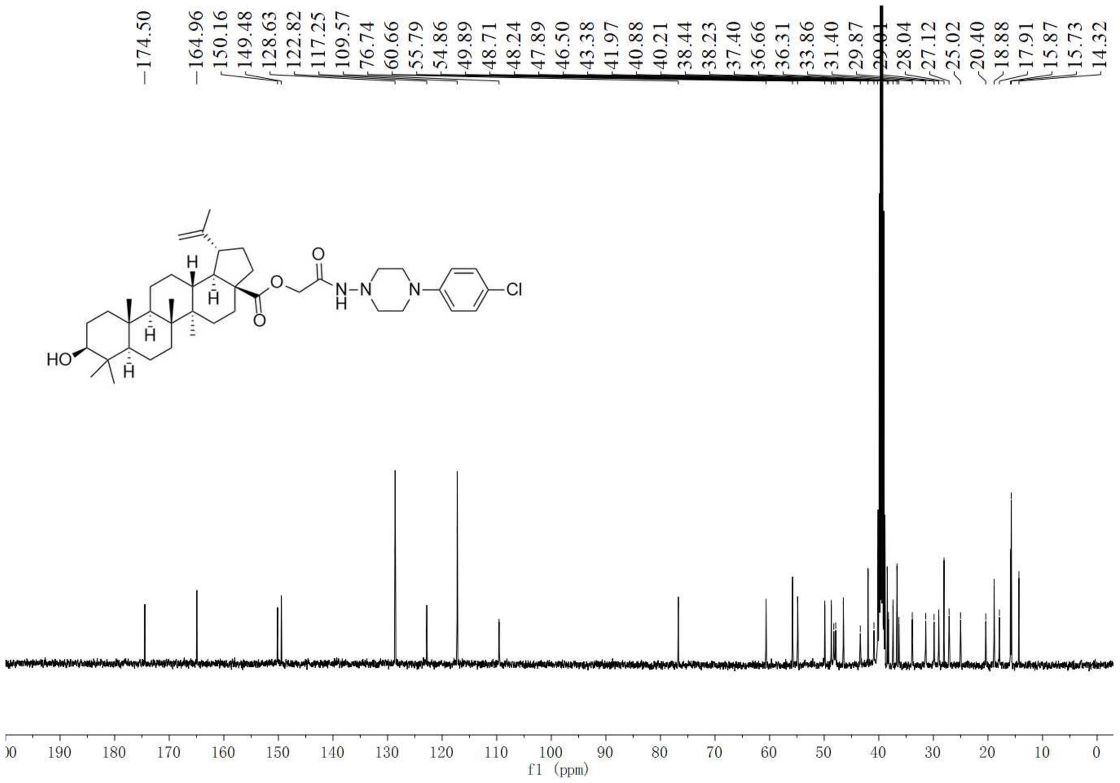

Figure S18-2 ^{13}C NMR spectrum of compound **3r.**

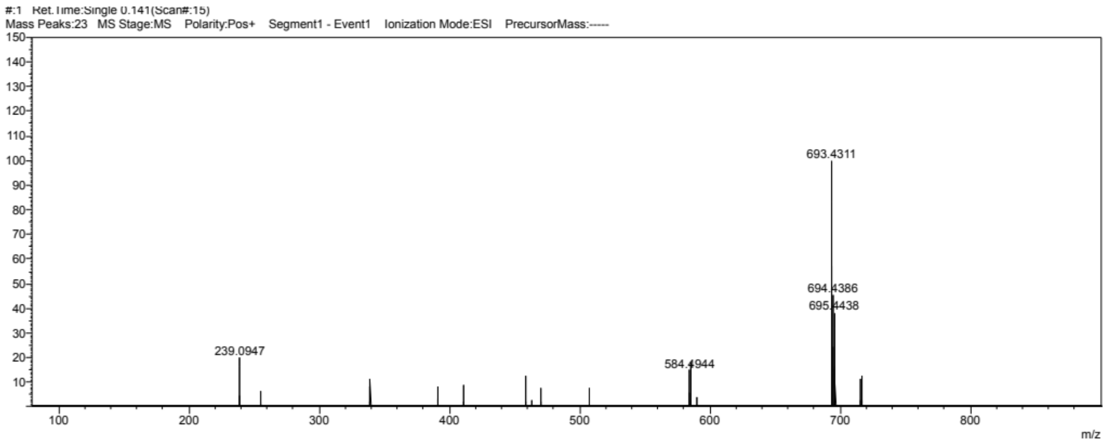

Figure S18-3 HRMS spectrum of compound **3r.**

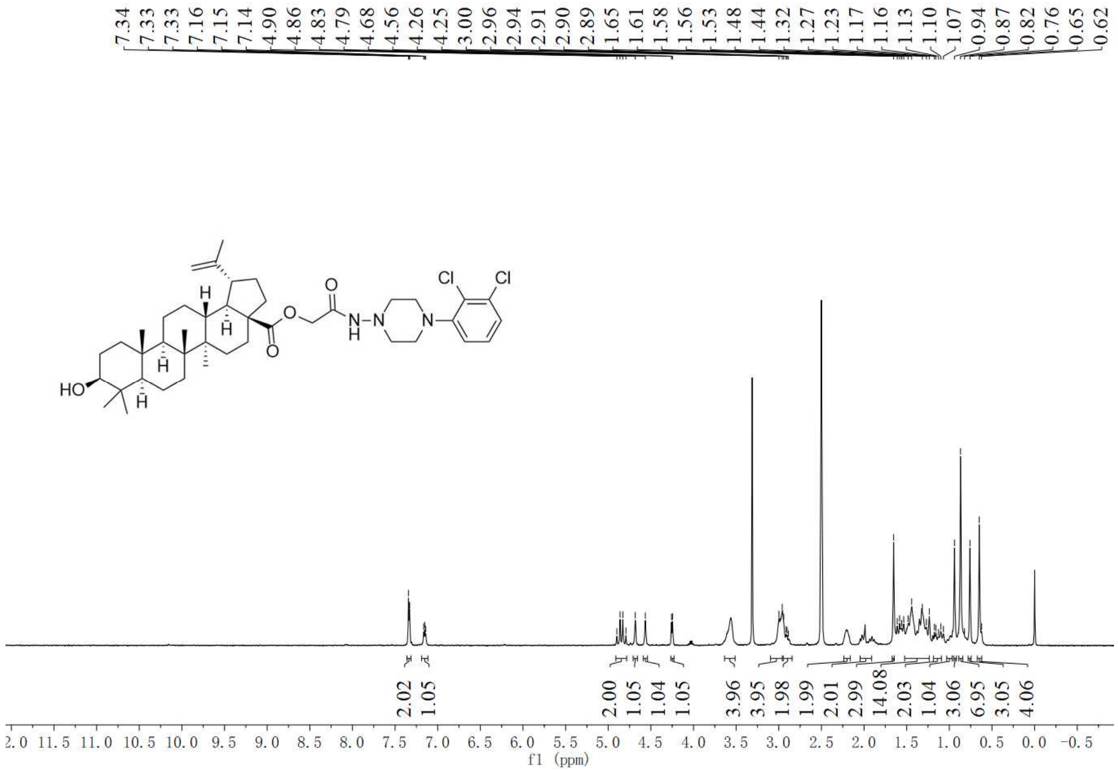

Figure S19-1 ^{1}H NMR spectrum of compound **3s.**

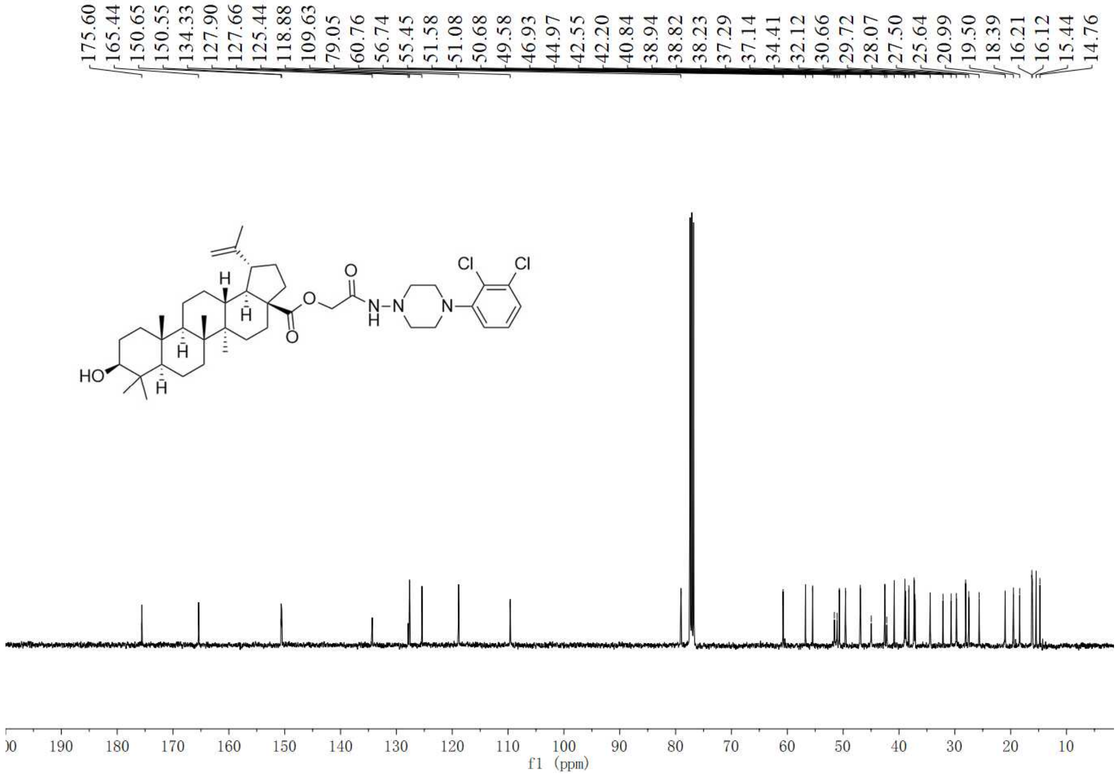

Figure S19-2 ^{13}C NMR spectrum of compound **3s.**

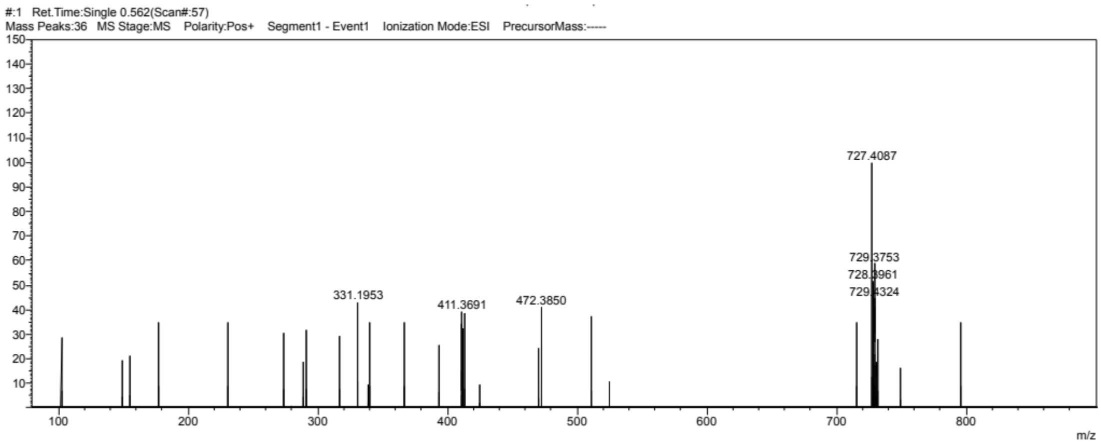

Figure S19-3 HRMS spectrum of compound **3s.**

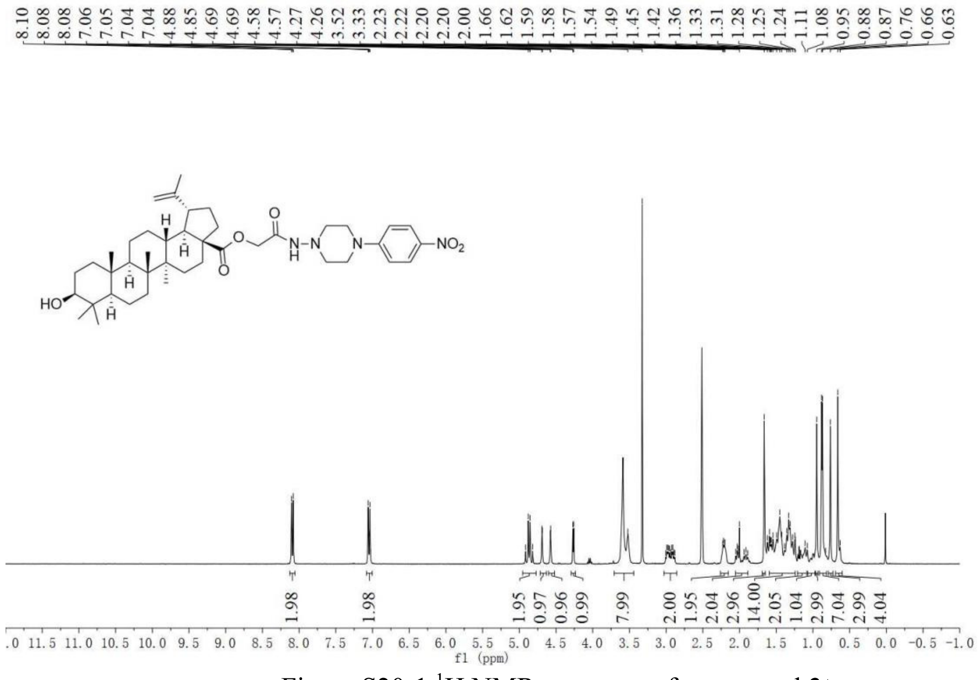

Figure S20-1 ^{1}H NMR spectrum of compound **3t.**

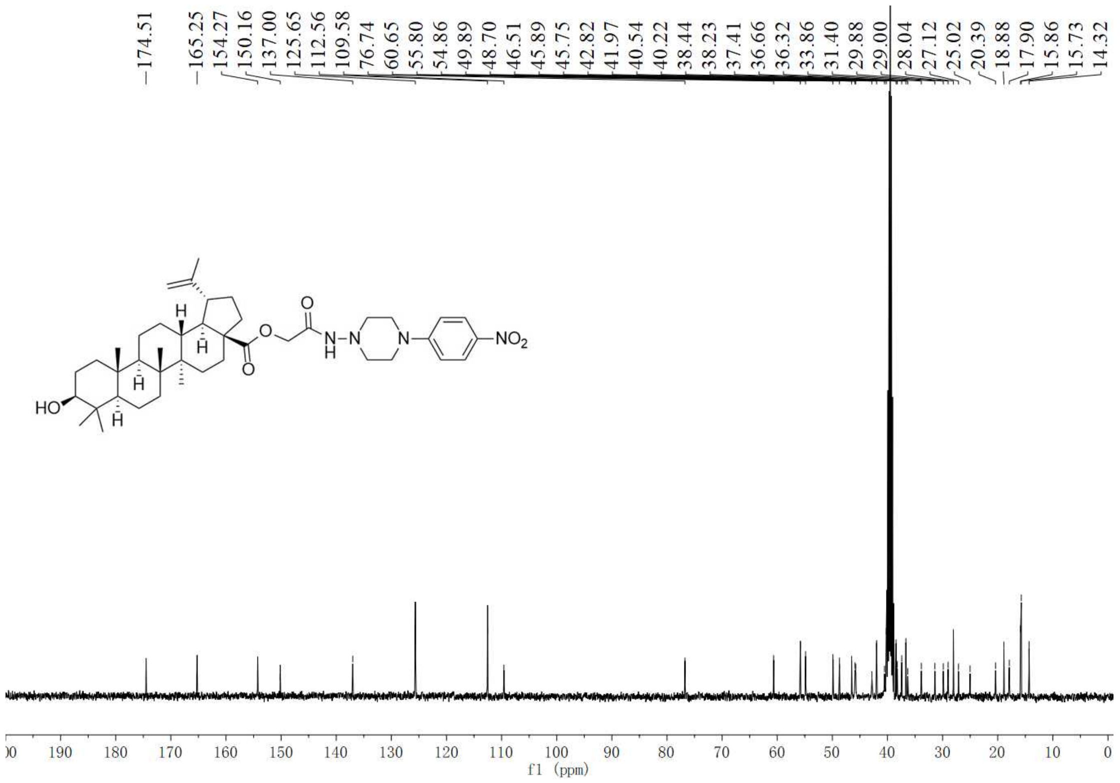

Figure S20-2 ^13^C NMR spectrum of compound **3t.**

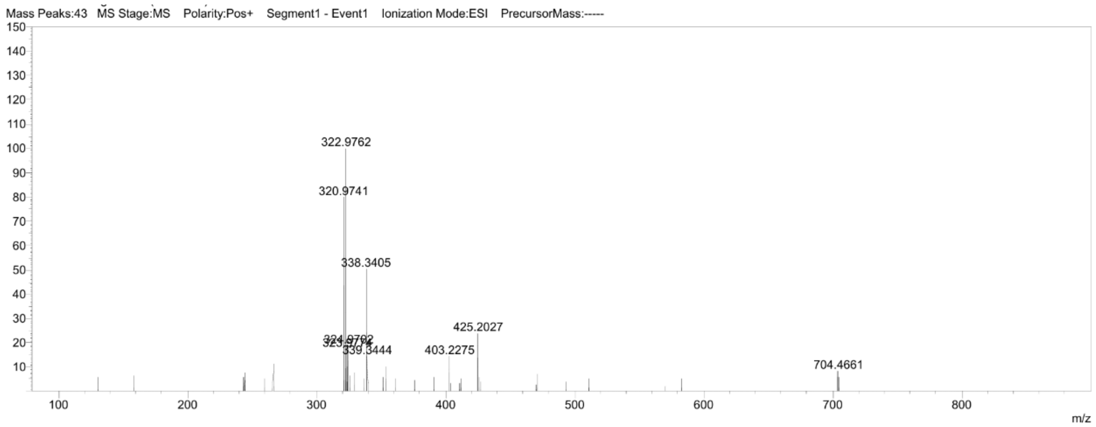

Figure S20-3 HRMS spectrum of compound **3t.**

1.2 Characterization data of Target Compounds 3a to 3t

2-((2-Fluorophenyl)amino)-2-oxoethyl(1*R*,3a*S*,5a*R*,5b*R*,7a*R*,9*S*,11a*R*,11b*R*,13a*R*,13b*R*)-9-hydroxy-5a,5b,8,8,11a-pentamethyl-1-(prop-1-en-2-yl)icosahydro-3a*H*-cyclopenta[a]chrysene-3a-carboxylate (**3a**)

White solid, yield 62%. ^{1}H NMR (400 MHz, DMSO-*d*6) δ 9.90 (s, 1H), 7.92 (td, *J* = 7.7, 3.5 Hz, 1H), 7.31-7.22 (m, 1H), 7.16 (ddd, *J* = 7.2, 4.5, 2.3 Hz, 2H), 4.73 (d, *J* = 4.0 Hz, 2H), 4.68 (d, *J* = 2.6 Hz, 1H), 4.56 (s, 1H), 4.25 (d, *J* = 5.0 Hz, 1H), 2.93 (ddt, *J* = 27.5, 11.1, 5.4 Hz, 2H), 2.20 (ddd, *J* = 15.6, 10.8, 3.3 Hz, 2H), 2.05-1.76 (m, 2H), 1.65 (s, 3H), 1.62-1.21 (m, 14H), 1.18-1.05 (m, 2H), 1.00 (m, 1H), 0.94 (s, 3H), 0.86 (d, *J* = 5.3 Hz, 7H), 0.75 (s, 3H), 0.65 (s, 4H). ^{13}C NMR (101 MHz, DMSO-*d*6) δ 174.67, 166.03, 154.44, 150.09, 125.56, 124.38, 124.34, 123.57, 115.33, 109.63, 76.75, 61.89, 55.79, 54.87, 49.90, 48.70, 46.45, 41.97, 40.22, 38.45, 38.24, 37.42, 36.67, 36.22, 33.84, 31.33, 29.87, 29.05, 28.04, 27.12, 25.02, 20.38, 18.88, 17.91, 15.87, 15.73, 15.56, 14.33. HRMS (ESI) m/z: calculated $C_{38}H_{53}FNO_4$ $[M-H]^-$ 606.3964, found: 606.4041.

3-2-((4-Chloro-3-fluorophenyl)amino)-2-oxoethyl(1*R*,3a*S*,5a*R*,5b*R*,7a*R*,9*S*,11a*R*,11b*R*,13a*R*,13b*R*)-9-hydroxy-5a,5b,8,8,11a-pentamethyl-1-(prop-1-en-2-yl)icosahydro-3a*H*-cyclopenta[*a*]chrysene-3a-carboxylate (**3b**)

White solid, yield 60%. ^{1}H NMR (400 MHz, DMSO-*d*6) δ 10.34 (s, 1H), 7.87 (dd, *J* = 6.6, 2.6 Hz, 1H), 7.50-7.33 (m, 2H), 4.73-4.60 (m, 3H), 4.56 (s, 1H), 4.25 (d, *J* = 5.1 Hz, 1H), 2.93 (ddt, *J* = 28.2, 11.0, 5.3 Hz, 2H), 2.31-2.13 (m, 2H), 1.99 (s, 1H), 1.93-1.79 (m, 1H), 1.65 (s, 3H), 1.62-1.21 (m, 14H), 1.20-1.07 (m, 2H), 1.00 (m, 1H),0.94 (s, 3H), 0.86 (d, *J* = 9.3 Hz, 7H), 0.75 (s, 3H), 0.65 (s, 4H). ^{13}C NMR (101 MHz, DMSO-*d*6) δ 174.77, 165.85, 151.91, 150.06, 135.75, 120.48, 119.30, 117.09, 116.88, 109.64, 76.75, 62.00, 55.77, 54.87, 49.89, 48.71, 46.44, 41.97, 40.22, 38.44, 38.24, 37.43, 36.66, 36.19, 33.85, 31.32, 29.89, 29.05, 28.03, 27.12, 25.00, 20.38, 18.88, 17.90, 15.85, 15.72, 15.54, 14.32. HRMS (ESI) m/z: calculated $C_{38}H_{52}ClFNO_4$ $[M-H]^-$ 640.3574, found: 640.3603.

4-2-((4-Chloro-2-fluorophenyl)amino)-2-oxoethyl(1*R*,3a*S*,5a*R*,5b*R*,7a*R*,9*S*,11a*R*,11b*R*,13a*R*,13b*R*)-9-hydroxy-5a,5b,8,8,11a-pentamethyl-1-(prop-1-en-2-yl)icosahydro-3a*H*-cyclopenta[*a*]chrysene-3a-carboxylate (**3c**)

White solid, yield 55%. ^{1}H NMR (400 MHz, DMSO-*d*6) δ 10.17 (s, 1H), 8.09 (dd, *J* = 6.9, 2.7 Hz, 1H), 7.37 (dd, *J* = 10.6, 8.8 Hz, 1H), 7.28-7.19 (m, 1H), 4.76 (d, *J* = 3.4 Hz, 2H), 4.70 (s, 1H), 4.59 (s, 1H), 4.27 (d, *J* = 5.1 Hz, 1H), 2.95 (ddt, *J* = 28.2, 11.0, 5.4 Hz, 2H), 2.28-2.16 (m, 2H), 2.05-1.82 (m, 2H), 1.67 (s, 3H), 1.65-1.24 (m, 14H), 1.14 (t, *J* = 14.1 Hz, 2H), 1.02 (m, 1H), 0.96 (s, 3H), 0.89 (d, *J* = 2.4 Hz, 7H), 0.77 (s, 3H), 0.67 (s, 4H). ^{13}C NMR (101 MHz, DMSO-*d*6) δ 174.74, 166.53, 152.74, 150.05, 127.96, 127.14, 124.40, 122.33, 117.12, 109.63, 76.74, 61.95, 55.76, 54.86, 49.89, 48.71, 46.44, 41.97, 40.23, 38.44, 38.22, 37.42, 36.66, 36.17, 33.84, 31.34, 29.88, 29.03, 28.03, 27.11, 25.00, 20.38, 18.88, 17.89, 15.83, 15.72, 15.57, 14.32. HRMS (ESI) m/z: calculated $C_{38}H_{52}ClFNO_4$ $[M-H]^-$ 640.3574, found: 640.3557.

2-((4-Fluoro-3-(trifluoromethyl)phenyl)amino)-2-

oxoethyl(1*R*,3a*S*,5a*R*,5b*R*,7a*R*,9*S*,11a*R*,11b*R*,13a*R*,13b*R*)-9-hydroxy-5a,5b,8,8,11a-pentamethyl-1-(prop-1-en-2-yl)icosahydro-3a*H*-cyclopenta[*a*]chrysene-3a-carboxylate (**3d**)

White solid, yield 58%. ^{1}H NMR (400 MHz, DMSO-*d*$_6$) δ 10.52 (s, 1H), 8.07 (dd, *J* = 6.6, 2.6 Hz, 1H), 7.81 (dt, *J* = 7.3, 3.4 Hz, 1H), 7.51 (t, *J* = 9.8 Hz, 1H), 5.77 (s, 1H), 4.68 (s, 2H), 4.58 (s, 1H), 4.33-4.24 (m, 1H), 3.03-2.86 (m, 2H), 2.30-2.14 (m, 2H), 2.05-1.78 (m, 2H), 1.67 (s, 3H), 1.64-1.23 (m, 14H), 1.13 (td, *J* = 12.4, 3.9 Hz, 2H), 1.02 (m, 1H), 0.96 (s, 3H), 0.87 (d, *J* = 12.8 Hz, 7H), 0.76 (s, 3H), 0.67 (s, 4H). 13C NMR (101 MHz, DMSO-d6) δ174.80, 166.06, 155.61, 150.05, 135.30, 124.99, ,117.86, ,116.97, 116.33, 109.62, 76.73, 62.06, 55.76, 54.85, 51.63, 49.88, 48.72, 46.44, 41.96, 40.21, 38.43, 38.22, 37.43, 36.65, 36.15, 33.84, 31.32, 29.88, 29.02, 28.02, 27.10, 24.99, 20.36, 18.86, 17.85, 15.79, 15.70, 15.47, 14.30. HRMS (ESI) m/z: calculated $C_{39}H_{52}F_4NO_4^-$ [M-H]$^-$ 674.3838, found: 674.3756.

2-((2-Bromo-4-fluorophenyl)amino)-2-oxoethyl(1*R*,3a*S*,5a*R*,5b*R*,7a*R*,9*S*,11a*R*,11b*R*,13a*R*,13b*R*)-9-hydroxy-5a,5b,8,8,11a-pentamethyl-1-(prop-1-en-2-yl)icosahydro-3a*H*-cyclopenta[*a*]chrysene-3a-carboxylate (**3e**)

White solid, yield 60%. ^{1}H NMR (400 MHz, DMSO-*d*$_6$) δ 9.64 (s, 1H), 7.64 (td, *J* = 9.4, 8.9, 4.3 Hz, 2H), 7.28 (td, *J* = 8.6, 2.9 Hz, 1H), 4.79-4.64 (m, 3H), 4.56 (s, 1H), 4.25 (d, *J* = 5.1 Hz, 1H), 3.01-2.86 (m, 2H), 2.21 (dd, *J* = 18.1, 9.2 Hz, 2H), 1.99 (s, 2H), 1.65 (s, 3H), 1.62-1.22 (m, 14H), 1.20-1.10 (m, 2H), 1.00 (m, 1H), 0.94 (s, 3H), 0.86 (d, *J* = 8.0 Hz, 7H), 0.75 (s, 3H), 0.65 (s, 4H). 13C NMR (101 MHz, DMSO-*d*$_6$) δ 174.53, 166.08, 150.07, 132.29, 127.94, 119.67, 119.42, 115.16, 114.94, 109.63, 76.72, 61.78, 55.81, 54.85, 49.87, 48.71, 46.44, 41.96, 40.21, 38.43, 38.21, 37.41, 36.66, 36.17, 33.82, 31.29, 29.85, 29.06, 28.03, 27.10, 25.00, 20.36, 18.86, 17.88, 15.86, 15.72, 15.60. HRMS (ESI) m/z: calculated $C_{38}H_{52}BrFNO_4^-$ [M-H]$^-$ 684.3069, found: 684.2917.

2-((3-Bromo-4-fluorophenyl)amino)-2-oxoethyl(1*R*,3a*S*,5a*R*,5b*R*,7a*R*,9*S*,11a*R*,11b*R*,13a*R*,13b*R*)-9-hydroxy-5a,5b,8,8,11a-pentamethyl-1-(prop-1-en-2-yl)icosahydro-3a*H*-cyclopenta[*a*]chrysene-3a-carboxylate (**3f**)

White solid, yield 59%. ^{1}H NMR (400 MHz, DMSO-*d*$_6$) δ 10.33 (s, 1H), 7.99 (dd, *J* = 6.4, 2.5 Hz, 1H), 7.48 (ddd, *J* = 9.0, 4.4, 2.6 Hz, 1H), 7.35 (t, *J* = 8.8 Hz, 1H), 4.68 (d, *J* = 2.2 Hz, 1H), 4.64 (d, *J* = 2.2 Hz, 2H), 4.56 (t, *J* = 2.0 Hz, 1H), 4.25 (d, *J* = 5.1 Hz, 1H), 2.93 (ddt, *J* = 31.0, 11.0, 5.4 Hz, 2H), 2.27-2.13 (m, 2H), 2.02-1.79 (m, 2H), 1.65 (s, 3H), 1.62-1.22 (m, 14H), 1.20-1.06 (m, 2H), 1.00 (m, 1H), 0.94 (s, 3H), 0.86 (d, *J* = 10.1 Hz, 7H), 0.75 (s, 3H), 0.65 (s, 4H). 13C NMR (101 MHz, DMSO-d6) δ174.78, 165.82, 155.39, 150.06, 135.96, 123.28, 119.99, 116.87, 109.63, 107.45, 76.73, 62.02, 55.76, 54.85, 49.87, 48.70, 46.44, 41.96, 40.21, 38.43, 38.21, 37.42, 36.66, 36.16, 33.83, 31.30, 29.87, 29.03, 28.03, 27.10, 24.99, 20.36, 18.88, 17.88, 15.84, 15.72, 15.53, 14.31. HRMS (ESI) m/z: calculated $C_{38}H_{52}BrFNO_4^-$ [M-H]$^-$ 684.3069, found: 684.3069.

2-((3-Chloro-2-fluorophenyl)amino)-2-oxoethyl(1*R*,3a*S*,5a*R*,5b*R*,7a*R*,9*S*,11a*R*,11b*R*,13a*R*,13b*R*)-9-hydroxy-5a,5b,8,8,11a-pentamethyl-1-(prop-1-en-2-yl)icosahydro-3a*H*-cyclopenta[*a*]chrysene-3a-carboxylate (**3g**)

White solid, yield 52%. ^{1}H NMR (400 MHz, DMSO-d_6) δ 10.10 (s, 1H), 7.87 (ddd, J = 8.4, 7.0, 1.6 Hz, 1H), 7.34 (ddd, J = 8.3, 6.8, 1.6 Hz, 1H), 7.20 (td, J = 8.2, 1.5 Hz, 1H), 4.74 (d, J = 3.1 Hz, 2H), 4.68 (d, J = 2.5 Hz, 1H), 4.59-4.54 (m, 1H), 4.25 (d, J = 5.1 Hz, 1H), 2.93 (ddq, J = 26.6, 11.0, 5.4, 4.9 Hz, 2H), 2.26-2.13 (m, 2H), 2.02-1.81 (m, 2H), 1.65 (s, 3H), 1.62-1.21 (m, 15H), 1.20-1.07 (m, 2H), 1.00 (m, 1H), 0.94 (s, 3H), 0.86 (d, J = 8.0 Hz, 6H), 0.75 (s, 3H), 0.65 (s, 4H). ^{13}C NMR (101 MHz, DMSO-d_6) δ 174.68, 166.25, 150.07, 147.77,127.10, 125.46, 125.02, 124.98, 122.42, 119.89, 109.62, 76.73, 61.88, 55.78, 54.85, 49.87, 48.69, 46.44, 41.96, 40.21, 38.43, 38.21, 37.42, 36.66, 36.18, 33.82, 31.30, 29.85, 29.03, 28.03, 27.11, 25.00, 20.36, 18.87, 17.89, 15.85, 15.72, 15.54, 14.31. HRMS (ESI) m/z: calculated $C_{38}H_{52}ClFNO_4$ $[M+Na]^+$ 664.3539, found: 664.3381.

2-((2-Chloro-3-fluorophenyl)amino)-2-oxoethyl(1*R*,3a*S*,5a*R*,5b*R*,7a*R*,9*S*,11a*R*,11b*R*,13a*R*,13b*R*)-9-hydroxy-5a,5b,8,8,11a-pentamethyl-1-(prop-1-en-2-yl)icosahydro-3a*H*-cyclopenta[*a*]chrysene-3a-carboxylate (**3h**)

White solid, yield 61%. ^{1}H NMR (400 MHz, DMSO-d_6) δ 9.81 (s, 1H), 7.66 (dd, J = 8.3, 1.5 Hz, 1H), 7.38 (td, J = 8.3, 6.2 Hz, 1H), 7.23 (td, J = 8.8, 1.4 Hz, 1H), 4.77 (d, J = 3.3 Hz, 2H), 4.69 (d, J = 2.5 Hz, 1H), 4.56 (d, J = 2.2 Hz, 1H), 4.25 (d, J = 5.1 Hz, 1H), 2.93 (ddt, J = 27.4, 11.0, 5.4 Hz, 2H), 2.27-2.14 (m, 2H), 2.04-1.94 (m, 1H), 1.93-1.78 (m, 1H), 1.65 (s, 3H), 1.62-1.22 (m, 14H), 1.21-1.07 (m, 2H), 1.00 (m, 1H), 0.94 (s, 3H), 0.86 (d, J = 7.6 Hz, 7H), 0.75 (s, 3H), 0.65 (s, 4H). ^{13}C NMR (101 MHz, DMSO-d_6) δ 174.61, 166.26,156.46, 150.07, 136.25, 128.10, 120.61, 112.64, 112.43, 109.64, 76.73, 61.93, 55.80, 54.84, 49.87, 48.70, 46.44, 41.97, 40.21, ,38.43, 38.21, 37.42, 36.66, 36.18, 33.82, 31.29, 29.85, 29.05, 28.03, 27.11, 25.00, 20.36, 18.87, 17.88, 15.85, 15.72, 15.57, 14.32. HRMS (ESI) m/z: calculated $C_{38}H_{52}ClFNO_4$ $[M+Na]^+$ 664.3539, found: 664.3456.

2-((4-Methoxyphenyl)amino)-2-oxoethyl(1*R*,3a*S*,5a*R*,5b*R*,7a*R*,9*S*,11a*R*,11b*R*,13a*R*,13b*R*)-9-hydroxy-5a,5b,8,8,11a-pentamethyl-1-(prop-1-en-2-yl)icosahydro-3a*H*-cyclopenta[*a*]chrysene-3a-carboxylate (**3i**)

White solid, yield 61%. ^{1}H NMR (400 MHz, DMSO-d_6) δ 9.95 (s, 1H), 7.50-7.42 (m, 2H), 6.93-6.84 (m, 2H), 4.68 (d, J = 2.5 Hz, 1H), 4.61 (d, J = 1.9 Hz, 2H), 4.56 (q, J = 2.9, 2.1 Hz, 1H), 4.26 (s, 1H), 3.72 (s, 3H), 3.01-2.84 (m, 2H), 2.27-2.14 (m, 2H), 2.03-1.79 (m, 2H), 1.65 (s, 3H), 1.62-1.22 (m, 14H), 1.13 (dd, J = 22.9, 8.5 Hz, 2H), 1.00 (m, 1H),0.94 (s, 3H), 0.86 (d, J = 7.5 Hz, 7H), 0.75 (s, 3H), 0.65 (s, 4H). ^{13}C NMR (101 MHz, DMSO-d_6) δ 174.73, 164.98, 155.20, 150.11, 131.74, 120.55, 113.86, 109.60, 76.73, 62.00, 55.77, 55.10, 54.86, 49.89, 48.71, 46.45, 41.97, 40.22, 38.44, 38.22, 37.42, 36.66, 36.21, 33.83, 31.33, 29.88, 29.04, 28.04, 27.11, 25.01, 20.37, 18.88, 17.90, 15.87, 15.73, 15.56, 14.33. HRMS (ESI) m/z: calculated $C_{39}H_{56}NO_5^-$ $[M-H]^-$ 618.4164, found: 618.4119.

2-((2,3-Difluorophenyl)amino)-2-oxoethyl(1*R*,3a*S*,5a*R*,5b*R*,7a*R*,9*S*,11a*R*,11b*R*,13a*R*,13b*R*)-9-hydroxy-5a,5b,8,8,11a-pentamethyl-1-(prop-1-en-2-yl)icosahydro-3a*H*-cyclopenta[*a*]chrysene-3a-carboxylate (**3j**)

White solid, yield 65%. ^{1}H NMR (400 MHz, DMSO-d_6) δ 10.14 (s, 1H), 7.88 (ddd, J = 10.1, 6.2, 3.2 Hz, 1H), 7.33 (td, J = 9.8, 5.1 Hz, 1H), 6.98 (tt, J = 7.8, 3.3 Hz, 1H), 4.75 (d, J = 3.1 Hz, 2H), 4.68 (d, J = 2.5 Hz, 1H), 4.57 (s, 1H), 4.25 (d, J = 5.1 Hz, 1H), 2.93 (ddt, J = 27.9, 10.9, 5.4 Hz, 2H), 2.26-2.13 (m, 2H), 2.02-1.79 (m, 2H), 1.65 (s, 3H), 1.62-1.22 (m, 14H), 1.21-1.06 (m, 2H), 1.00 (m, 1H), 0.94 (s, 3H), 0.87 (d, J = 3.9 Hz, 7H), 0.75 (s, 3H), 0.65 (s, 4H). ^{13}C NMR (101 MHz, DMSO-d_6) δ 174.71, 166.51, 158.79, 150.06, ,126.92, 116.41, 116.29, 110.72, 109.45, 109.16, 76.74, 61.93, 55.77, 54.86, 49.89, 48.70, 46.44, 41.97, 40.23, 38.44, 38.23, 37.44, 36.66, 36.19, 33.84, 31.32, 29.87, 29.03, 28.03, 27.11, 25.00, 20.37, 18.87, 17.90, 15.84, 15.71, 15.54, 14.31. HRMS (ESI) m/z: calculated $C_{38}H_{52}F_2NO_4^-$ [M-H]$^-$ 624.3870, found: 624.3771.

2-Oxo-2-((4-(trifluoromethyl)phenyl)amino)ethyl(1*R*,3a*S*,5a*R*,5b*R*,7a*R*,9*S*,11a*R*,11b*R*,13a*R*,13b*R*)-9-hydroxy-5a,5b,8,8,11a-pentamethyl-1-(prop-1-en-2-yl)icosahydro-3a*H*-cyclopenta[*a*]chrysene-3a-carboxylate (**3k**)

White solid, yield 65%. ^{1}H NMR (400 MHz, DMSO-d_6) δ 10.48 (s, 1H), 7.77 (d, J = 8.6 Hz, 2H), 7.69 (d, J = 8.6 Hz, 2H), 4.68 (d, J = 4.2 Hz, 3H), 4.57 (d, J = 2.3 Hz, 1H), 4.24 (d, J = 5.1 Hz, 1H), 2.93 (ddq, J = 26.8, 11.1, 5.5, 4.9 Hz, 2H), 2.27-2.14 (m, 2H), 2.00 (q, J = 7.1, 6.0 Hz, 1H), 1.90-1.80 (m, 1H), 1.65 (s, 3H), 1.62-1.22 (m, 14H), 1.21-1.06 (m, 2H), 1.00 (m, 1H), 0.94 (s, 3H), 0.86 (d, J = 13.2 Hz, 7H), 0.74 (s, 3H), 0.65 (s, 4H). ^{13}C NMR (101 MHz, DMSO-d_6) δ 174.77, 166.19, 150.06, 142.08, 126.08, 123.21, 118.90, 109.62, 76.74, 62.07, 59.67, 55.78, 54.85, 49.88, 48.69, 46.44, 41.96, 40.20, 38.43, 38.22, 37.41, 36.66, 36.19, 33.82, 31.30, 29.87, 29.04, 28.03, 27.11, 24.99, 20.36, 18.87, 17.88, 15.85, 15.70, 15.51, 14.30. HRMS (ESI) m/z: calculated $C_{39}H_{53}F_3NO_4^-$ [M-H]$^-$ 656.3932, found: 656.3960.

2-((4-Cyanophenyl)amino)-2-oxoethyl(1*R*,3a*S*,5a*R*,5b*R*,7a*R*,9*S*,11a*R*,11b*R*,13a*R*,13b*R*)-9-hydroxy-5a,5b,8,8,11a-pentamethyl-1-(prop-1-en-2-yl)icosahydro-3a*H*-cyclopenta[*a*]chrysene-3a-carboxylate (**3l**)

White solid, yield 59%. ^{1}H NMR (400 MHz, DMSO-d_6) δ 10.58 (s, 1H), 7.86-7.72 (m, 4H), 4.70 (d, J = 4.9 Hz, 3H), 4.58 (s, 1H), 4.26 (d, J = 5.1 Hz, 1H), 2.95 (ddt, J = 26.6, 11.0, 5.4 Hz, 2H), 2.22 (ddd, J = 24.2, 11.3, 3.2 Hz, 2H), 2.05-1.81 (m, 2H), 1.67 (s, 3H), 1.65-1.24 (m, 14H), 1.23-1.08 (m, 2H), 1.02 (m, 1H), 0.96 (s, 3H), 0.88 (d, J = 10.8 Hz, 7H), 0.77 (s, 3H), 0.67 (s, 4H). ^{13}C NMR (101 MHz, DMSO-d_6) δ 174.77, 166.37, 150.05, 142.69, 133.32, 119.06, 109.63, 105.13, 76.74, 62.07, 59.67, 55.78, 54.86, 49.88, 48.68, 46.43, 41.97, 40.21, 38.44, 38.22, 37.42, 36.66, 36.19, 33.83, 31.29, 29.86, 29.03, 28.03, 27.11, 25.00, 20.36, 18.88, 17.90, 15.86, 15.72, 15.52, 14.32. HRMS (ESI) m/z: calculated $C_{39}H_{53}N_2O_4^-$ [M-H]$^-$ 613.4011, found: 613.3936.

2-((3-Chlorophenyl)amino)-2-oxoethyl(1*R*,3a*S*,5a*R*,5b*R*,7a*R*,9*S*,11a*R*,11b*R*,13a*R*,13b*R*)-9-hydroxy-5a,5b,8,8,11a-pentamethyl-1-(prop-1-en-2-yl)icosahydro-3a*H*-cyclopenta[*a*]chrysene-3a-carboxylate (**3m**)

White solid, yield 62%. ^{1}H NMR (400 MHz, DMSO-d_6) δ 10.32 (s, 1H), 7.76 (t, J = 2.0 Hz, 1H), 7.45-7.31 (m, 2H), 7.13 (ddd, J = 7.8, 2.1, 1.1 Hz, 1H), 4.67 (dd, J = 12.0, 2.4 Hz, 3H), 4.56 (t, J = 2.0 Hz, 1H), 4.26 (d, J = 5.2 Hz, 1H), 2.93 (ddt, J = 30.1,

11.0, 5.4 Hz, 2H), 2.20 (ddd, *J* = 24.0, 11.6, 3.1 Hz, 2H), 2.04-1.78 (m, 2H), 1.65 (s, 3H), 1.63-1.22 (m, 14H), 1.20-1.07 (m, 2H), 1.00 (dd, *J* = 13.1, 4.6 Hz, 1H), 0.94 (s, 3H), 0.86 (d, *J* = 8.2 Hz, 7H), 0.75 (s, 3H), 0.65 (s, 4H). ^{13}C NMR (101 MHz, DMSO-d_6) *δ* 174.79, 165.93, 150.06, 139.92, 133.07, 130.47, 123.07, 118.56, 117.43, 109.63, 76.74, 62.06, 55.77, 54.86, 49.89, 48.71, 46.44, 41.97, 40.22, 38.44, 38.23, 37.43, 36.66, 36.18, 33.84, 31.32, 29.88, 29.04, 28.04, 27.11, 25.00, 20.37, 18.88, 17.89, 15.85, 15.72, 15.54, 14.32. HRMS (ESI) m/z: calculated $C_{38}H_{53}ClNO_4^-$ [M-H]$^-$ 622.3669, found: 622.3591.

2-((2-Chlorophenyl)amino)-2-oxoethyl(1*R*,3a*S*,5a*R*,5b*R*,7a*R*,9*S*,11a*R*,11b*R*,13a*R*,13b*R*)-9-hydroxy-5a,5b,8,8,11a-pentamethyl-1-(prop-1-en-2-yl)icosahydro-3a*H*-cyclopenta[*a*]chrysene-3a-carboxylate (**3n**)

White solid, yield 62%. ^{1}H NMR (400 MHz, DMSO-d_6) *δ* 9.63 (s, 1H), 7.77 (dd, *J* = 8.1, 1.6 Hz, 1H), 7.51 (dd, *J* = 8.1, 1.5 Hz, 1H), 7.34 (td, *J* = 7.8, 1.5 Hz, 1H), 7.21 (dd, *J* = 7.7, 1.6 Hz, 1H), 4.74 (d, *J* = 3.4 Hz, 2H), 4.68 (s, 1H), 4.56 (t, *J* = 2.0 Hz, 1H), 4.26 (d, *J* = 5.1 Hz, 1H), 2.93 (ddt, *J* = 26.6, 11.0, 5.4 Hz, 2H), 2.21 (ddd, *J* = 15.6, 10.3, 3.2 Hz, 2H), 2.04-1.79 (m, 2H), 1.65 (s, 3H), 1.62-1.21 (m, 14H), 1.21-1.07 (m, 2H), 1.04-0.96 (m, 1H), 0.94 (s, 3H), 0.86 (d, *J* = 7.0 Hz, 7H), 0.75 (s, 3H), 0.65 (s, 4H). ^{13}C NMR (101 MHz, DMSO-d_6) *δ* 174.57, 166.04, 150.07, 134.25, 129.47, 127.47, 126.19, 125.72, 125.33, 109.64, 76.74, 61.96, 55.81, 54.85, 49.88, 48.71, 46.45, 41.97, 40.21, 38.44, 38.22, 37.43, 36.66, 36.19, 33.83, 31.31, 29.86, 29.08, 28.04, 27.11, 25.00, 20.37, 18.87, 17.89, 15.86, 15.72, 15.59, 14.32. HRMS (ESI) m/z: calculated $C_{38}H_{53}ClNO_4^-$ [M-H]$^-$ 622.3669, found: 622.3474.

2-((4-Fluorophenyl)amino)-2-oxoethyl(1*R*,3a*S*,5a*R*,5b*R*,7a*R*,9*S*,11a*R*,11b*R*,13a*R*,13b*R*)-9-hydroxy-5a,5b,8,8,11a-pentamethyl-1-(prop-1-en-2-yl)icosahydro-3a*H*-cyclopenta[*a*]chrysene-3a-carboxylate (**3o**)

White solid, yield 62%. ^{1}H NMR (400 MHz, DMSO-d_6) *δ* 10.16 (s, 1H), 7.57 (dd, *J* = 8.9, 5.0 Hz, 2H), 7.16 (t, *J* = 8.9 Hz, 2H), 4.68 (d, *J* = 2.5 Hz, 1H), 4.64 (d, *J* = 2.0 Hz, 2H), 4.56 (d, *J* = 2.5 Hz, 1H), 4.26 (d, *J* = 5.1 Hz, 1H), 2.93 (ddt, *J* = 29.3, 11.1, 5.4 Hz, 2H), 2.21 (qd, *J* = 12.2, 10.8, 3.3 Hz, 2H), 2.05-1.79 (m, 2H), 1.65 (s, 3H), 1.62-1.22 (m, 14H), 1.21-1.06 (m, 2H), 1.00 (dd, *J* = 13.0, 4.7 Hz, 1H), 0.94 (s, 3H), 0.86 (d, *J* = 8.3 Hz, 7H), 0.75 (s, 3H), 0.65 (s, 4H). ^{13}C NMR (101 MHz, DMSO-d_6) *δ* 174.78, 165.44, 156.79, 150.10, 134.93, 120.80, 120.72, 115.45, 115.23, 109.64, 76.74, 62.00, 55.78, 54.86, 49.88, 48.70, 46.46, 41.98, 40.22, 38.45, 38.22, 37.42, 36.67, 36.20, 33.83, 31.31, 29.87, 29.05, 28.05, 27.12, 25.01, 20.37, 18.89, 17.91, 15.88, 15.74, 15.55, 14.33. HRMS (ESI) m/z: calculated $C_{38}H_{53}FNO_4^-$ [M-H]$^-$ 606.3964, found: 606.4013.

2-Oxo-2-(thiazol-2-ylamino)ethyl(1*R*,3a*S*,5a*R*,5b*R*,7a*R*,9*S*,11a*R*,11b*R*,13a*R*,13b*R*)-9-hydroxy-5a,5b,8,8,11a-pentamethyl-1-(prop-1-en-2-yl)icosahydro-3a*H*-cyclopenta[a]chrysene-3a-carboxylate (**3p**)

White solid, yield 58%. ^{1}H NMR (400 MHz, DMSO-d_6) *δ* 12.32 (s, 1H), 7.48 (d, *J* = 3.6 Hz, 1H), 7.23 (d, *J* = 3.6 Hz, 1H), 4.77 (s, 2H), 4.68 (d, *J* = 2.5 Hz, 1H), 4.56 (t, *J* = 2.1 Hz, 1H), 4.25 (d, *J* = 5.1 Hz, 1H), 2.93 (ddt, *J* = 31.5, 11.0, 5.4 Hz, 2H),

2.26-2.12 (m, 2H), 1.98-1.78 (m, 2H), 1.65 (s, 3H), 1.63-1.22 (m, 14H), 1.17 (t, *J* = 7.1 Hz, 2H), 1.05-0.97 (m, 1H), 0.94 (s, 3H), 0.87 (d, *J* = 5.0 Hz, 7H), 0.75 (s, 3H), 0.65 (s, 4H). ^{13}C NMR (101 MHz, DMSO-d_6) δ 174.78, 165.76, 157.42, 150.04, 137.59, 113.63, 109.65, 76.74, 61.42, 55.79, 54.86, 49.89, 48.70, 46.45, 41.98, 40.24, 38.44, 38.23, 37.47, 36.67, 36.17, 33.86, 31.32, 29.86, 29.02, 28.04, 27.11, 25.01, 20.37, 18.87, 17.92, 15.88, 15.72, 15.58, 14.32. HRMS (ESI) m/z: calculated $C_{35}H_{51}N_2O_4S^-$ [M-H]$^-$ 595.3575, found: 595.3510.

2-((5-Methylpyridin-2-yl)amino)-2-oxoethyl(1*R*,3a*S*,5a*R*,5b*R*,7a*R*,9*S*,11a*R*,11b*R*,13a*R*,13b*R*)-9-hydroxy-5a,5b,8,8,11a-pentamethyl-1-(prop-1-en-2-yl)icosahydro-3a*H*-cyclopenta[*a*]chrysene-3a-carboxylate (**3q**)

White solid, yield 61%. ^{1}H NMR (400 MHz, DMSO-d_6) δ 10.55 (s, 1H), 8.17 (d, *J* = 5.1 Hz, 1H), 7.85 (s, 1H), 6.95 (d, *J* = 5.1 Hz, 1H), 4.75 (d, *J* = 14.9 Hz, 1H), 4.71-4.64 (m, 2H), 4.56 (s, 1H), 4.26 (d, *J* = 5.1 Hz, 1H), 2.93 (ddt, *J* = 28.0, 11.0, 5.5 Hz, 2H), 2.31 (s, 3H), 2.25-2.11 (m, 2H), 2.06-1.81 (m, 2H), 1.65 (s, 3H), 1.60-1.22 (m, 14H), 1.20-1.08 (m, 2H), 1.00 (dd, *J* = 13.3, 4.2 Hz, 1H), 0.94 (s, 3H), 0.87 (d, *J* = 4.2 Hz, 7H), 0.75 (s, 3H), 0.65 (s, 4H). ^{13}C NMR (101 MHz, DMSO-d_6) δ 174.72, 166.28, 151.55, 150.09, 148.88, 147.58, 120.46, 113.76, 109.61, 76.74, 61.94, 55.77, 54.86, 49.90, 48.70, 46.47, 41.98, 40.23, 38.44, 38.23, 37.45, 36.67, 36.22, 33.85, 31.35, 29.91, 29.04, 28.03, 27.11, 26.29, 25.01, 20.37, 18.88, 17.90, 15.84, 15.72, 15.61, 14.32. HRMS (ESI) m/z: calculated $C_{38}H_{57}N_2O_4^+$ [M+H]$^+$ 605.4313, found: 605.4224.

2-(4-(4-Chlorophenyl)piperazin-1-yl)-2-oxoethyl(1*R*,3a*S*,5a*R*,5b*R*,7a*R*,9*S*,11a*R*,11b*R*,13a*R*,13b*R*)-9-hydroxy-5a,5b,8,8,11a-pentamethyl-1-(prop-1-en-2-yl)icosahydro-3a*H*-cyclopenta[*a*]chrysene-3a-carboxylate (**3r**)

White solid, yield 47%. ^{1}H NMR (400 MHz, DMSO-d_6) δ 7.25 (d, *J* = 8.5 Hz, 2H), 6.97 (d, *J* = 8.6 Hz, 2H), 4.91-4.76 (m, 2H), 3.31 (s, 2H), 3.22-3.06 (m, 4H), 2.93 (ddt, *J* = 28.0, 11.1, 5.5 Hz, 2H), 2.26-2.14 (m, 2H), 2.06-1.85 (m, 2H), 1.65 (s, 3H), 1.62-1.21 (m, 15H), 1.19-1.04 (m, 2H), 1.00 (m, 1H), 0.93 (s, 3H), 0.86 (d, *J* = 4.9 Hz, 7H), 0.75 (s, 3H), 0.65 (s, 4H). ^{13}C NMR (101 MHz, DMSO-d_6) δ 174.50, 164.96, 150.16, 149.48, 128.63, 122.82, 117.25, 109.57, 76.74, 60.66, 55.79, 54.86, 49.89, 48.71, 48.24, 47.89, 46.50, 43.38, 41.97, 40.88, 40.22, 38.44, 38.23, 37.40, 36.66, 36.31, 33.86, 31.40, 29.87, 29.01, 28.04, 27.12, 25.02, 20.40, 18.88, 17.91, 15.87, 15.73, 14.32. HRMS (ESI) m/z: calculated $C_{42}H_{62}ClN_2O_4^+$ [M+H]$^+$ 693.4393, found: 693.4311.

2-(4-(2,3-Dichlorophenyl)piperazin-1-yl)-2-oxoethyl(1*R*,3a*S*,5a*R*,5b*R*,7a*R*,9*S*,11a*R*,11b*R*,13a*R*,13b*R*)-9-hydroxy-5a,5b,8,8,11a-pentamethyl-1-(prop-1-en-2-yl)icosahydro-3a*H*-cyclopenta[*a*]chrysene-3a-carboxylate (**3s**)

White solid, yield 45%. ^{1}H NMR (400 MHz, DMSO-d_6) δ 7.37-7.29 (m, 2H), 7.19-7.12 (m, 1H), 4.85 (q, *J* = 14.8 Hz, 2H), 4.68 (s, 1H), 4.56 (s, 1H), 4.25 (d, *J* = 5.1 Hz, 1H), 3.56 (s, 4H), 2.98 (d, *J* = 15.0 Hz, 4H), 2.95-2.84 (m, 2H), 2.01 (d, *J* = 13.7 Hz, 2H), 1.65 (s, 3H), 1.61-1.24 (m, 14H), 1.19-1.06 (m, 2H), 0.99 (m, 1H), 0.94 (s, 3H), 0.87 (s, 7H), 0.76 (s, 3H), 0.65 (s, 4H). ^{13}C NMR (101 MHz, DMSO-d_6) δ 175.60, 165.44, 150.65, 150.55, 134.33, 127.90, 127.66, 125.44, 118.88, 109.63, 79.05, 60.76,

56.74, 55.45, 51.58, 51.08, 50.68, 49.58, 46.93, 44.97, 42.55, 42.20, 40.84, 38.94, 38.82, 38.23, 37.29, 37.14, 34.41, 32.12, 30.66, 29.72, 28.07, 27.50, 25.64, 20.99, 19.50, 18.39, 16.21, 16.12, 15.44, 14.76. HRMS (ESI) m/z: calculated $C_{42}H_{61}Cl_2N_2O_4^+$ $[M+H]^+$ 727.4003, found: 727.4087.

2-(4-(4-Nitrophenyl)piperazin-1-yl)-2-oxoethyl(1*R*,3a*S*,5a*R*,5b*R*,7a*R*,9*S*,11a*R*,11b*R*,13a*R*,13b*R*)-9-hydroxy-5a,5b,8,8,11a-pentamethyl-1-(prop-1-en-2-yl)icosahydro-3a*H*-cyclopenta[*a*]chrysene-3a-carboxylate (**3t**)

White solid, yield 45%. ^{1}H NMR (400 MHz, DMSO-*d*$_6$) δ 8.09 (d, J = 9.4 Hz, 2H), 7.08-7.00 (m, 2H), 4.94-4.79 (m, 2H), 4.69 (d, J = 2.6 Hz, 1H), 4.58 (d, J = 2.5 Hz, 1H), 4.26 (d, J = 5.1 Hz, 1H), 3.52 (s, 2H), 3.33 (s, 3H), 2.94 (ddt, J = 27.6, 11.0, 5.4 Hz, 2H), 2.26-2.16 (m, 2H), 2.07-1.98 (m, 1H), 1.96-1.87 (m, 1H), 1.66 (s, 3H), 1.63-1.22 (m, 14H), 1.09 (d, J = 12.4 Hz, 2H), 1.00 (m, 1H),0.95 (s, 3H), 0.88 (d, J = 4.9 Hz, 7H), 0.76 (s, 3H), 0.66 (s, 4H). ^{13}C NMR (101 MHz, DMSO-*d*$_6$) δ 174.51, 165.25, 154.27, 150.16, 137.01, 125.65, 112.56, 109.58, 76.74, 60.65, 55.80, 54.86, 49.89, 48.70, 46.51, 45.89, 45.75, 42.82, 41.97, 40.54, 40.22, 38.44, 38.23, 37.41, 36.66, 36.32, 33.86, 31.40, 29.88, 29.00, 28.04, 27.12, 25.02, 20.39, 18.88, 17.90, 15.86, 15.73, 14.32. HRMS (ESI) m/z: calculated $C_{42}H_{62}N_3O_6^+$ $[M+H]^+$ 704.4633, found: 704.4661.

2. Effect of the solvent DMSO on biofilms.

To assess the potential influence of the solvent dimethyl sulfoxide (DMSO) on the experimental outcomes, a solvent control group was established under identical conditions. All test compounds were dissolved in DMSO and diluted with culture medium to the desired working concentrations prior to the experiments. The final concentration of DMSO in the experimental systems was 1% (v/v). The results demonstrated that, compared with the blank control group containing no solvent, DMSO at the above concentration did not exert any significant effect (P > 0.05) on the planktonic growth or the biofilm-forming capacity (quantified by crystal violet staining) of the tested bacteria. Therefore, the antibacterial activity and biofilm inhibition observed in this study can be attributed to the test compounds themselves rather than to the solvent DMSO.

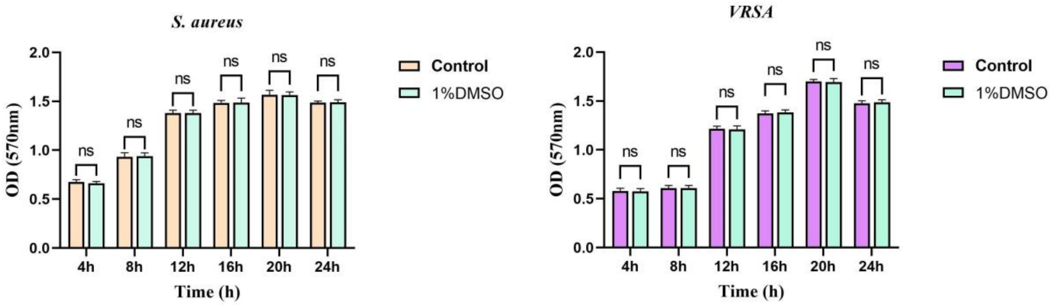

Figure S21 Effect of the solvent DMSO on the biofilms of two *S. aureus* strains.
